# msqrob2TMT: Robust Linear Mixed Models for Inferring Differential Abundant Proteins in Labeled Experiments With Arbitrarily Complex Design

**DOI:** 10.1016/j.mcpro.2025.101002

**Published:** 2025-05-30

**Authors:** Stijn Vandenbulcke, Christophe Vanderaa, Oliver Crook, Lennart Martens, Lieven Clement

**Affiliations:** 1 Department of Mathematics, Computer Science and Statistics, Ghent University, Ghent, Belgium; 2 CompOmics, VIB Center for Medical Biotechnology, VIB, Ghent, Belgium; 3 Department of Biomolecular Medicine, Faculty of Medicine and Health Sciences, Ghent University, Ghent, Belgium; 4 Department of Statistics, University of Oxford, Oxford, UK; 5 BioOrganic Mass Spectrometry Laboratory (LSMBO), IPHC UMR 7178, University of Strasbourg, CNRS, Strasbourg, France; 6 Infrastructure Nationale de Protéomique ProFI − FR2048, Strasbourg, France

**Keywords:** differential proteomics, mass spectrometry, TMT labeling, mixed models, statistics

## Abstract

Labeling strategies in mass spectrometry–based proteomics enhance sample throughput by enabling the acquisition of multiplexed samples within a single run. However, contemporary experiments often involve increasingly complex designs, where the number of samples exceeds the capacity of a single run, resulting in a complex correlation structure that must be addressed for accurate statistical inference and reliable biomarker discovery. To this end, we introduce msqrob2TMT, a suite of mixed model-based workflows specifically designed for differential abundance analysis in labeled mass spectrometry–based proteomics data. msqrob2TMT accommodates both sample-specific and feature-specific (e.g., peptide or protein) covariates, facilitating inference in experiments with arbitrarily complex designs and allowing for explicit correction of feature-specific covariates. We benchmark our innovative workflows against state-of-the-art tools, including DEqMS, MSstatsTMT, and msTrawler, using two spike-in studies. Our findings demonstrate that msqrob2TMT offers greater flexibility, improved modularity, and enhanced performance, particularly through the application of robust ridge regression. Finally, we demonstrate the practical relevance of msqrob2TMT in a real mouse study, highlighting its capacity to effectively account for the complex correlation structure in the data.

## Highlights

• Statistical workflows for differential abundance analysis in labeled proteomics.
• Enhanced sensitivity and specificity.
• Flexible modeling for experiments with both simple and complex designs.
• Benchmarked against DEqMS, MSstatsTMT, and msTrawler using two spike-in datasets.
• Transparent and modular workflows through open-source Bioconductor package msqrob2.

## Graphical Abstract

**Figure:**
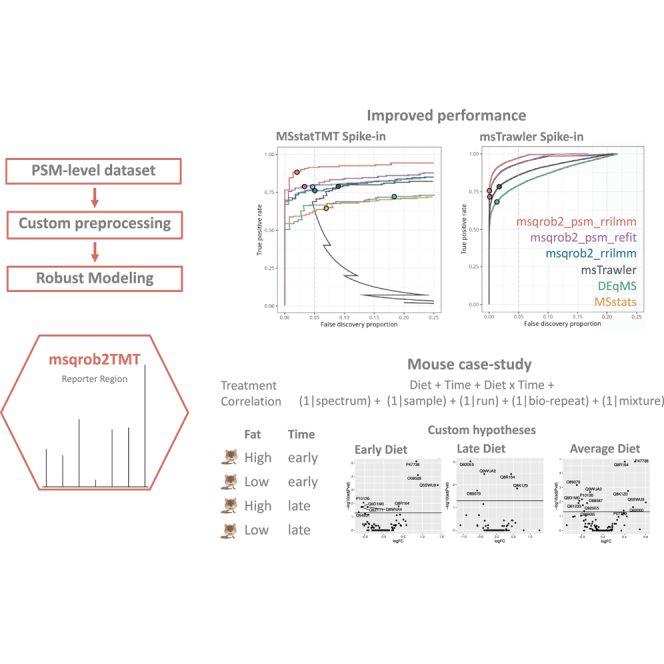

## In Brief

Novel msqrob2TMT workflows for differential abundance analysis of labeled proteomics data with simple and complex designs. Incorporating modular preprocessing and flexible modeling using multiple continuous and categorical variables and their interactions. Accounting for correlations within spectra, channels, runs, mixtures, and in longitudinal designs using random effects. Improved performance of our peptide-level and protein-level robust regression workflows over existing methods, demonstrated in two spike-in studies and one case study. Accessible through open-source Bioconductor package msqrob2 and companion GitHub page: https://github.com/statOmics/msqrob2tmt_paper.

High-throughput LC-MS–based proteomic workflows are widely used to quantify differential protein abundance across samples. Relative protein quantification is generally achieved through either label-free or labeled workflows. The latter employ stable isotope labeling techniques such as metabolic and postmetabolic labeling, which gained traction in recent years (1). Compared to label-free approaches, labeling offers the advantage of mitigating run-to-run variability in peptide identification and quantification by pooling and analyzing multiple samples within a single run. This enables researchers to compare proteomes across multiple conditions or treatments within a single mass spectrometry (MS) run, thereby providing a more comprehensive view of the proteome and enhancing the statistical power of the analysis. Current tandem mass tag (TMT) kits, for instance, support the multiplexing of up to 18 samples in a single run (1).

However, contemporary experiments often involve increasingly complex designs, where the number of samples exceeds the capacity of a single run. This has far reaching consequences for the downstream data analysis. In labeled experiments with multiple MS runs, biological replicates are often distributed across distinct runs, and additional technical MS runs are frequently included. Similar to label-free approaches, such multirun experiments are prone to numerous missing peptide intensity values across MS runs (2). Furthermore, the data of these designs are correlated at multiple levels. Indeed, the MS technology introduces various sources of technical variation, including run-level effects, channel effects, and spectrum-specific effects, among others. Hence, ion intensities from samples multiplexed in the same run are more similar than those from samples multiplexed in different runs. Additionally, quantification does not happen at the protein level but at the level of its identified peptide ions. Peptide ion abundances from the same sample within a run are inherently more similar than those of peptide ions from different samples. At the lowest level, TMT reporter ions from a specific spectrum enable peptide ion quantification across multiple samples, resulting in correlated ion intensities; ion intensities within the same spectrum are more alike than those measured across different spectra. Furthermore, mixtures of multiplexed samples are often run in technical repeats on the MS, introducing yet another level of correlation. Addressing such a complex correlation structure in labeled experiments necessitates flexible statistical models capable of producing valid tests for differential abundance. However, most proteomics software tools assume independence among the (summarized) ion intensities and can therefore not account for the abovementioned intricate correlation structures in contemporary TMT experiments, which may in turn lead to an inflation of false positives (FPs) (3). The MSstatsTMT tool (3), however, is one of the notable exceptions, as it has been explicitly developed to accommodate experiments with multiple conditions, biological replicate runs, technical repeat runs, and unbalanced designs.

In this publication, we present a bespoke data analysis workflow for labeled MS-based proteomics experiments, embedded in our msqrob2 universe in R/Bioconductor. Initially designed for label-free workflows, msqrob2 has demonstrated advantages over other state-of-the-art tools in terms of both performance and its modular, transparent implementation (4). In this work, we extend these benefits to the analysis of labeled experiments. Similar to MSstatsTMT, our workflows address the complex correlation structure inherent in contemporary labeled MS-based proteomics experiments by mixed models. However, unlike MSstatsTMT and msTrawler (5), we avoid by default the imputation of missing peptide-spectrum match (PSM) intensities, instead assuming missingness at random after accounting for peptide effects. Nevertheless, users retain the option in msqrob2TMT to utilize the default imputation strategies available in QFeatures or to implement custom functions tailored to their specific needs. Additionally, DEqMS (6) and MSstatsTMT model the data upon summarization at the protein level and msTrawler immediately models the data at the PSM level, while msqrob2TMT provides the user with the option to use either PSM-level or protein-level workflows. Another notable difference is that msqrob2 offers ridge regression and robust M-estimation, which can further stabilize parameter estimation and thus enhance reliable detection of differential proteins.

We demonstrate that our novel msqrob2TMT workflows outperform state-of-the-art tools by benchmarking them against DEqMS, MSstatsTMT, and msTrawler using two spike-in and real experimental dataset. Our comparisons also highlight that none of the state-of-the-art tools could provide valid inference across all of these three datasets, as their specific implementation limits the experimental designs for which they can infer differential abundance (DA). Particularly, MSstatsTMT cannot account for multiple covariates, for example, treatment effects together with block effects, age and/or other confounders, while DEqMS and msTrawler cannot address the complex correlation in TMT experiments with multiple runs and technical replication, which are commonly employed in the proteomics community. Note that Proteome Discoverer was not included in our benchmark, as we utilized datasets featured in the original MSstatsTMT publication, where the authors had already established the superior performance of MSstatsTMT compared to Proteome Discoverer (3).

Importantly, our novel msqrob2TMT workflows offer researchers extensive flexibility to develop both peptide-level and protein-level data analysis workflows, incorporating custom preprocessing steps and user-defined models that can be tailored to their specific applications. The latest release further expands support for complex experimental designs by providing full compatibility with the model specification capabilities of the popular lme4 R package for mixed models, while enabling the use of both feature-level and sample-level covariates. The sample-level covariates allow msqrob2 to accommodate longitudinal and clustered experimental designs. The use of feature-level covariates is supported through integration with the QFeatures (7) data infrastructure. This integration ensures that raw input data are never lost but remain accessible and linked to the preprocessed, normalized, and summarized assays, as well as to the model outputs. This approach guarantees transparency, traceability, and reproducibility throughout the analysis workflow.

## Experimental Procedures

In this section, we first introduce the datasets that are used to evaluate our novel workflows and to benchmark them against state-of-the-art methods DEqMS, MSstatsTMT, and msTrawler. Next, we briefly introduce these methods and our novel msqrob2TMT workflows. We conclude this section with the different metrics used to benchmark performance.

### Datasets

Three datasets are used to evaluate and benchmark the different tools: a spike-in dataset, a multibatch benchmarking experiment, and real mouse case study.

#### MSstatsTMT Spike-In Dataset

The spike-in dataset was obtained from MassIVE (RMSV000000265) and has the following design: 500, 333, 250, and 62.5 fmol of UPS1 peptides were spiked into 50 μg of stable isotope labeling by amino acids in cell culture (SILAC) HeLa peptides. This series forms a dilution gradient of 1, 0.667, 0.5, and 0.125 relative to the highest UPS1 peptide amount (500 fmol). A reference sample was prepared by combining the diluted UPS1 peptide samples (286.5 fmol) with 50 μg of SILAC HeLa peptides. Each dilution and the reference sample were processed in duplicate, yielding a total of 10 samples. These samples were subsequently labeled using TMT10-plex reagents and combined for LC-MS/MS analysis, hereafter referred to as a “mixture.” This protocol was repeated five times. Each mixture was analyzed in technical triplicate, resulting in a total of 15 MS runs. This experimental design simulates a scenario with 10 biological replicates per condition, consisting of two replicates per condition within each of the five mixtures. The MS data were searched by the MSstatsTMT authors using Proteome Discoverer 2.2.0.388 (Thermo Fisher Scientific, https://knowledge1.thermofisher.com/Software_and_Downloads/Chromatography_and_Mass_Spectrometry_Software/Proteome_Discoverer/Proteome_Discoverer_User_Guides/Proteome_Discoverer_2.2_overview) and Mascot Server 2.6.1 (Matrix Science).

A custom filtering step was required prior to the standard preprocessing performed by the different methods. In this study, two PSMs can exhibit identical intensity values due to the use of two separate Mascot search nodes: one targeting the SwissProt protein database and the other targeting the Sigma UPS protein database. This dual search strategy was employed to identify which PSMs originate from HeLa cell proteins, which are labeled with both SILAC and TMT, and which PSMs originate from the spike-in UPS proteins, labeled only with TMT. However, identification is imperfect, as some spectra are matched in both nodes, leading to different peptides being matched with the same spectrum and identical intensity values. These PSMs were excluded, as it is not possible to ascertain whether they correspond to the spike-in UPS proteins, the HeLa background proteins, or, in the extreme case, a mixture of both. Additionally, following the MSstatsTMT default settings, PSMs with fewer than six out of 10 intensity values were removed, and the PSM with the highest sum of intensity values is chosen when multiple spectra for the same peptide ion occur.

#### msTrawler Multibatch Benchmarking Experiment

The msTrawler multibatch benchmarking experiment data has been deposited on ProteomeXchange (PXD036799). However, for reproducibility of the results published in the original paper, the data available on the google drive of the msTrawler paper (https://console.cloud.google.com/storage/browser/mstrawler_paper) was used. The experiment is a spike-in study which consists of a constant mouse plasma background in which yeast proteins were then spiked with the following dilution ratios 1, 2, 3, 5, 11, 14, 18, 20, 24, 28, and 32. The yeast proteins were harvested from different media; glycerol + ammonium sulfate, galactose + urea, galactose + monosodium glutamate, galactose + ammonium sulfate, glucose + ammonium sulfate, glucose + monosodium glutamate, and glucose + urea. The samples were labeled using the TMTpro 18-plex and pooled in six mixtures, each containing 15 samples. Two of the remaining TMT channels are reference channels, one reference channel is the combination of one batch and the other reference channel consists of all batches. For this dataset, the ground truth is known: yeast proteins were spiked in the samples in different amounts, while mouse proteins were added as a constant background. Hence, any yeast protein that is returned as significant is a true positive (TP), and any mouse protein that is returned as significant is a FP.

The dataset imposes specific challenges for the normalization. Indeed, the majority of the proteins are DA, that is, all yeast peptides, and they are all DA in the same direction, which implies a violation of the normalization assumptions for most tools. To address this issue, the authors of msTrawler (5) developed a custom normalization workflow tailored specifically to this dataset. Particularly, they excluded mouse PSMs that correlate with the yeast dilution profile, that is, PSMs with a Pearson correlation coefficient higher than 0.25. Subsequently, PSMs with a summed signal-to-noise ratio below 20 were filtered out. Finally, the within sample geometric mean for the 50% most stable mouse PSMs in a run is used for normalization. However, it should be noted that such a procedure is not applicable in typical experimental contexts, as the set of non-DA proteins is generally unknown. For this study, we nevertheless applied this custom msTrawler preprocessing workflow to the dataset prior to conducting differential analysis to ensure that all tools are benchmarked on the same preprocessed dataset, thereby enabling a fair comparison of their performance.

#### Mouse Dataset

The mouse dataset was downloaded from MassIVE (RMSV000000264). In the study conducted by (8), 20 mice were divided into groups to investigate the effects of low-fat (LF) and high-fat (HF) diets. The experiment involves two factors: the type of diet (LF or HF), and the duration of the diet, categorized as either short term (8 weeks) or long term (18 weeks). This design resulted in four distinct groups corresponding to each combination of diet type and duration. Five mice, that is, biological replicates, were randomly assigned to each group and samples from their epididymal adipose tissue were randomly assigned to three TMT10-plex mixtures. Within each mixture, two reference channels were included, each containing pooled samples representing a range of peptides from all samples. Not all TMT channels were utilized, leading to an unbalanced experimental design. Finally, each TMT mixture was fractionated into nine parts and analyzed using synchronous precursor selection, culminating in a total of 27 MS runs.

### Tools for Differential Analysis

In this section, we first introduce the state-of-the-art methods DEqMS, MSstatsTMT, and msTrawler before developing our novel msqrob2 workflows for labeled experiments.

#### DEqMS

The DEqMS workflow (6) starts by $\log_{2}$-transforming the observed PSM intensities. The PSM intensities are then summarized into protein intensities by adopting the median sweep algorithm to the PSM data for each protein in each run, separately. This approach can be regarded as a one-step median polish that corrects for PSM-specific effects (spectra effects). The PSM-specific effect is shared across all TMT intensities from the same spectrum, which can be estimated by their corresponding median. Hence, the median sweep algorithm corrects TMT intensities for PSM-specific effects by subtracting their corresponding median. Subsequently, the spectrum-centered PSM intensities in each TMT channel for each protein are aggregated into protein expression values using median summarization. Finally, the protein summaries are normalized by subtracting the median of all summarized protein expression values within their corresponding channel.

Note that for the msTrawler multibatch benchmarking experiment, DEqMS starts from data that have been custom-filtered and normalized (see “msTrawler Multibatch Benchmarking Experiment”). Subsequently, the reference channels are removed, the data are $\log_{2}$-transformed, and summarized through a modified median sweep algorithm, which omits the normalization step of the protein summaries.

DEqMS uses conventional linear models to model the abundances protein by protein. Hence, it is unable to account for the complex correlation structures that arise in labeled experiments involving multiple biological replicates, which are multiplexed across several mixtures and may be acquired through multiple technical repeat runs. Nevertheless, DEqMS is well suited to analyze data from simpler labeled experiments that can be modeled as randomized complete block designs. This applies to cases where all samples are derived from distinct biological replicates and where all treatments are multiplexed within each MS run, allowing variation between runs to be addressed through the inclusion of a fixed run effect:

$$\begin{array}{c} y_{rc}=x_{rc}^{t}\beta +\beta_{r}^{\text{run}}+\epsilon_{rc}\\ \epsilon_{rc}∼N\left(0,\sigma^{2}\right) \end{array}$$

with $y_{rc}$ the $\log_{2}$-transformed and summarized protein intensity for TMT channel *c* in MS-run *r*, $x_{rc}^{t}$ the covariate pattern, for example, diet, time, condition, dilution, and/or media for the sample in TMT channel *c* of run *r* that is used to model the mean protein abundance upon correction for the run effect, β a vector of model parameters modelling the $\log_{2}$ fold changes (FCs) for each covariate, and $\beta_{r}^{\text{run}}$ the fixed block effect for run *r*, and $\epsilon_{rc}$ a normally distributed error term with mean 0 and variance $\sigma^{2}$, that is., $N\left(0,\sigma^{2}\right)$. Hence, DEqMS can model data of complex experiments as long as these can be parameterized as a randomized complete block design. However, it cannot accomodate experiments with clustered or longitudinal designs, nor for designs including technical repeats.

Statistical hypothesis testing builds on the moderated *t* tests and F-tests from the popular limma package. So users have the flexibility to infer DA using any linear combination of the model parameters ($L^{T}\beta$), which we also refer to as contrasts. DEqMS version 1.24 (9) from Bioconductor release 3.20 is used in our article.

#### MSstatsTMT

The default MSstatsTMT workflow (3) begins with the $\log_{2}$ transformation of the observed MS intensities, followed by median equalization across PSMs, TMT channels, and runs. Missing values are subsequently imputed using an accelerated failure time model. After imputation, PSM intensities are summarized to protein intensities using Tukey’s median polish. Finally, protein intensities are normalized against the reference by subtracting the median of the corresponding reference channel intensities.

Note that, for the msTrawler multibatch benchmarking experiment, MSstatsTMT starts from data that have been custom-filtered and normalized (see “msTrawler Multibatch Benchmarking Experiment”). Subsequently, the data are $\log_{2}$-transformed, imputed using an accelerated failure time model when missing, summarized, and normalized against the reference channels.

MSstatsTMT can fit linear mixed models to the normalized and summarized protein intensities to infer DA between *T* treatment groups in experimental designs involving multiple biological replicates, which are multiplexed with tags across several mixtures and quantified through multiple technical repeat MS runs. These models are fitted protein-by-protein and are specified as follows:

$$\begin{array}{c} y_{rcm}=\beta_{0}+\sum_{t=1}^{T}\beta_{t}^{\text{treat}}x_{rcmt}^{\text{treat}}+u_{r}^{\text{run}}+u_{m}^{\text{mix}}+u_{cm}^{\text{biorep}}+\epsilon_{rcm}\\ \sum_{t=1}^{T}\beta_{t}^{\text{treat}}=0\\ u_{r}^{\text{run}}∼N\left(0,\sigma_{\text{run}}^{2}\right)\\ u_{m}^{\text{mix}}∼N\left(0,\sigma_{\text{mix}}^{2}\right)\\ u_{cm}^{\text{biorep}}∼N\left(0,\sigma_{\text{biorep}}^{2}\right)\\ \epsilon_{rcm}∼N\left(0,\sigma^{2}\right) \end{array}$$

with $y_{rcm}$ the $\log_{2}$-transformed and summarized protein intensity for TMT tag *c* in mixture *m* in MS run *r*, $\beta_{0}$ the intercept or the overall mean, $\beta_{t}^{\text{treat}}$ the treatment effect for treatment *t*, $x_{rcmt}^{\text{treat}}$ a dummy variable that is $x_{rcmt}^{\text{treat}}=1$ if the biological replicate cm from TMT channel *c* of mixture *m* belongs to group *t* and $x_{rcmt}^{\text{treat}}=0$ otherwise, and $u_{r}^{\text{run}}$, $u_{m}^{\text{mix}}$, and $u_{cm}^{\text{biorep}}$ normally distributed random effects with mean 0 and variance $\sigma_{\text{run}}^{2}$, $\sigma_{\text{mixture}}^{2}$, and $\sigma_{\text{biorep}}^{2}$, respectively, and $\epsilon_{rcm}$ the normally distributed error term with mean 0 and variance $\sigma^{2}$. Note that the random effects model the hierarchical correlation structure in the data, that is, the correlation within runs, mixtures, and biological replicates.

Hence, MSstatsTMT can only perform inference on experiments where treatments can be modeled using a single factor with multiple groups and assumes a very specific correlation structure: technical repeats involve the replication of an entire mixture. Consequently, MSstatsTMT cannot support additional covariates such as blocking variables or age, nor additional random effects needed to address studies with clustered or longitudinal designs.

The variance components are estimated using restricted maximum likelihood (REML). If insufficient data are available to estimate the parameters of the full model, MSstatsTMT reduces the model to infer DA on as many proteins as possible.

MSstatsTMT performs statistical inference on linear combinations of group means (contrasts) using approximate *t* tests. For example, to test the difference between treatments *j* and *k*, it calculates: $\log_{2}\;FC_{j-k}^{\text{treat}}=\beta_{j}^{\text{treat}}-\beta_{k}^{\text{treat}}$. The user has the flexibility to perform all pairwise comparisons between groups or to focus on user-specified contrasts.

In addition, the default empirical Bayes step from (10) is employed to stabilise the estimation of variance of the errors by borrowing strength across proteins; and a Satterthwaite approximation is used to estimate the degrees of freedom for the approximate *t*-statistics.

MSstatsTMT version 2.14.1 (3) from Bioconductor release 3.20 was used in our article.

#### msTrawler

msTrawler (5) initially removes peptides with a summed signal-to-noise ratio below 20. Missing values are imputed based on the limit of reliability, defined as 0.01 times the total scan intensity. Scans containing fewer than three values above this limit are excluded. Samples are then normalized by subtracting the column medians of the subset of peptides with a SD lower than the median SD. All these values reflect the default settings of msTrawler for these arguments.

Note that for the msTrawler multibatch benchmarking experiment, msTrawler starts from data that have been custom-filtered and normalized (see “msTrawler Multibatch Benchmarking Experiment”). Subsequently, missing data are imputed based on the limit of reliability, defined as 0.01 times the total scan intensity and scans containing fewer than three values above this limit are excluded.

msTrawler builds a linear mixed model starting from peptide-level data, based on user-specified sample and covariate files (5). Specifically, msTrawler employs the following base model:

$$\begin{array}{c} y_{rcs}=x_{rc}^{t}\beta +u_{rc}^{\text{samp}}+\epsilon_{rcs}\\ u_{rc}^{\text{samp}}∼N\left(0,\sigma_{\text{samp}}^{2}\right)\\ \epsilon_{rcs}∼N\left(0,\sigma^{2}\right) \end{array}$$

with $y_{rcs}$ the $\log_{2}$-transformed and normalized intensity for the peptide ion of scan *s* of a protein from the sample with TMT tag *c* in MS run *r*, $x_{rc}^{t}$ the covariate pattern for the sample in TMT channel *c* of run *r*, β the vector of model parameters modeling the effect of each covariate, and $u_{rc}^{\text{samp}}$ a normally distributed random effect with mean 0 and variance $\sigma_{\text{samp}}^{2}$ modeling the correlation of PSMs from the same protein pool in a sample, and $\epsilon_{rcs}$ the normally distributed error term with mean 0 and variance $\sigma^{2}$. Hence, msTrawler does not address the correlation within scans, runs, and mixtures that are acquired in multiple technical repeats on the MS.

msTrawler further enables the use of reference samples by extending the response vector with the ion intensities for the reference samples $y_{rbs}$ with TMT tag *b*:

$$\begin{array}{c} y_{rbs}=\alpha_{s}+\epsilon_{rbs}\\ y_{rcs}=\alpha_{s}+x_{rc}^{t}\beta +u_{rc}^{\text{samp}}+\epsilon_{rcs} \end{array}$$

where $\alpha_{s}$ is a nuisance parameter that accounts for scan-to-scan variability.

The model parameters are estimated using weighted REML, where each ion intensity is weighted according to its signal-to-noise ratio.

The covariate pattern $x_{rc}^{t}$ for each sample is constructed from a user-specified sample and covariate files. The covariates can be of the type “factor”, that is, a factor with multiple levels; “continuous”, that is, a linear term; “time”, that is, a linear, quadratic, or cubic trend by specifying the degree of the polynomial, a factor with every time point as a level, or a sine and cosine term to model circadian effects.

When multiple covariates are used, these are added as additive terms in the model. For each covariate of the factor type, multiple output files are automatically generated; one for each pairwise comparison between two groups. For each protein, the output files contain an estimate of the corresponding $\log_{2}\;FC$ and a *p* value based on an approximate *t* test where the degrees of freedom are estimated using the Kenward-Rogers approximation.

Hence, msTrawler is restricted to specific designs and does not allow for user-specified contrasts, limiting the research questions that can be inferred from experiments with complex designs.

#### msqrob2TMT

The msqrob2TMT workflows begin with the $\log_{2}$ transformation of the observed PSM intensities. This is followed by normalization of the TMT channels by subtracting the median $\log_{2}$ intensity of each channel to account for loading differences. This normalization step assumes that the majority of proteins are not DA. For our protein-level workflows, PSMs are further summarized into protein abundance values using Tukey’s median polish method, applied separately to the PSM data of each protein within each run. No imputation is performed in the default msqrob2TMT workflows. However, users may define custom preprocessing pipelines by leveraging the functionalities provided in the QFeatures package for filtering, normalization, and imputing missing intensities.

Note that for the msTrawler multibatch benchmarking experiment, our msqrob2TMT workflows start from data that have been custom-filtered and normalized (see “msTrawler Multibatch Benchmarking Experiment”). Subsequently, the reference channels are removed, the data are $\log_{2}$-transformed, and summarized for our protein-level workflows.

msqrob2TMT can fit linear mixed models for each protein, which can be implemented in our msqrob2 package from version 1.14 of the Bioconductor release 3.20 (11), onward. Our PSM-level msqrob2TMT workflows directly models PSM-level data, while our protein-level msqrob2TMT workflows begin with summarized data following median polish summarization.

Our msqrob2TMT framework is very flexible. The model specification has been made fully compatible with the lme4 R package for fitting mixed models. Additionally, it leverages the QFeatures data infrastructure, allowing the incorporation of both feature-level (rowData) and sample-level (colData) variables within the model. This facilitates analyses that infer and adjust for feature-specific and sample-specific covariates, as well as their interactions. Consequently, the current msqrob2TMT implementation can accommodate arbitrarily complex experimental designs, provided they can be specified within the framework of linear mixed models:

$$\begin{array}{c} y_{rcms}=x_{rcm}^{t}\beta +z_{rcms}^{t}u+\epsilon_{rcms}\\ \epsilon_{rcms}∼N\left(0,\sigma^{2}\right)\\ u∼MVN\left(0,G\right) \end{array}$$

with $y_{rcms}$ the $\log_{2}$-transformed and normalized intensity for peptide ion of scan *s* of a protein from the sample in tag *c* of mixture *m* of MS run *r*, $x_{rcm}^{t}$ the covariate pattern, for example, diet, time, condition, dilution, and/or media for the sample in channel c of mixture *m* in run r, β the model parameters modelling the effect of each covariate, and $z_{rcms}^{t}$ the covariate pattern for the random effects to model the hierarchical correlation structure of the data, that is, mixture, run, sample, PSM, and/or additional covariates needed to address clustered and longitudinal study designs, and **u** the vector of random effects that are multivariate normally distributed with mean **0** and variance-covariance matrix **G**, that is, MVN(0,G). Note that the residuals $\epsilon_{rcms}$ given the random effects are again assumed to be i.i.d. normally distributed with mean 0 and variance $\sigma^{2}$.

For models starting at the protein level the index *s* is dropped, and $y_{rcm}$ becomes the $\log_{2}$-transformed, normalized, and summarized protein abundance values.

Similar to MSstatsTMT, variance components are estimated using REML, and the error variance is stabilized by borrowing strength across proteins using the default empirical Bayes step of limma (10). However, msqrob2 introduces two additional advancements: outliers can be addressed using M-estimation with Huber weights, and treatment effects can be regularized through ridge penalization (12). Note that the ridge penalty is estimated by leveraging the connection between ridge regression and mixed models.

Statistical inference with Wald tests can be conducted for any linear combinations of the estimated model parameters ($L^{T}\beta$), providing our users with full flexibility to infer research hypotheses of interest. We direct readers to the supplementary information for detailed vignettes demonstrating the implementation of our msqrob2TMT workflows, their model parameterization, how to define the appropriate contrasts for the log2 FC of interest, and for the interpretation of the output.

### Method Performance

We benchmark our novel workflows against state-of-the-art tools by examining the number of reported proteins that are TP (i.e., UPS proteins in the spike-in study, and yeast proteins in the multibatch benchmark study), and FP (i.e., HeLa proteins in the spike-in study, and mouse proteins in the multibatch benchmark study). Additionally, we construct true positive rate (TPR)-false discovery proportion (FDP) plots. TPR represents the fraction of truly DA proteins reported by the method, calculated as TPR = TP/(TP + false negative), with false negatives (i.e., UPS proteins in the spike-in study, and yeast proteins in the multibatch benchmark study that were not flagged as DA). FDP denotes the proportion of FPs among all proteins flagged as DA, calculated as FDP = FP/(TP + FP). We also highlight the observed FDP at a 5% false discovery rate (FDR) threshold. Since the FDR represent the expected FDP, that is, the average of the FDPs obtained when the spike-in experiment were to be repeated an infinite number of times, an observed FDP that is very far away from 5% is indicative for a workflow that provides poor FDR control.

Furthermore, we assess the $\log_{2}\;FC$ estimates and compare them to the ground truth. For background proteins (HeLa proteins in the spike-in study, and mouse proteins in the multibatch benchmark study) the ground truth $\log_{2}\;FC$ is zero, whereas for the DA proteins (UPS proteins in the spike-in study, and yeast proteins in the multibatch benchmark study) the ground truth corresponds to their spiked $\log_{2}$ ratio.

## Results

We here develop three novel workflows for labeled experiments in our msqrob2 universe:

1. msqrob2_rrilmm (robust ridge linear mixed model): A novel workflow that models the summarized protein-level expression values using a robust linear mixed model. This model accounts for correlation within TMT-plexes via a random effect, regularizes FC estimates across conditions using a ridge penalty, and corrects for outliers via M-estimation;
2. msqrob2_psm_rrilmm (PSM robust ridge linear mixed model): A novel workflow that directly models normalized PSM intensities using a robust linear mixed model with random effects for sample, PSM, and run. It employs a ridge penalty to regularize FC estimates and uses M-estimation to correct for outliers;
3. msqrob2_psm_rrilmm_refit (PSM robust ridge linear mixed model, refit): This workflow is identical to msqrob2_psm_rrilmm, but reduces the model complexity in order to also fit one-hit-wonder proteins, those for which only one PSM per run is observed.

It is worth noting that our msqrob2TMT workflows also allow for incorporating additional random effects to account for technical repeats or other factors arising from longitudinal or clustered experimental designs.

**Fig. 1:**
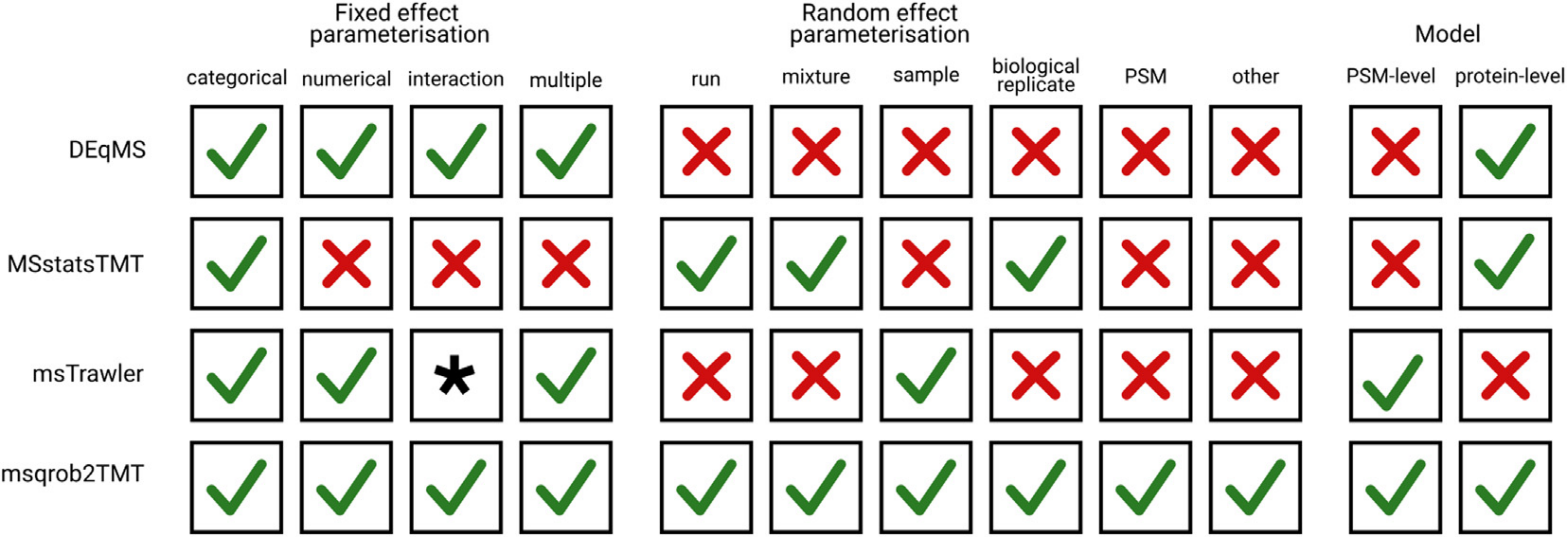
**Overview of the tools considered in this work and the model parameterization they enable**. *Green tick* marks indicate that the method supports the specific model parameterization. Note, that “other” in the random effect parameterization refers to other random effects implied by the design, for example, in studies with longitudinal or clustered designs. (∗) msTrawler can only account for interactions between a factor and a continuous time trend.

We compare our novel msqrob2TMT workflows to the state-of-the-art methods DEqMS (6), MSstatsTMT (Huang *et al*. 2020b), and msTrawler (5). In Figure 1, we provide an overview of the various functionalities of these tools and of our novel workflows. Notably, msqrob2TMT is the only workflow capable of modeling arbitrarily complex experimental designs. DEqMS cannot model correlations in the data using random effects. It can thus only provide valid inference if MS run can be treated as a block effect. Moreover, it cannot handle designs with technical repeats. MSstatsTMT can only infer differential abundance (DA) for designs where the experimental conditions can be specified with a one-way ANOVA layout. It does include random effects for run and mixture. So, it can handle designs where mixtures are run in technical repeats on the MS. msTrawler, on the other hand, models peptide-level data with main effects and a random sample effect to account for correlations between PSMs from the same protein pool. However, it does not account for correlations between samples multiplexed within the same run and can only model interactions between a factor and a continuous time trend. Furthermore, msTrawler is the only tool that does not offer users the functionality to define their own hypotheses for inferring DA. It only provides tests for each slope term in the model or for all pairwise comparisons between the groups of a factor variable.

Using two spike-in studies and one experimental case study, we highlight how the specific implementations of state-of-the-art tools impose limitations on the range of experimental designs for which they can infer DA. We further illustrate how these constraints can be addressed by leveraging the flexibility of our msqrob2TMT workflows and demonstrate its superior performance to prioritize DA.

### MSstatsTMT Spike-In Study

In this study, 40 UPS1 proteins were spiked into a HeLa protein background at four different concentrations. Each spike-in concentration was multiplexed twice within a 10-plex TMT mixture, accompanied by two reference channels. A total of five 10-plex TMT mixtures were prepared, and each mixture was analyzed in technical triplicate on the mass spectrometer. Therefore, the ground truth is known: only the spike-in UPS proteins are DA.

Note that DEqMS cannot account for the hierarchical correlation structure inherent in labeled experiments with multiple runs. While it can correctly infer FCs when all conditions are present in each run by introducing a block factor, it cannot adequately handle technical repeats. Furthermore, it was also unclear how to account for technical repeats from msTrawler’s documentation. Consequently, in the main article, we assess the performance of the different methods using only one technical repeat per mixture. Results for the full study, including the analysis with multiple technical repeats, are provided in the Supplementary Information.

**Fig. 2:**
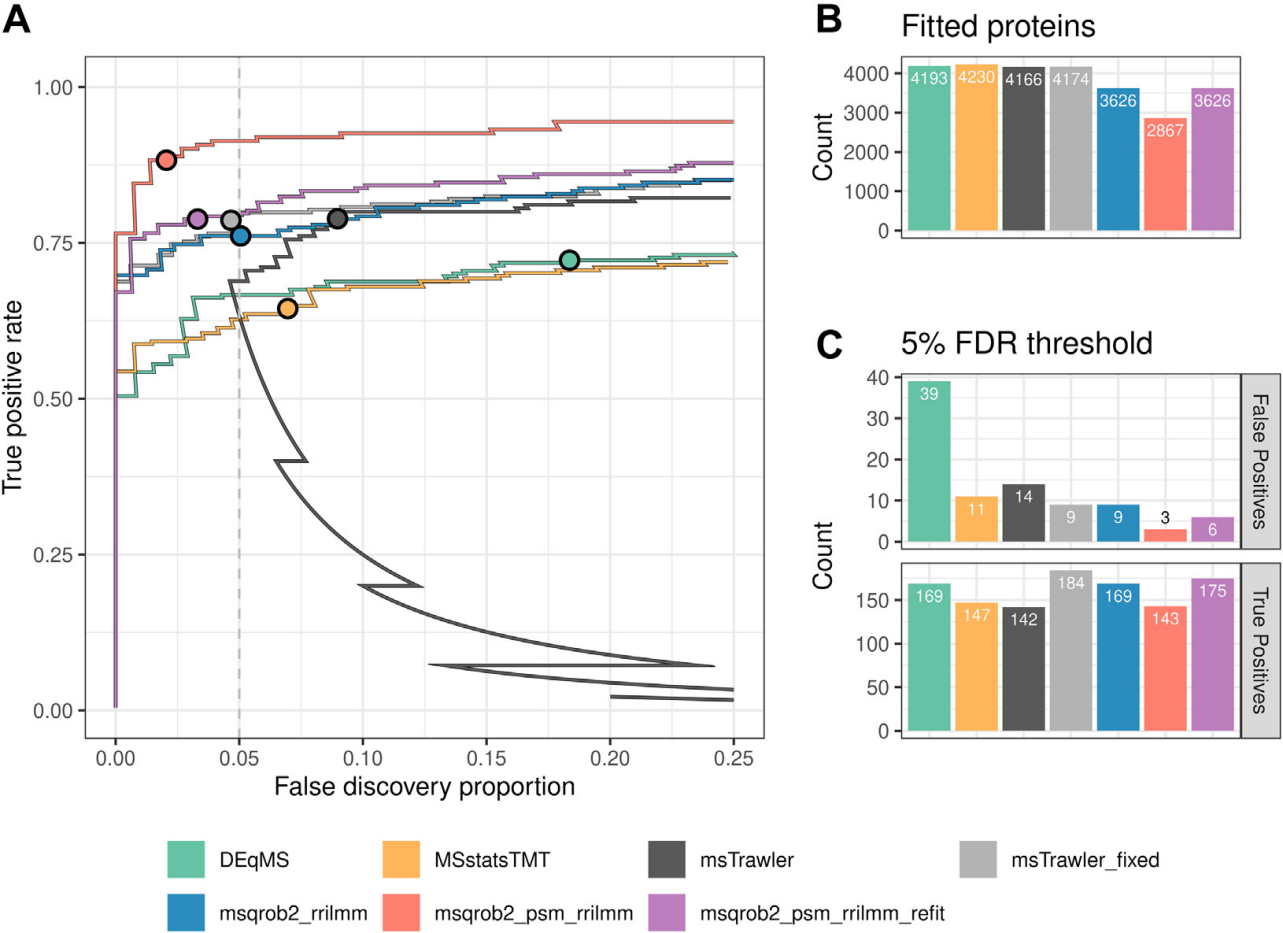
**Performance in the MSstatsTMT spike-in study**. *Panel A*: true positive rate (TPR)-false discovery proportion (FDP) plots for DEqMS, MSstatsTMT, msTrawler, and msqrob2TMT workflows. Note that the import function of msTrawler did not properly import the data. So we also provide results for msTrawler with a refactored import function, referred to as msTrawler fixed. The combined performance over all the dilution comparisons is shown. *Dots* indicate the TPR and FDP obtained at the 5% FDR threshold. *Panel B*: number of fitted proteins for each workflow. *Panel C*: number of true and false positives over all the comparisons. FDR, false discovery rate.

In Figure 2*A*, we compare the performance of the different methods using TPR and FDP curves. The TPR represents the fraction of spiked UPS proteins reported as DA, while the FDP reflects the proportion of HeLa proteins among the total number of DA proteins returned. Our msqrob2TMT workflows clearly outperform the state-of-the-art methods. Indeed, they have a much higher sensitivity (TPR) at the same FDP level.

However, it is important to note that the different methods return results for varying numbers of proteins, as shown in Figure 2*B*. In particular, our novel msqrob2-based workflows tend to report fewer proteins. To ensure a fair comparison, we also present TPR-FDP curves based on all ground truth DA proteins (all 40 spike-in UPS1 proteins) as the maximum number of TP that can be reported for each comparison (Supplemental Fig. S1*B*), as well as plots for the common subset of proteins returned by all methods (Supplemental Fig. S1*C*). Notably, Supplemental Figure S1*B* presents a different picture: here, the PSM-level method msqrob2_psm_rrilmm shows significantly lower sensitivity than the summarization-based approaches. Specifically, msqrob2_psm_rrilmm only returns models for 27 UPS proteins. This is due to msqrob2_psm_rrilmm being unable to fit models for many proteins because of missing data and/or due to one-hit-wonder proteins, that is, those for which only one peptide ion is observed. Indeed, for one-hit-wonder proteins, msqrob2 cannot fit the random sample-level effect and returns an error. To address this, we developed a refit function that can automatically refit PSM-level models for one-hit-wonders in our msqrob2_psm_rrilmm_refit workflow. This adaptation restores top performance when calculating the TPR based on all 40 spike-in UPS proteins that are known to be DA in each comparison. However, it should be noted that msqrob2_psm_rrilmm_refit results in a decrease in sensitivity compared to msqrob2_psm_rrilmm when TPR-FDP curves are based solely on the proteins returned by each method (Fig. 2*A*), indicating that accounting for one-hit-wonders leads to a faster accumulation of FPs among the top-ranked proteins.

Interestingly, msTrawler, msqrob2_psm_rrilmm, and msqrob2_psm_rrilmm_refit show very similar performance when the analysis is based on the common proteins returned by all methods, with msqrob2_rrilmm following closely behind (Supplemental Fig. S1*C*). These results are consistent with findings from label-free proteomics literature, where PSM-level methods typically outperform summarisation-based workflows (e.g., (4)). Remarkably, msTrawler exhibited a performance boost as compared to that in Supplemental Figure S1 panels A and B, which probed us to closely examine its top-ranked FPs. They appeared to be spike-in proteins that were incorrectly labeled, which we could trace back to the msTrawler import function that failed to read-in the data correctly. Upon refactoring the lengthy msTrawler import function, which is referred to as msTrawler_fixed, the performance becomes on par with msqrob2_psm_rrilmm_refit.

These findings are further corroborated by the results at the 5% FDR level, shown in Figure 2*C*. All msqrob2 workflows demonstrate high sensitivity and low FDP. Specifically, they recover between 143 to 175 DA hits (TPs) for spike-in UPS proteins across all six pairwise comparisons while only reporting between 3 to 9 FPs for HeLa proteins. As a result, their FDP ranges between 2.1% and 5.1%, suggesting appropriate FDR control at the 5% level. The default msTrawler workflow, however, was only able to recover 142 TP and reported 14 FP, leading to an FDP of 9%. With our refactored import function, this improved to 184 TP, 9 FP, and an FDP of 4.7%. The summarization-based workflows DEqMS and MSstatsTMT, reported 169 and 147 TP, respectively, with 39 and 11 FP, resulting in FDPs of 18.8% and 7%, respectively, suggesting improper FDR control by DEqMS.

More detailed comparisons are available in Supplemental Figures S2–4, which display TPR-FDP curves for individual pairwise comparisons of the spike-in conditions. These figures highlight that the observed performance improvements of our msqrob2 workflows are particularly pronounced for more challenging comparisons involving low FCs (e.g., spike-in concentrations 0.667–0.5 and 1–0.667). In Supplemental Figure S5, we present results for the full dataset, incorporating all technical repeats. While the results for MSstatsTMT and our novel msqrob2TMT workflows are consistent with those obtained from a single replicate, the difference in performance is somewhat reduced when considering all technical repeats. It is worth noting that only MSstatsTMT and our msqrob2 workflows can appropriately analyze the full dataset, as DEqMS is unable to introduce random effects, and it is unclear in msTrawler’s documentation how to address technical repeats.

**Fig. 3:**
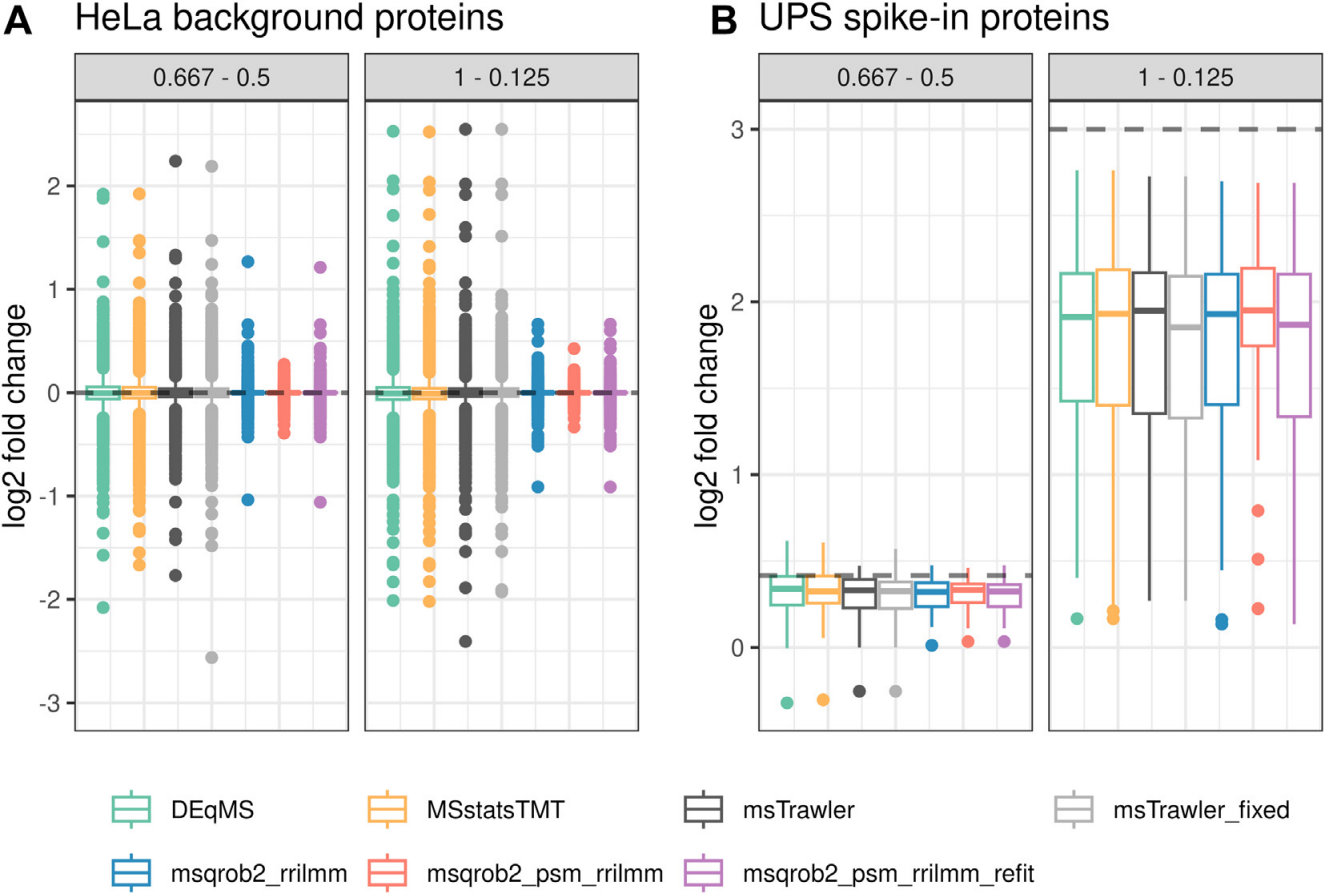
**log2 fold change estimates in the MSstatsTMT spike-in study.** Boxplots showing the log2 FC distributions for the spike-in and nonspike-in proteins, focusing on two comparisons with the highest and lowest difference in spike-in concentrations. Fold changes are estimated by DEqMS, msqrob2TMT, MSstatsTMT, and msTrawler workflows. The *gray dotted line* is the true log2 FC for the comparison. *Panel A*: nonspike-in proteins (HeLa); *panel B*: spike-in proteins (UPS).

In Figure 3 we evaluate the $\log_{2}$ FC estimates. Figure 3*A* shows that the estimated $\log_{2}$ FC for nonspiked proteins is unbiased across all methods. Interestingly, the variability of the estimates for the msqrob2TMT workflows with ridge regularisation is lower. Note that in comparison with 0.667 − 0.5 and 1 − 0.125, there are extreme $\log_{2}$ FC estimates of −4.82 and −8.25, respectively, for the msTrawler workflow that are not shown in the plots. Figure 3*B* demonstrates that the $\log_{2}$ FC for spike-in proteins are generally underestimated by all methods. This is consistent with prior reports in the literature and likely due to interference from cofragmentation of peptide ions, which causes the $\log_{2}$ FC between reporter ions to be underestimated (13, 14). We observe that the underestimation becomes more pronounced as the $\log_{2}$ FC between conditions increases. The variability of the $\log_{2}$ FC estimates for the spiked UPS proteins is similar across all methods. This clearly illustrates the benefit of the ridge regularization in our msqrob2TMT workflows as it affects only the $\log_{2}$ FC estimates for non-DA proteins while leaving those for DA proteins largely unchanged.

Finally, Supplemental Figure S6 shows that MSstatsTMT, msTrawler, and our msqrob2TMT workflows all produce fairly uniform *p* value distributions for nonspike-in proteins. DEqMS, however, shows a slight inflation at low *p* values. The ridge regression *p* values are slightly overconservative with a notable spike at *p* values equal to 1, due to the shrinkage of FCs for nonspike-in proteins.

#### Impact of Robust Ridge Regression, Imputation, and Reference Normalization

**Fig. 4:**
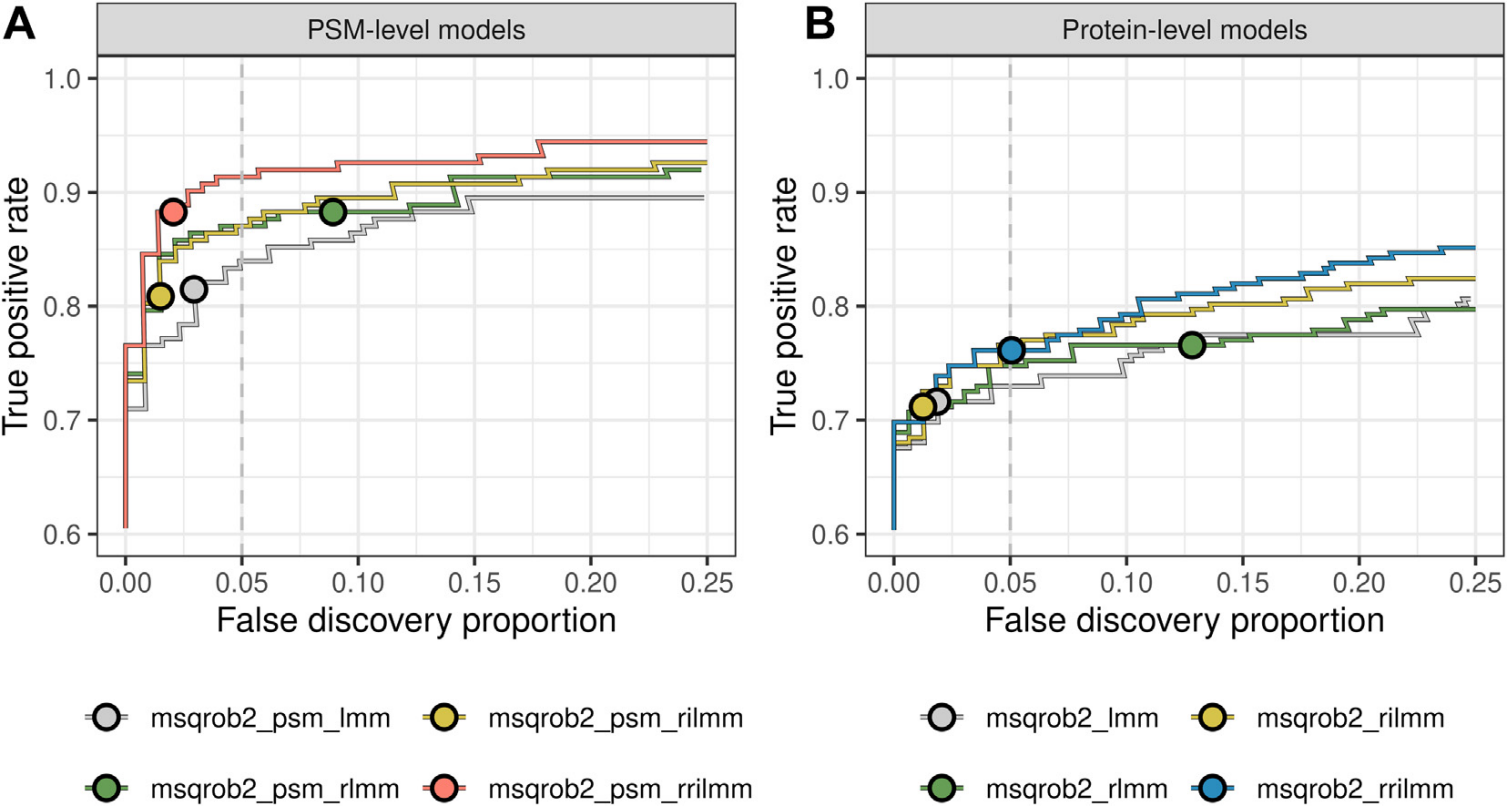
**Performance gain of robust M-estimation and ridge regression.** True positive rate (TPR)-false discovery proportion (FDP) plots showing the effect of robust M-estimation and ridge regression on the performance of the msqrob2TMT protein-level (*panel A*) and PSM-level workflows (*panel B*). Combined performance over all comparisons is shown. Differential abundance is inferred with msqrob2TMT with a vanilla linear mixed model (lmm), a linear mixed model fitted via robust M-estimation (rlmm), ridge regression (rilmm), or robust M-estimation and ridge regression (rrilmm). *Dots* indicate the TPR and FDP obtained at the 5% FDR threshold. FDR, false discovery rate.

A unique feature of the msqrob2TMT workflows is the regularization of parameter estimation through robust M-estimation and ridge penalization. In Figure 4, we evaluate the impact of robust ridge regression on performance within both protein-level and PSM-level models. The results indicate a modest improvement in performance when either robust M-estimation or ridge penalization is applied individually. However, the combination of robust M-estimation and ridge penalization yields the most substantial performance gain.

Note that the msqrob2TMT workflow also differs from MSstatsTMT in its preprocessing methodology. Specifically, MSstatsTMT, by default, imputes missing values and employs reference channels for additional normalization. In Supplemental Figure S7, we evaluate the integration of MSstatsTMT preprocessing with msqrob2TMT model fitting. The findings reveal that imputation and reference channel normalization do not provide additional benefits in the context of this spike-in study. Interestingly, a performance improvement is again observed when robust M-estimation and ridge penalization are applied.

### msTrawler Multibatch Benchmarking Study

The msTrawler multibatch benchmarking study has a more complex design comprising six TMTpro 18-plex batches, where two channels serve as reference channels. One reference channel is derived from a single batch, while the other reference channel includes data from all batches. Yeast proteins cultured on different media were spiked in a background of mouse proteins. The spike-in dilution ratios range from 1, 2, 3, 5, 11, 14, 18, 20, 24, 28, up to 32. Hence, the ground truth is known: only the yeast proteins are DA. A correct data analysis requires modeling of the spike-in condition, while blocking on culture media and accounting for run-to-run variability. Note that this is not possible for MSstatsTMT as users can only specify a single factor to model the variability induced by the experimental design. Consequently, incorporating MSstatsTMT would not provide a fair comparison and has therefore been omitted from the results included in the main text.

It is important to note that the peculiar design of the experiment implies unconventional preprocessing steps. Specifically, there is a much higher number of DA yeast proteins, 1102 proteins, than non-DA mouse proteins, 287 proteins, which violates the typical assumptions of normalization. Hence, we followed the bespoke normalization approach specifically devised for this dataset in the msTrawler paper (5). In particular, the authors based their normalization on mouse proteins with low standard deviation, that is, non-DA proteins that were consistently detected. Note that this approach is not possible with real experimental data, where non-DA proteins are typically unknown. For a fair comparison, we opted to apply this bespoke preprocessing pipeline prior to conducting DA analysis with DEqMS and our novel msqrob2TMT workflows.

**Fig. 5:**
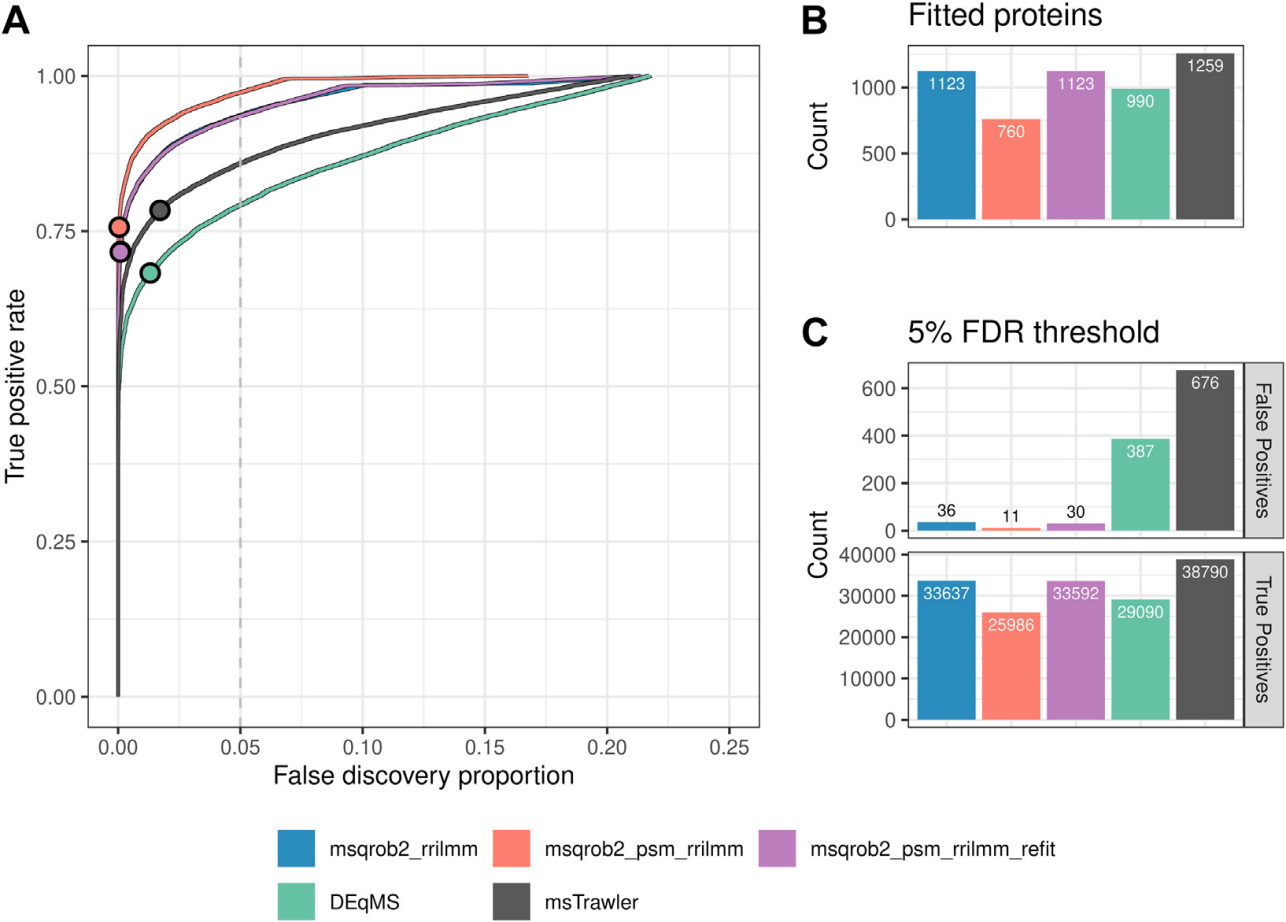
**Performance in the msTrawler multibatch benchmarking study**. *Panel A*: true positive rate (TPR)-false discovery proportion (FDP) plots for DEqMS, msTrawler, and msqrob2TMT workflows. Combined performance over all comparisons is shown. *Dots* indicate the TPR and FDP obtained at the 5% FDR threshold. Note that the performance of msqrob2_rrilmm and msqrob2_psm_rrillm_refit are on par, so their TPR-FDP curves almost coincide. *Panel B*: number of fitted proteins for each workflow. *Panel C*: number of true and false positives over all comparisons. FDR, false discovery rate.

In Figure 5, we compare the performance of our msqrob2TMT workflows with the msTrawler and DEqMS workflows across all dilution comparisons. The TPR-FDP plot in Figure 5*A* demonstrates that msqrob2_psm_rrilmm is again the top performer followed by the msqrob2_rrilmm and msqrob2_psm_rrilmm_refit methods, which are on par and outperform both msTrawler and DEqMS that exhibit lower sensitivity. However, in Figure 5*B*, a significant difference is observed in the number of proteins assessed by each workflow. Notably, msqrob2_psm_rrilmm again reports fewer results due fit errors for proteins with a lot of missing data and one-hit-wonder proteins. Nevertheless, following the refit that allows for one-hit wonders, the number of proteins reported by msqrob2 increases by 47.8%, indicating a very large number of one-hit-wonder proteins. A substantial difference is also observed in the number of FPs returned at the 5% FDR threshold by each workflow (Fig. 5*C*). Specifically, DEqMS and msTrawler report 387 and 676 FPs, respectively, whereas the msqrob2TMT workflows only return between 11 and 36 FPs at the same 5% FDR threshold. Notably, the protein-level msqrob2_rrilmm and msqrob2_psm_rrilmm_refit still report about 4500 TPs more than DEqMS. Lastly, all workflows exhibit an FDP below 5%, which is not unexpected, given that only 20.7% of the proteins assessed are non-DA. Indeed, the default Benjamini–Hochberg FDR method implicitly assumes the fraction of non-DA features to be one so as to provide a conservative estimate of the expected proportion of FPs in the list of significant features it returns (15), which is particularly conservative for this dataset with an artificially high number of DA proteins.

For a fair comparison, we also report TPR-FDP curves using all ground truth DA proteins as the maximum number of TP that can be reported for each comparison (Supplemental Fig. S8*B*) as well as those based on the common subset of proteins assessed by all workflows (Supplemental Fig. S8*C*). In Supplemental Figure S8*B*, we observe a large decrease in performance for PSM-level method msqrob2_psm_rrilmm, which is due to the vast number of one-hit-wonder yeast proteins. However, after refitting, msqrob2_psm_rrilmm_refit recovers performance comparable to that of our msqrob2_rrilmm workflow, which outperforms msTrawler irrespectively of the set of ground truth DA proteins that is used to calculate the TPR (Supplemental Fig. S8, *A*–*C*). Note, however, that the methods also differ in terms of their preprocessing. Specifically, msTrawler employs imputation and additional filtering. In Supplemental Figure S9, we therefore present results for msqrob2TMT models applied on msTrawler’s preprocessed data, which demonstrates a further increased performance. This finding confirms the superior model fitting of our msqrob2TMT workflows compared to msTrawler.

To illustrate the importance of msqrob2TMT’s flexibility in accommodating arbitrarily complex experimental designs, we included results in Supplemental Figure S8 that MSstatsTMT users would obtain if they omitted the blocking effect in their analysis. As expected, excluding this blocking factor leads to a significant reduction in performance.

**Fig. 6:**
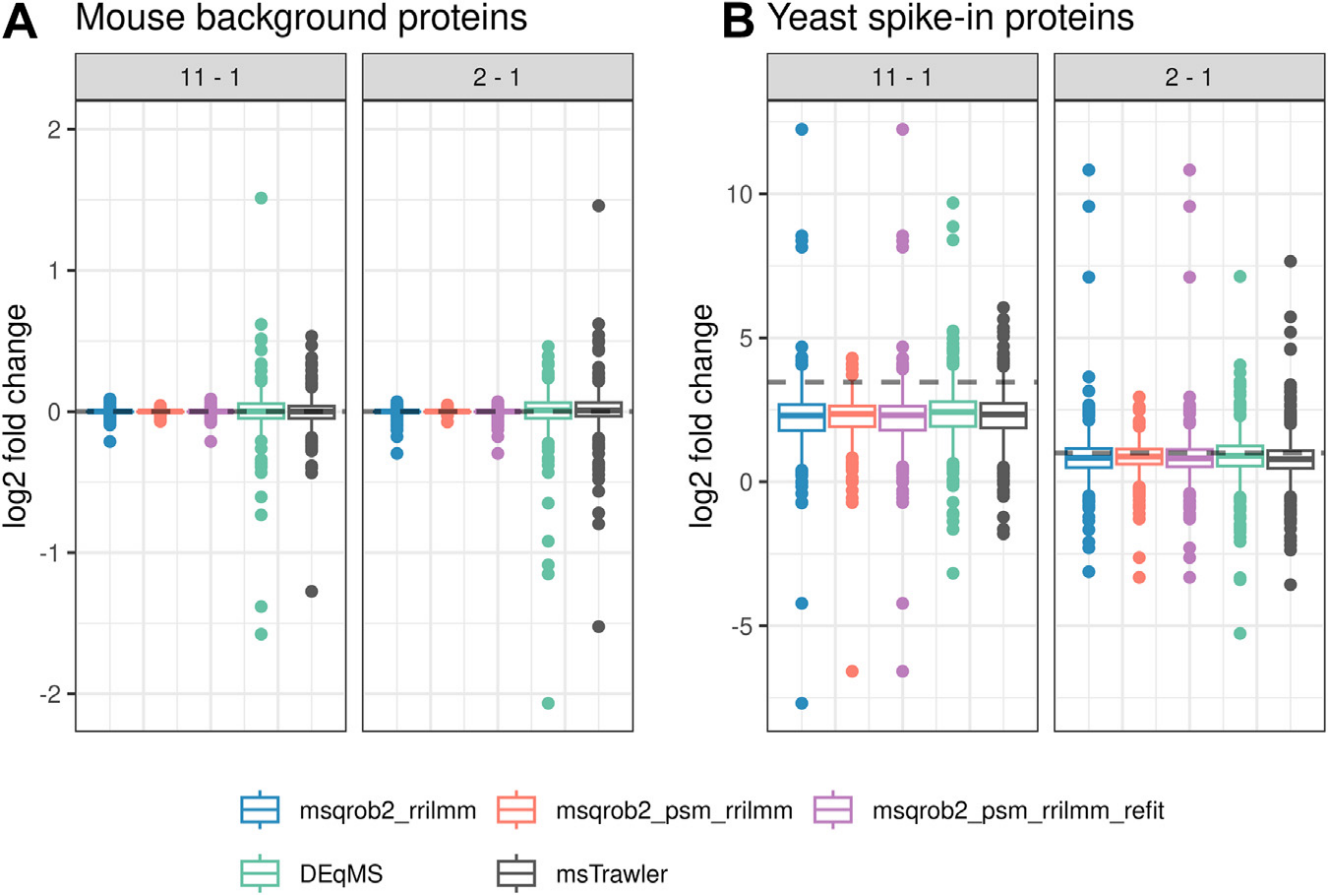
**log2 fold change estimates in the msTrawler multibatch benchmarking study**. Boxplots showing the log2 FC distributions for the spike-in proteins, focusing on two comparisons with the highest and lowest difference in spike-in concentrations. Fold changes are estimated by DEqMS, msqrob2TMT, and msTrawler workflows. The *gray dotted line* is the true log2 FC for the comparison. *Panel A*: nonspiked proteins (mouse); *panel B*: spike-in proteins (yeast).

In Figure 6, we evaluate the $\log_{2}$ FC estimates for non-DA mouse proteins and DA spike-in yeast proteins. Notably, the variability in $\log_{2}$ FC estimates for non-DA mouse proteins is markedly lower and closer to zero for the msqrob2TMT workflows. Similar to Figure 3, $\log_{2}$ FC values for DA proteins are generally underestimated across all workflows, with similar levels of variability observed. This again highlights the beneficial property of our msqrob2TMT robust ridge workflows in reducing variability for non-DA proteins, without affecting these for truly DA proteins.

Finally, Supplemental Figure S10 illustrates that both the msTrawler and msqrob2TMT workflows generate relatively uniform *p* value distributions for nonspike-in proteins. The *p* values derived from ridge regression are slightly overconservative, and due to the shrinkage of FCs for the nonspiked proteins, there is a noticeable peak of *p* values at 1. This histogram exhibits greater variability than Supplemental Figure S6 likely due to the limited number of non-DA mouse proteins in the dataset (287 proteins only).

### Mouse Study

This study investigates the effect of the diet and duration on the proteome of adipocyte cells. The mouse dataset involves mice that were subjected to either an LF or an HF diet for varying durations, also referred to as short or long period. Note that MSstatsTMT cannot accommodate for designs with multiple experimental factors and/or their interactions. We therefore encode the treatment effect as a single factor, with each level corresponding to a specific diet × duration combination. The primary research focus lies on the effect of diet. Accordingly, we prioritize proteins using the following four contrasts:

1. DA between mice exposed to an HF *versus* LF diet after a short period, representing an early diet effect.
2. DA between mice exposed to an HF *versus* LF diet after a long period, representing a late diet effect.
3. DA between mice exposed to an HF *versus* LF averaged over the early and late period, representing an average diet effect.
4. DA between mice exposed to an HF *versus* LF diet differs between long and short period, representing the diet × duration interaction effect.

Note that msTrawler will automatically generate output files for all pairwise comparisons between each diet × duration combination. However, it does not allow users to define their contrasts of interest. Consequently, the diet × duration interaction and the average diet effect cannot be inferred using msTrawler. Furthermore, attempts to utilize msTrawler with multiple reference samples available per run were unsuccessful, even when subsampling was applied. Additionally, we could not address the correlation between fractions with the software, thereby preventing us from addressing the hierarchical correlation structure inherent to the dataset. Due to these limitations, msTrawler was excluded from the analysis.

We also include two protein-level msqrob2TMT workflows. In the first workflow, msqrob2_rrilmm, peptide ions are summarized to the protein-level for each run, resulting in multiple protein abundance values for each biological replicate, that is, one in every technical fraction. In the second workflow, msqrob2_rrilmm_mixture, we use the MSstatsTMT approach and summarize peptide ions to the protein-level over all technical fractions, resulting in a single protein abundance value for every biological replicate.

Table 1 summarizes the number of DA proteins identified as significant at the 5% FDR-adjusted *p* value threshold by the various methods. First, the PSM-level workflows (msqrob2_psm_rrilmm and msqrob2_psm_rrilmm_refit) return more DA proteins than protein-based workflows for three out the four contrasts tested. Note that the fourth contrast is the diet × duration for which few DA proteins were identified, irrespective of the method. Indeed, high-throughput omics studies are known to be under powered for discovering interaction effects (16). Second, modeling the technical MS fraction (msqrob2_psm_rrilmm, msqrob2_psm_rrilmm_refit and msqrob2_rrilmm) leads to a striking increase of the number of DA proteins compared to the workflows that summarize protein intensities over the technical fractions of a mixture (MSstatsTMT, msqrob2_rrilmm_mixture). Third, more DA proteins are found by our msqrob2TMT workflows compared to MSstatsTMT, even when applying the same summarization approach, that is, MSstatsTMT *versus* msqrob2_rrilmm_mixture. Finally, allowing for one-hit-wonder proteins when modeling the data at the PSM level (msqrob2_psm_rrilmm_refit) slightly increases the number of DA, making it the workflow that identifies the most DA proteins. However, as the ground truth for DA proteins in this experimental dataset is not available, we are unable to affirm whether the reported proteins are truly DA.

**Table 1:** Number of differential proteins at 5% FDR for the msqrob2TMT and MSstatsTMT workflows

	Comparison			

Early	Late	Avg	Int

MSstatsTMT	5	9	27	1
msqrob2_rrilmm_mixture	31	6	50	5
msqrob2_rrilmm	80	21	119	7
msqrob2_psm_rrilmm	95	24	132	4
msqrob2_psm_rrilmm_refit	98	29	145	6

The proteins are prioritized using the following four contrasts: DA between HF *versus* LF diet after short period (early), DA between HF *versus* LF diet after long period (long), average DA between HF *versus* LF over the early and late period (avg), and DA of HF *versus* LF diet differs between long period and short period (int).

FDR, false discovery rate; HF, high fat; LF, low fat.

The upset plots (Supplemental Figs. S11–13) demonstrate that the majority of the proteins identified by msqrob2_rrilmm and msqrob2_rrilmm_mixture are also prioritized by the PSM-level models (msqrob2_psm_rrilmm and msqrob2_psm_rrilmm_refit). Additionally, most of the 5, and 27 DA proteins that were reported by MSstatsTMT for the early and average diet contrasts, respectively, were also found significant by other msqrob2TMT workflows. The few proteins uniquely identified as DA by MSstatsTMT where either no longer significant for our msqrob2TMT workflows after correcting for multiple testing, that is, four proteins in the late comparison, or could not be fitted due to missingness, that is, two proteins in the late comparison and all five proteins in the average comparison. Furthermore, proteins uniquely reported by msqrob2_rrilmm_mixture again were no longer significant for the other msqrob2TMT workflows after correcting for multiple testing or could not be fitted due to missingness. Interestingly, the latter proteins exhibited FCs similar to those detected by MSstatsTMT, although their MSstatsTMT *p* values were no longer significant after correcting for multiple testing. Finally, proteins uniquely identified as DA by msqrob2_rrilmm or msqrob2_psm_rrilmm have FCs comparable to those observed in other workflows. Again, the corresponding *p* values in the other methods were no longer significant upon correcting for multiple testing.

We also conducted an overrepresentation analysis for the average diet effect, as this comparison yielded the largest list of DA proteins (Supplemental Fig. S14). The overrepresentation analysis can be performed against a background consisting of the entire mouse proteome or restricted to the proteins identified in the study. The latter approach is more convenient. Indeed, the proteins are isolated from mouse adipose tissue, which is expected to result in an overrepresentation of adipose tissue–specific proteins. Using this restricted background, only seven metabolic pathways were found to be significantly enriched with our msqrob2TMT PSM-level workflows, three of which were also detected by our protein-level workflow msqrob2_rrilmm. However, no Kyoto Encyclopedia of Genes and Genomes (KEGG) pathways were significantly enriched when employing the two protein-level workflows that summarize peptide-ions across fractions (MSstatsTMT and msqrob2_rrilmm_mixture). Hence, we also performed the overrepresentation analysis against the entire mouse background proteome to enable broader comparisons. With this approach, all our novel msqrob2TMT workflows found multiple significantly enriched KEGG pathways, again all related to metabolism, as opposed to MSstatTMT, which did not pick up any enriched KEGG pathway.

Finally, we assessed the validity of the *p* values through a mock analysis. Specifically, we randomly assigned the samples of each diet × duration combination within a mixture to two mock conditions. As none of the proteins exhibit DA between the mock conditions, the corresponding *p* values should follow a uniform distribution. The mock analysis could only be conducted with our msqrob2TMT workflows, as it is not feasible to analyze a two-factor design with additive effects using MSstatsTMT. As shown in Supplemental Figure S15, the *p* value distributions are uniform with a spike on 1. This pattern, also observed in the spike-in study, again originates from shrinkage of the model parameters toward zero. The results of the mock analysis suggest that the additional proteins found to be significant with our novel msqrob2TMT workflows are not driven by overly liberal asymptotic inference.

## Discussion

In this contribution, we introduced msqrob2TMT, a novel suite of workflows within our msqrob2 universe, designed to perform DA analysis for labeled MS-based proteomics experiments. These workflows leverage the flexible robust ridge regression framework of msqrob2 to enhance performance compared to the state-of-the-art tools DEqMS, MSstatsTMT, and msTrawler that uses standard linear (mixed) models.

With the release of msqrob2 version 1.14, we have unlocked full compatibility with the lme4 R package for mixed models, enabling the analysis of data from more complex experimental designs that require the use of both feature-level and sample-level covariates. This advancement allows our workflows to address the intricate hierarchical correlation structure inherent to data from labeled MS experiments, facilitating the analysis of datasets with arbitrarily complex layouts. This capability is particularly critical for modeling data generated in contemporary labeled proteomics experiments, as these utilize ever more complex designs.

Our evaluations on two spike-in studies and a case study demonstrate that none of the three state-of-the-art tools included in our comparisons could provide valid inference across all of these datasets. Notably, MSstatsTMT was unable to account for an additional block effect associated to growth medium in the msTrawler multibatch benchmarking experiment, a limitation with far reaching implications for its applicability in biomedical contexts. Indeed, the inability to adjust for additional covariates and potential confounders undermines the utility of MSstatsTMT in such settings. msTrawler and DEqMS, on the other hand, could not accommodate the more complex correlation structures of the MSstatsTMT spike-in study with technical repeats or the multiple fractions in the mouse case study. Yet these experimental setups are widely employed in the proteomics community, underscoring the limitations of these tools in addressing such commonly encountered experimental designs.

An additional drawback of msTrawler is its restriction on hypothesis testing. Unlike msqrob2TMT, msTrawler automatically generates output files for testing the slope of each additive covariate in the model as well as for all pairwise comparisons between the levels of each factor variable. However, it does not allow users to define custom hypotheses of interest, such as interaction effects between factors or contrasts like the average treatment effect of one factor when they are involved in an interaction. This limitation diminishes its utility in prioritizing proteins of interest, as demonstrated in our mouse case study, where assessing the average treatment effect enabled the identification of the largest number of DA proteins.

Notably, msqrob2TMT not only improves over the state-of-the-art tools in terms of its flexibility but also demonstrates superior performance. In our analysis of the MSstatsTMT spike-in and msTrawler’s multibatch benchmarking datasets, we showed that our novel msqrob2TMT workflows outperform the existing methods in terms of sensitivity, specificity and FDR control. These improvements underscore the importance of the parameter estimation procedure in DA workflows. By extending the linear mixed model to enhance robustness against outliers and improve uncertainty estimation, msqrob2TMT achieves substantial performance gains. The tools also differ in their preprocessing, specifically in normalization and imputation. Different sources of missingness occur, which can lead to suboptimal results when the data are imputed under the assumption of missingness by low abundance. The robust modeling in msqrob2TMT, however, can safely omit imputation altogether. msqrob2TMT is also modular, which provides its users with the flexibility to use custom preprocessing steps, making it future-proof when novel and more performant normalization, summarization, or imputation procedures become available.

Another significant distinction between the msqrob2, MSstatsTMT, and msTrawler packages lies in their approach to model fitting. MSstatsTMT and msTrawler aim to fit as many proteins as possible by automating the model fitting process. While this approach enhances ease of use, it can result in fitting proteins from the same dataset using different models—for instance, combining linear models with mixed models. This practice may not always be appropriate for the analysis and can introduce complexities in interpretation. Although it is possible to retrieve the underlying models and evaluate their validity, standard users may not be fully aware of these issues or the nuanced interpretations they entail. In contrast, the msqrob2TMT workflows prioritize transparency and reproducibility. Rather than automatically reducing models when the user-specified model cannot be fitted, msqrob2TMT explicitly reports a fit error. This approach allows the data analyst to refit specific proteins with a more appropriate model, ensuring greater consistency and interpretability. We developed a refit function for this purpose and provide detailed guidance in our vignettes (Supplementary Information) to support our users in adapting their analysis to incorporate this feature.

Our analyses also highlight the critical need for robust benchmark datasets in the field of proteomics. Both the MSstatsTMT spike-in dataset and the msTrawler multibatch benchmarking study, while valuable, have notable limitations. Specifically, all DA proteins in these datasets are spike-in in the same direction, which puts a severe burden on the conventional assumptions underlying normalization procedures. This can result in issues with channel normalization, as other proteins may become biased downward due to ion suppression effects in samples with high spike-in concentrations. In the msTrawler multibatch benchmarking study, the authors had to develop a bespoke preprocessing workflow tailored specifically to this dataset. Indeed, the data contained up to five times more DA proteins than non-DA proteins and all DA between spike-in conditions is in the same direction, which is highly unrealistic. Notably, their normalization method explicitly had to rely on stably expressed, non-DA proteins, which are typically unknown in a real experimental setting. Conversely, in the MSstatsTMT spike-in study, both non-DA background HeLa UPS proteins as well as spike-in UPS proteins are present. As the HeLa and UPS proteins differ in metabolic labeling, one should be able to discriminate between the non-DA and DA UPS proteins. When closely examining the PSM-level data provided by the MSstatsTMT authors, however, we found spectra that match perfectly with a DA pattern expected for spike-in proteins, while being tagged as HeLa and vice versa. We therefore performed additional preprocessing steps to exclude these ambiguous proteins. Nevertheless, it remains possible that some spectra are still mislabeled. This mislabeling can lead to two key issues: on the one hand, there may be an excess of perceived FPs originating from spike-in proteins that were erroneously tagged as background UPS proteins; on the other hand, there may be a reduction in sensitivity. The inclusion of spectra from mislabeled background UPS proteins diminishes the $\log_{2}\;FC$ estimates and increases the standard errors, which can result in spike-in UPS proteins not being identified as significant. When the analysis was conducted without our additional preprocessing, more FPs were observed. This as a result of background proteins that had not been tagged as spike-in UPS proteins, but clearly exhibited a DA pattern consistent with spike-in UPS proteins. After thoroughly examining these two recent state-of-the-art spike-in studies, we urge the field to progress by developing novel benchmark datasets that encompass more complex DA patterns, where some proteins are upregulated while others are downregulated within each spike-in condition.

Overall, we have demonstrated that our msqrob2TMT workflows offer distinct advantages over state-of-the-art tools. They provide a more sensitive and robust approach, while providing good FDR control. Furthermore, these workflows offer the flexibility to infer DA from data derived from experiments with arbitrarily complex designs, a crucial feature for analyzing large-scale MS-based proteomics datasets that are increasingly prevalent. Our modular implementation gives our users full flexibility with respect to the choice of search engine, preprocessing steps, and the research hypotheses it can address, while delivering a comprehensive, transparent, and reproducible workflow that spans the entire differential proteomics data analysis.

## Data Availability

All analyses presented in this paper can be reproduced using msqrob2 version 1.14.1 from Bioconductor release 3.20, available at https://doi.org/10.18129/B9.bioc.msqrob2, and the scripts on our companion GitHub page: https://github.com/statOmics/msqrob2tmt_paper. We also provide a Docker image containing the computational setup used to generate the results: https://hub.docker.com/repository/docker/cvanderaa/msqrob2tmt. To assist users in building their own msqrob2TMT workflows and interpreting the output, we have included two tutorial vignettes in the Supporting Information. The data from the MSstatsTMT spike-in study, the msTrawler multibatch benchmarking study, and the mouse case study are available in the PRIDE repositories under PXD0015258, PXD036799, and PXD005953, respectively. For the reproducibility of results, the data for PXD0015258, PXD036799, and PXD005953 were downloaded from MassIVE (RMSV000000265), Google Drive (https://console.cloud.google.com/storage/browser/mstrawler_paper), and MassIVE (RMSV000000264), respectively, and timestamped by creating a Zenodo repository: https://zenodo.org/records/14767905.

## Supplemental Data

This article contains supplemental data.

## Conflict of Interests

The authors declare no competing interests.
