# Supplementary material for: msqrob2TMT: Robust Linear Mixed Models for Inferring Differential Abundant Proteins in Labeled Experiments With Arbitrarily Complex Design
Source: Mol Cell Proteomics. 2025 May 30;24(7):101002. doi: 10.1016/j.mcpro.2025.101002 (PMC12365501; doi:10.1016/j.mcpro.2025.101002)
Supplement: Supplemental Data 1 [file mmc1.pdf]

Supplementary material for msqrob2TMT: robust linear mixed models for inferring differential abundant proteins in labelled experiments with arbitrarily complex design

Stijn Vandenbulcke[†,1,2,3] Christophe Vanderaa[†,3] Oliver Crook[4]
Lennart Martens[1,2] Lieven Clement[3,*]

Spike-In Dataset 1

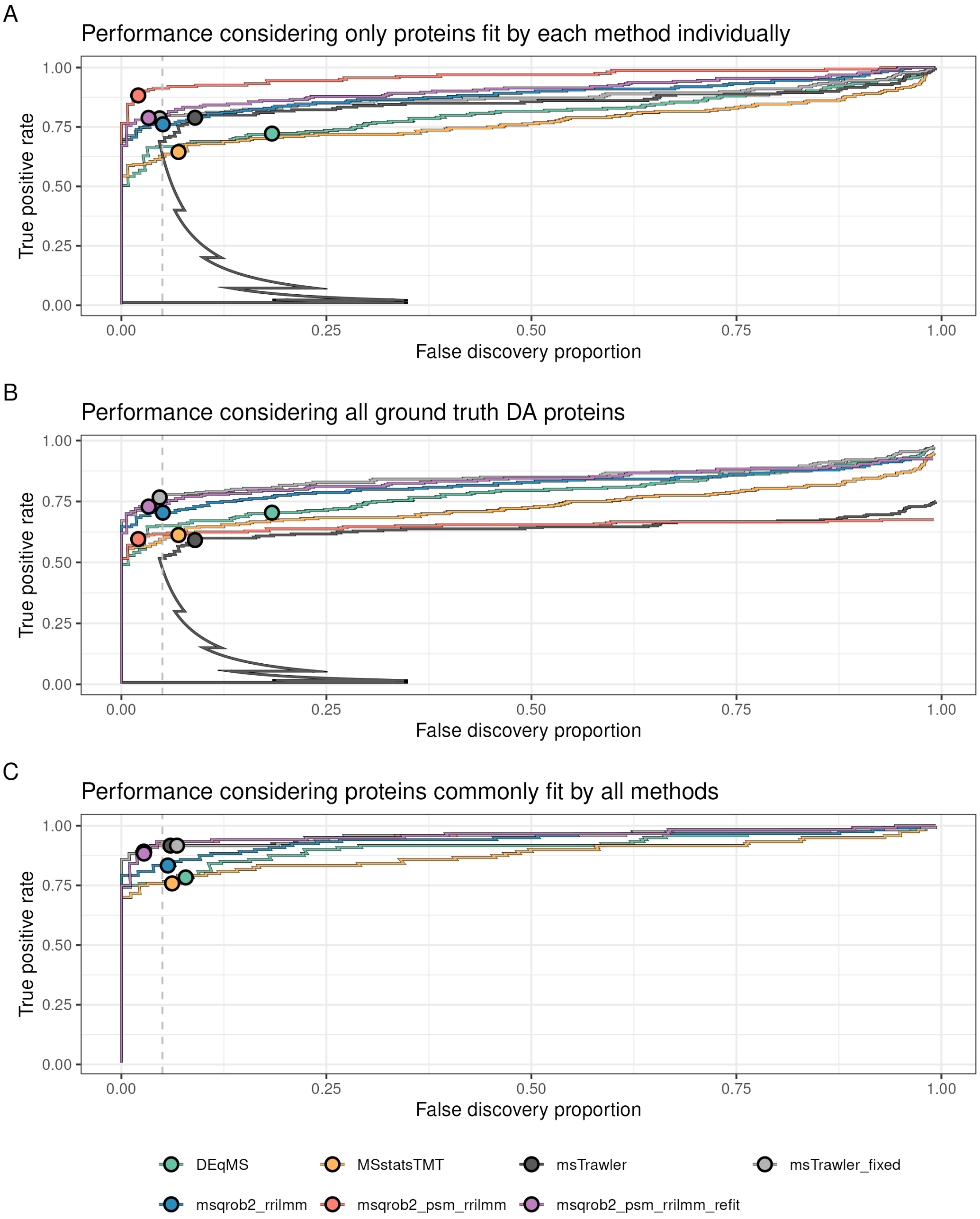

Supplementary Figure 1: True positive rate (TPR) - false discovery proportion (FDP) plots for DEqMS, msqrob2TMT, msTrawler, msTrawler fixed with a refactored import function and MSstatsTMT workflows based on all pairwise spike-in comparisons. Full Caption on the next page.

Supplementary Figure 1: True positive rate (TPR) - false discovery proportion (FDP) plots for DEqMS, msqrob2TMT, msTrawler, msTrawler_fixed with a refactored import function and MSstatsTMT workflows based on all pairwise spike-in comparisons. In Panel A the performance is based on the results that are returned by each workflow, in Panel B using all ground truth DA proteins as the maximum number of true positives that can be reported for each comparison (40 spike-in UPS proteins per comparison), and in panel C by only considering the common proteins that were assessed by every workflow. Dots indicate the TPR and FDP obtained at the 5% FDR threshold.

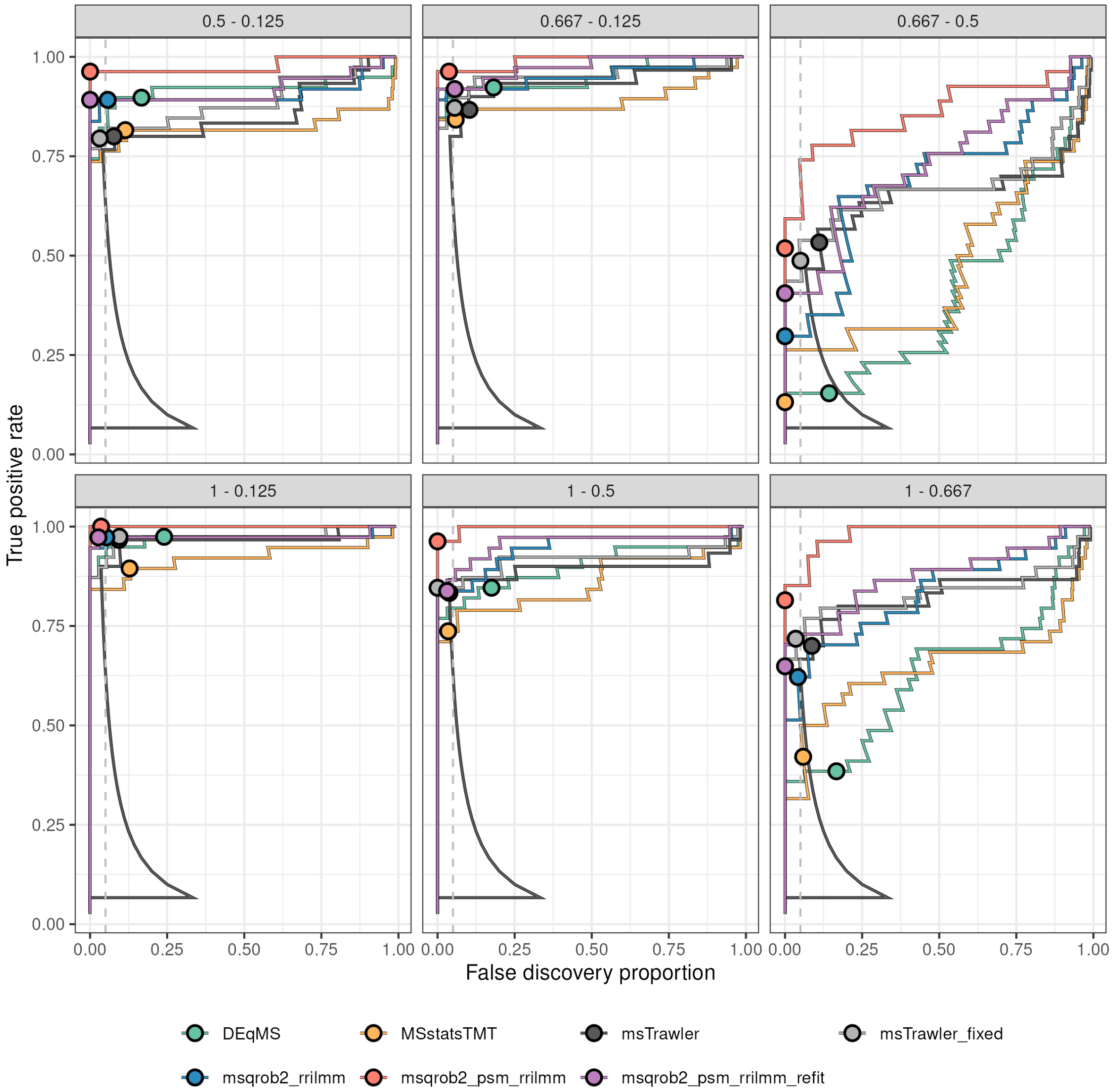

Supplementary Figure 2: True positive rate (TPR) - false discovery proportion (FDP) plots for DEqMS, MSstatsTMT, msTrawler, msTrawler fixed with a refactored import function and msqrob2TMT workflows. The performance is based on the results that are returned by each workflow. Dots indicate the TPR and FDP obtained at the 5% FDR threshold.

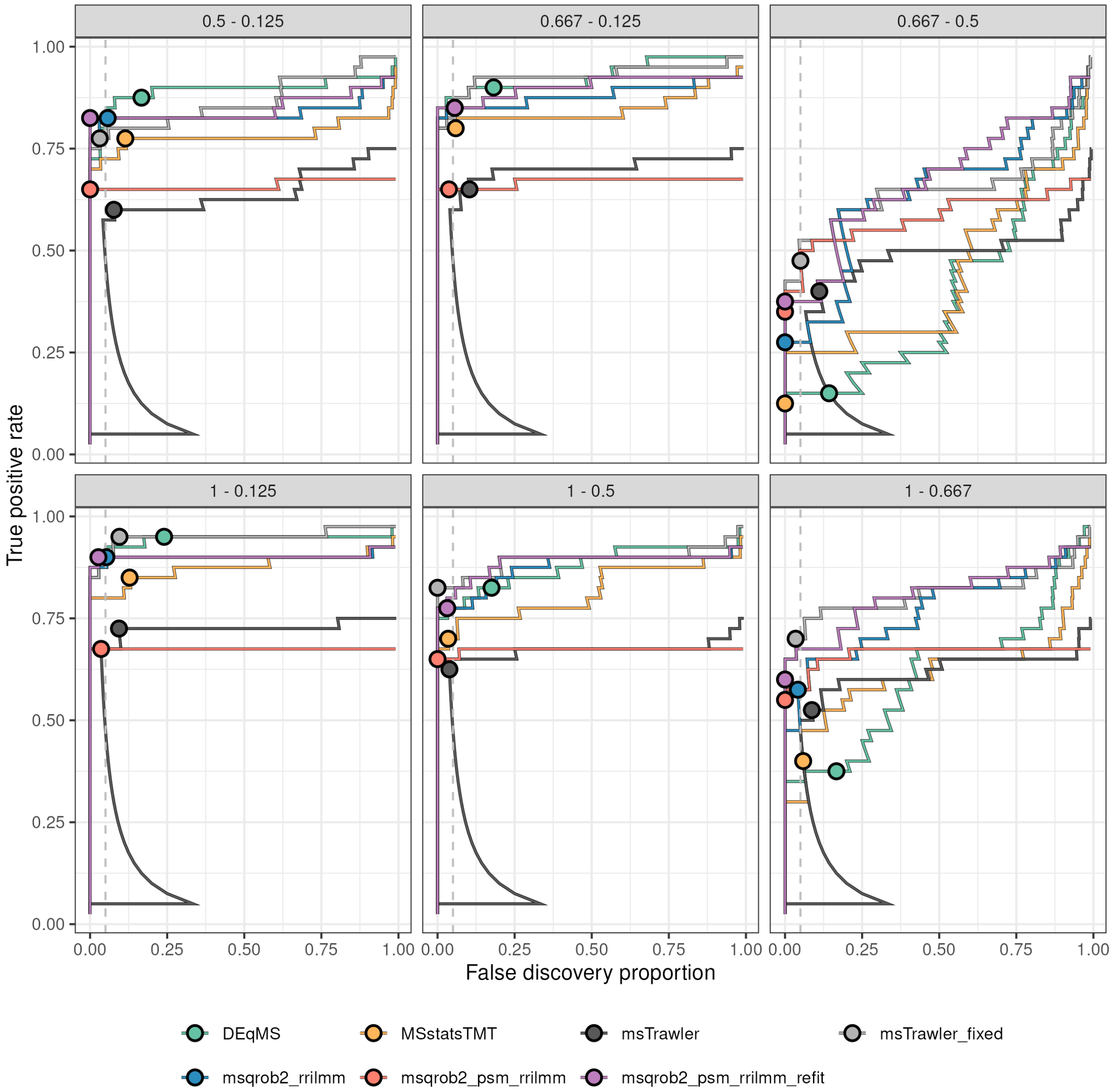

Supplementary Figure 3: True positive rate (TPR) - false discovery proportion (FDP) plots for DEqMS, MSstatsTMT, msTrawler, msTrawler fixed with a refactored import function and msqrob2TMT workflows. The TPR is based on all ground truth DA proteins as the maximum number of true positives that can be reported for each comparison (40 spike-in UPS proteins). Dots indicate the TPR and FDP obtained at the 5% FDR threshold.

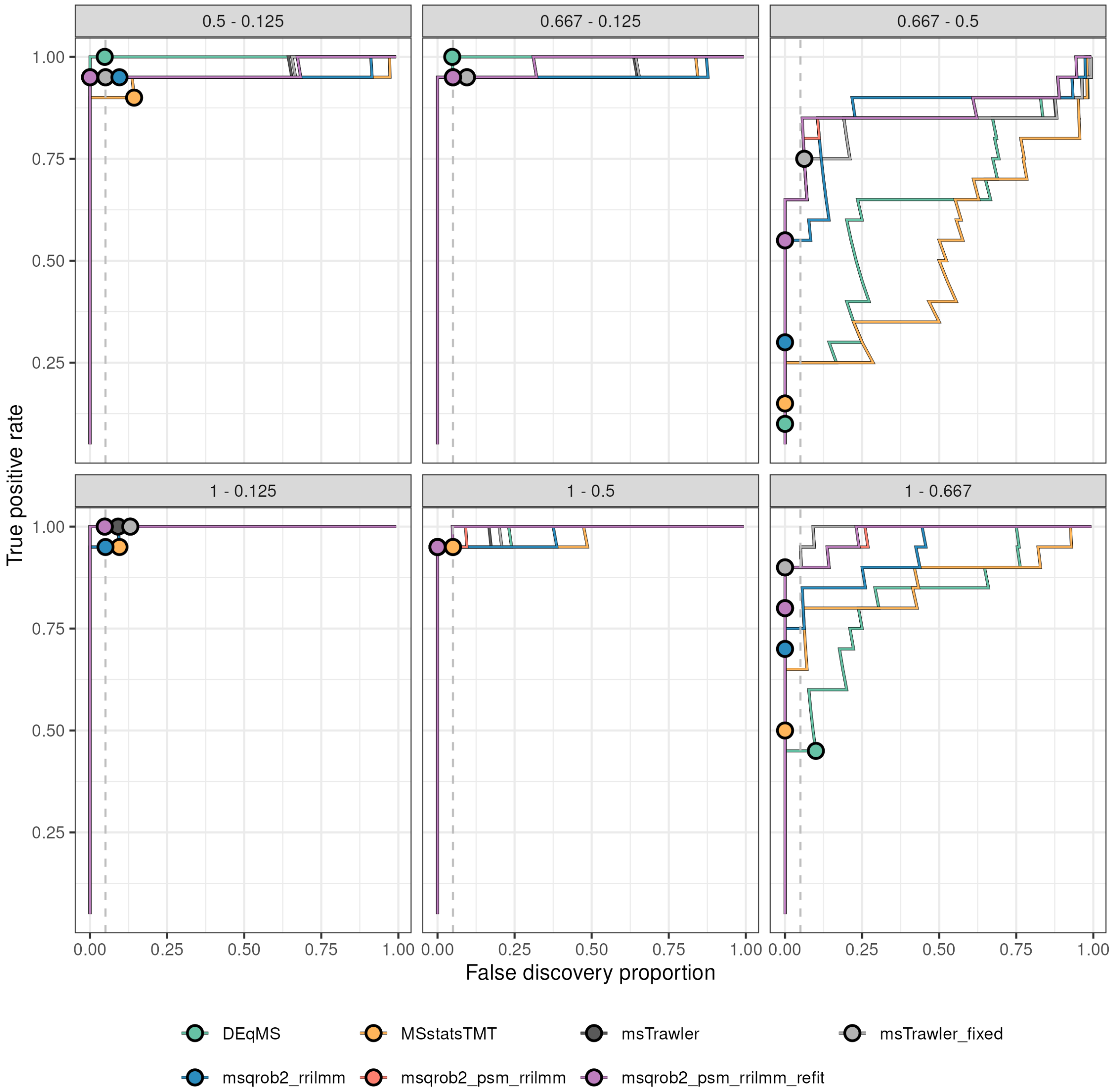

Supplementary Figure 4: True positive rate (TPR) - false discovery proportion (FDP) plots for DEqMS, MSstatsTMT, msTrawler, msTrawler fixed with a refactored import function and msqrob2TMT workflows. The performance is based on the common proteins that were assessed by every workflow. Dots indicate the TPR and FDP obtained at the 5% FDR threshold.

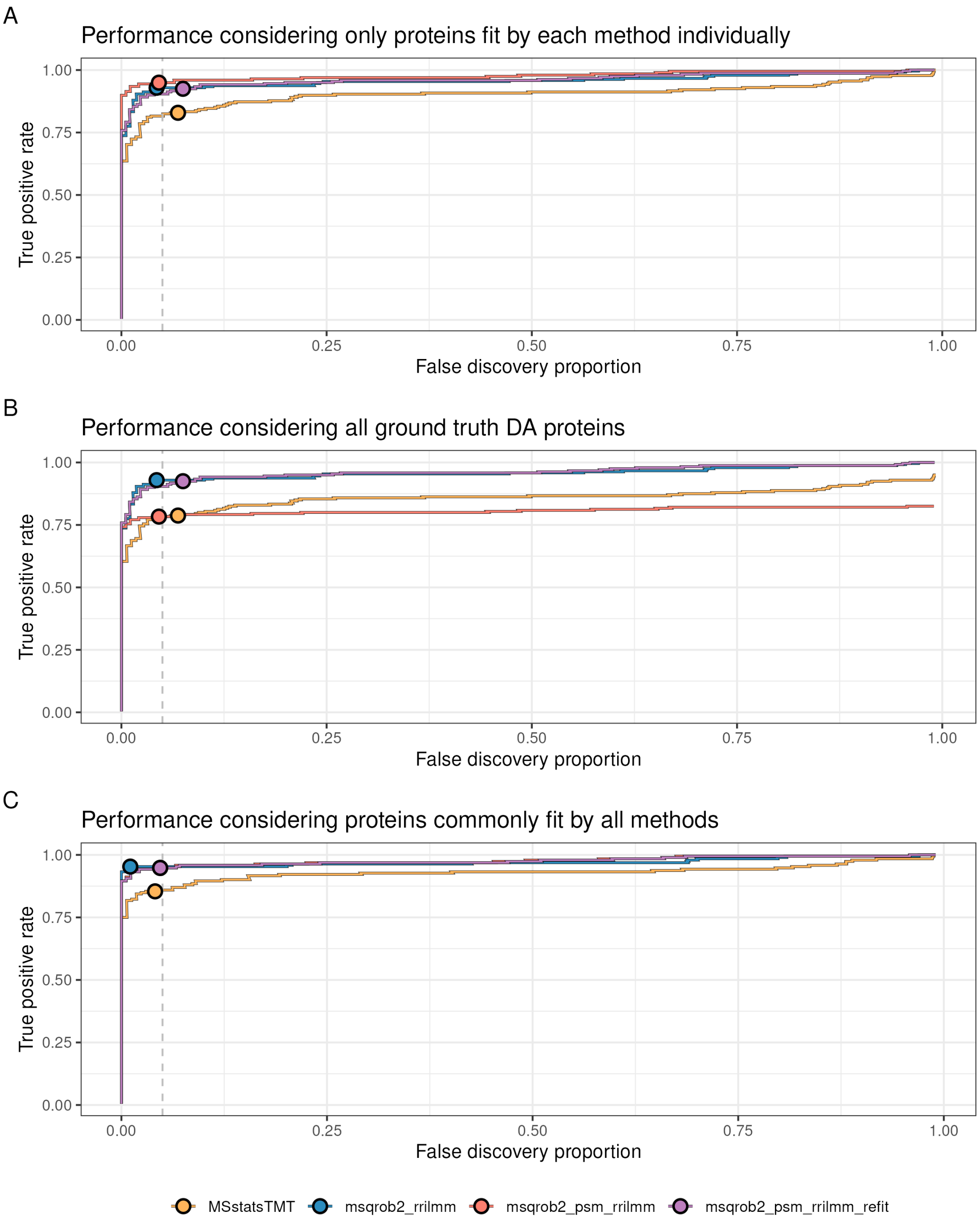

Supplementary Figure 5: True positive rate (TPR) - false discovery proportion (FDP) plots for MSstatsTMT and msqrob2TMT workflows using all technical repeats. The performance is based on all pairwise spike-in comparisons and only considering the results that are returned by each workflow (Panel A), all ground truth DA proteins as the maximum number of true positives that can be reported for each comparison (panel B), and the common proteins that were assessed by every method (panel C). Dots indicate the TPR and FDP obtained at the 5% FDR threshold.

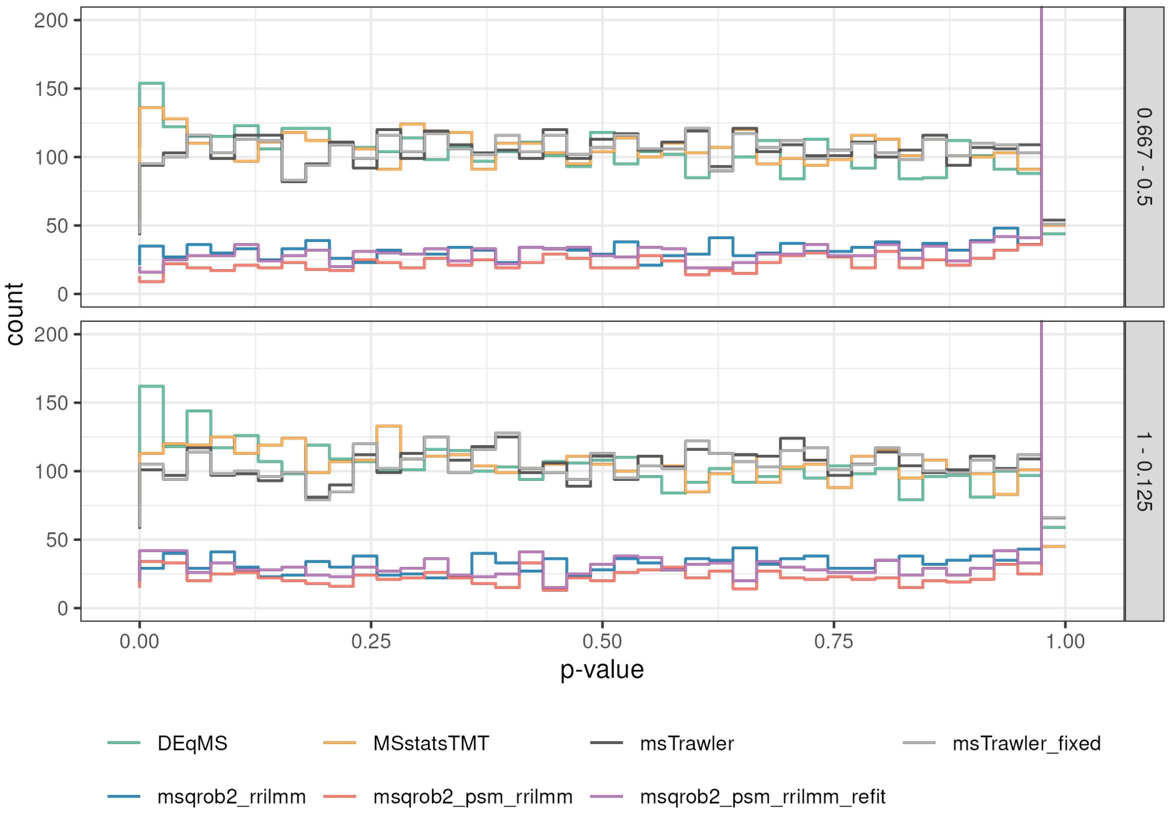

Supplementary Figure 6: Histograms of the p-values from non-spike-in proteins for the DEqMS, msqrob2TMT, MSstatsTMT and msTrawler workflows for two distinct pairwise comparisons between spike-in dilutions.

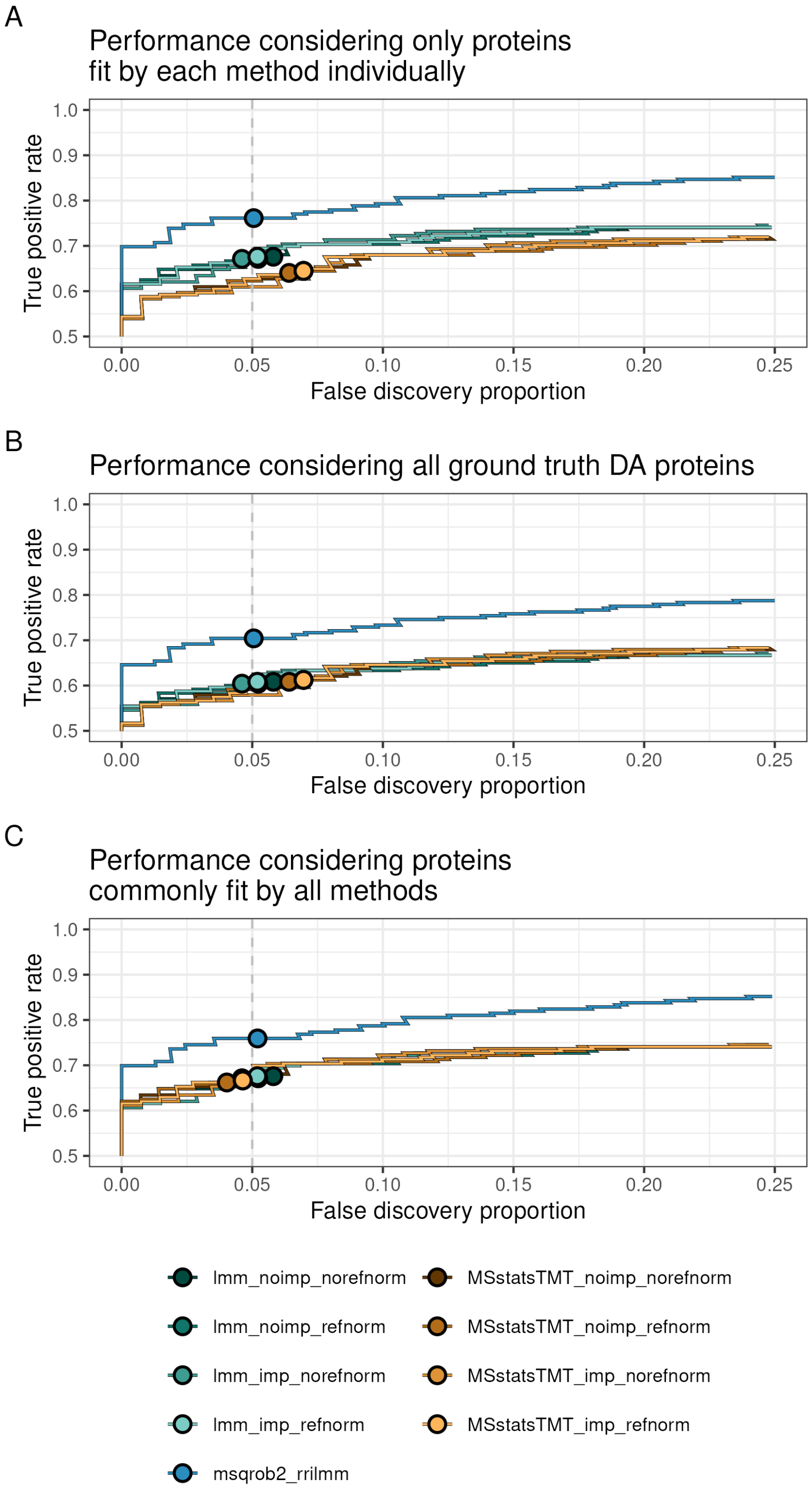

Supplementary Figure 7: Caption on the next page.

Supplementary Figure 7: True positive rate (TPR) - false discovery proportion (FDP) illustrating the effect of the MSstatsTMT preprocessing on the performance for the msqrob2TMT protein-level models. MSstatsTMT preprocessing was conducted both with and without imputation, employing its accelerated failure time model, and, with and without reference normalisation using bridge channels. These configurations are denoted with the suffixes -noimp (no imputation), -imp (with imputation), -norefnorm (no reference normalisation) and -refnorm (with reference normalisation), respectively. Differential abundance on the preprocessed, summarised and normalised protein abundances was subsequently inferred with MSstatsTMT, msqrob2TMT with a vanilla linear mixed model (lmm) or msqrob2TMT employing a linear mixed model fitted via robust M-estimation and ridge regression (rrilmm). The combined performance over all the spike-in comparisons are shown. In Panel A the performance is based on the results that are returned by each workflow, in Panel B on all ground truth DA proteins per comparison (40 spike-in UPS proteins per comparison) and in Panel C by only considering the common proteins that were assessed by every workflow. Dots indicate the TPR and FDP obtained at the 5% FDR threshold.

Multibatch Benchmarking Experiment

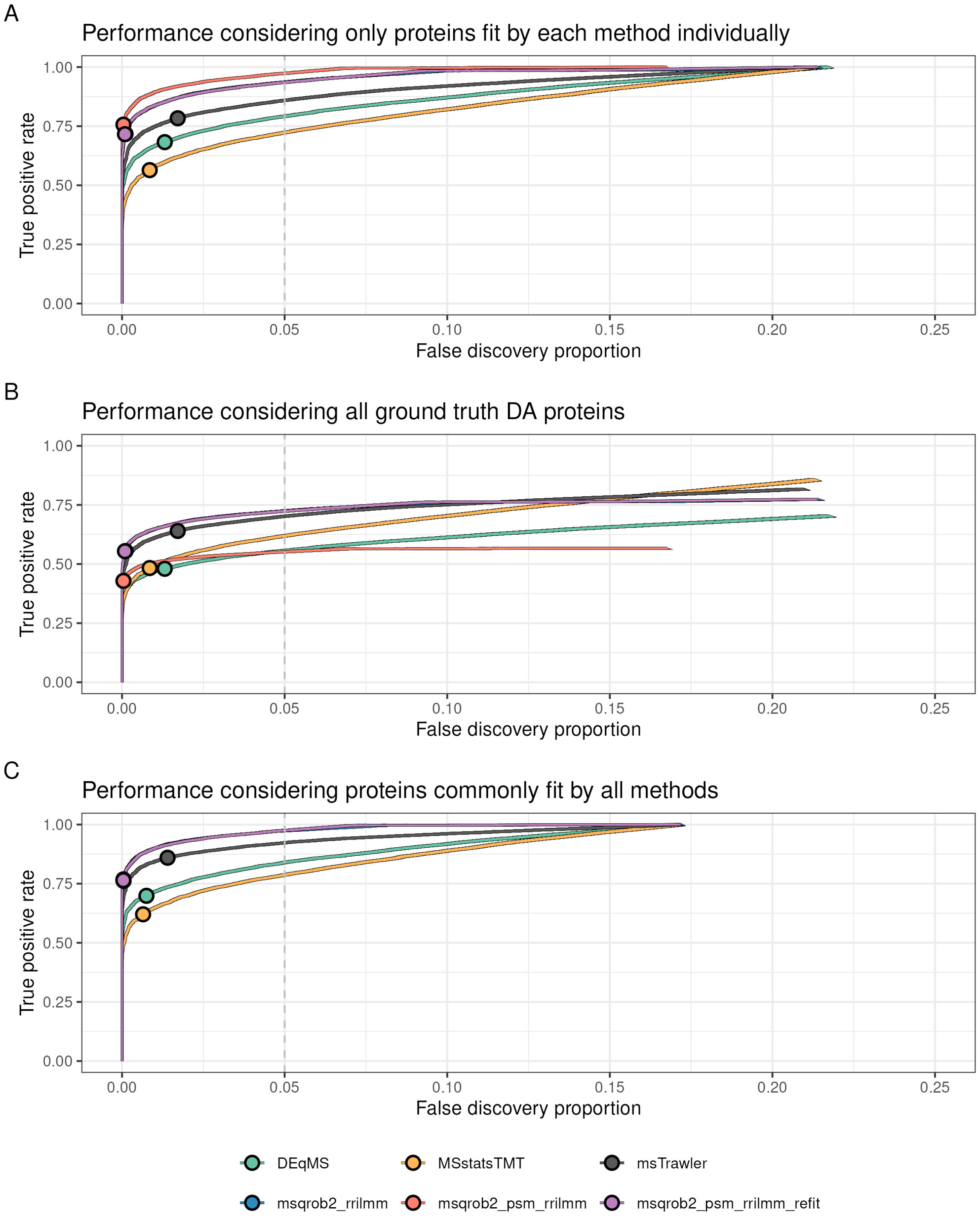

Supplementary Figure 8: Caption on the next page.

Supplementary Figure 8: True positive rate (TPR) - false discovery proportion (FDP) plots for DEqMS, MSstatsTMT, msTrawler and msqrob2TMT workflows. The combined performance over all the comparisons are shown. In Panel A the performance is based on the results that are returned by each workflow, in Panel B on all ground truth DA proteins as the maximum number of true positives that can be reported for each comparison, and in panel C by only considering the common proteins that were assessed by every workflow. Dots indicate the TPR and FDP obtained at the 5% FDR threshold.

Comparing performance after msTrawler preprocessing

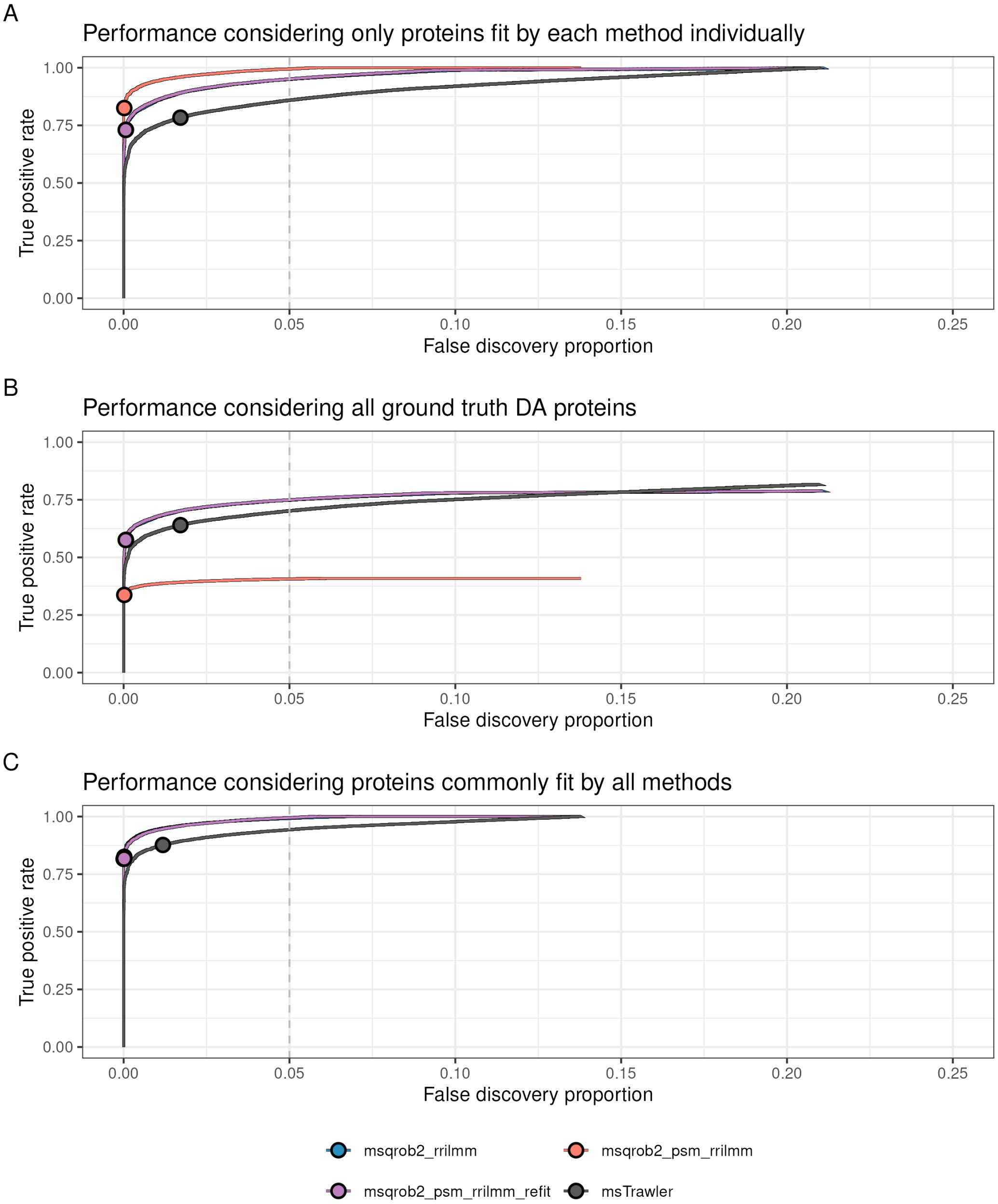

Supplementary Figure 9: True positive rate (TPR) - false discovery proportion (FDP) plots for DEqMS, MSstatsTMT, msTrawler and msqrob2TMT workflows upon full msTrawler preprocessing. The combined performance over all the comparisons are shown. In Panel A the performance is based on the results that are returned by each workflow, in Panel B on all ground truth DA proteins as the maximum number of true positives that can be reported for each comparison, and in panel C by only considering the common proteins that were assessed by every workflow. Dots indicate the TPR and FDP obtained at the 5% FDR threshold.

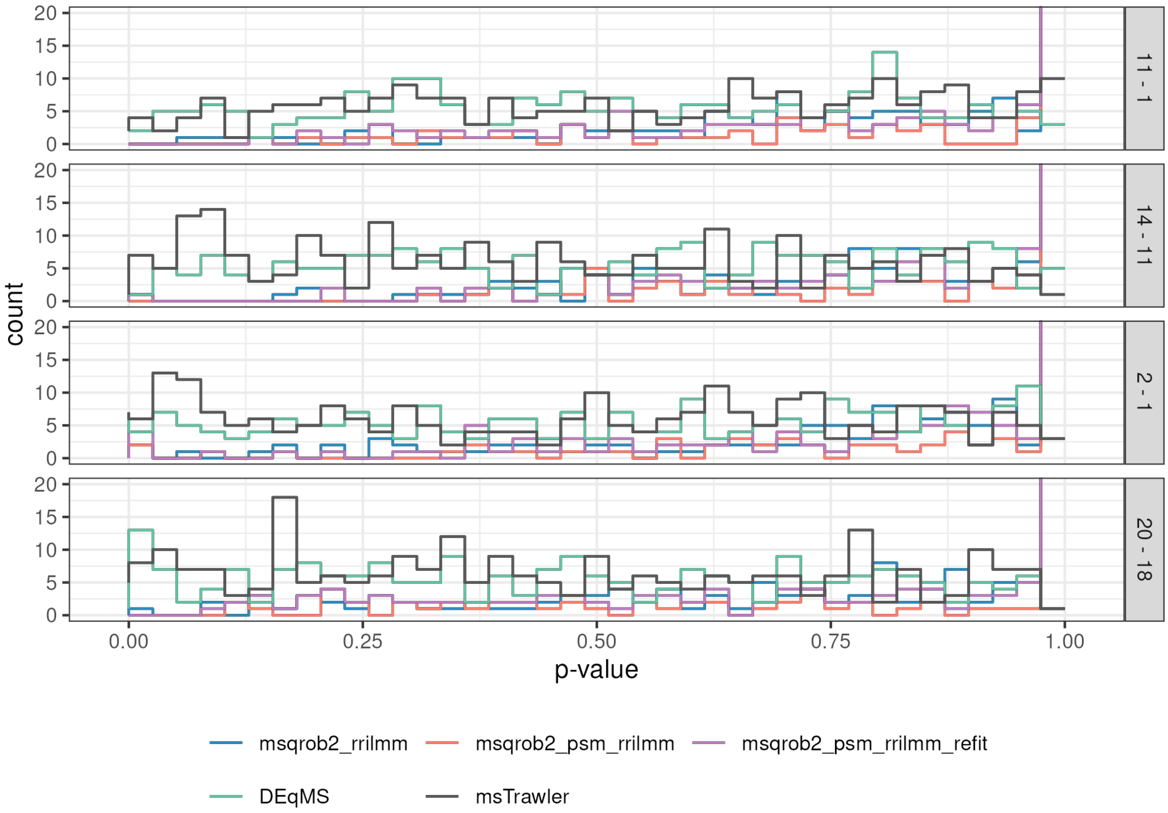

Supplementary Figure 10: Histograms of the p-values from non-spike-in proteins for the DEqMS, msqrob2TMT, and msTrawler workflows for four distinct pairwise comparisons between spike-in dilutions.

Mouse Study

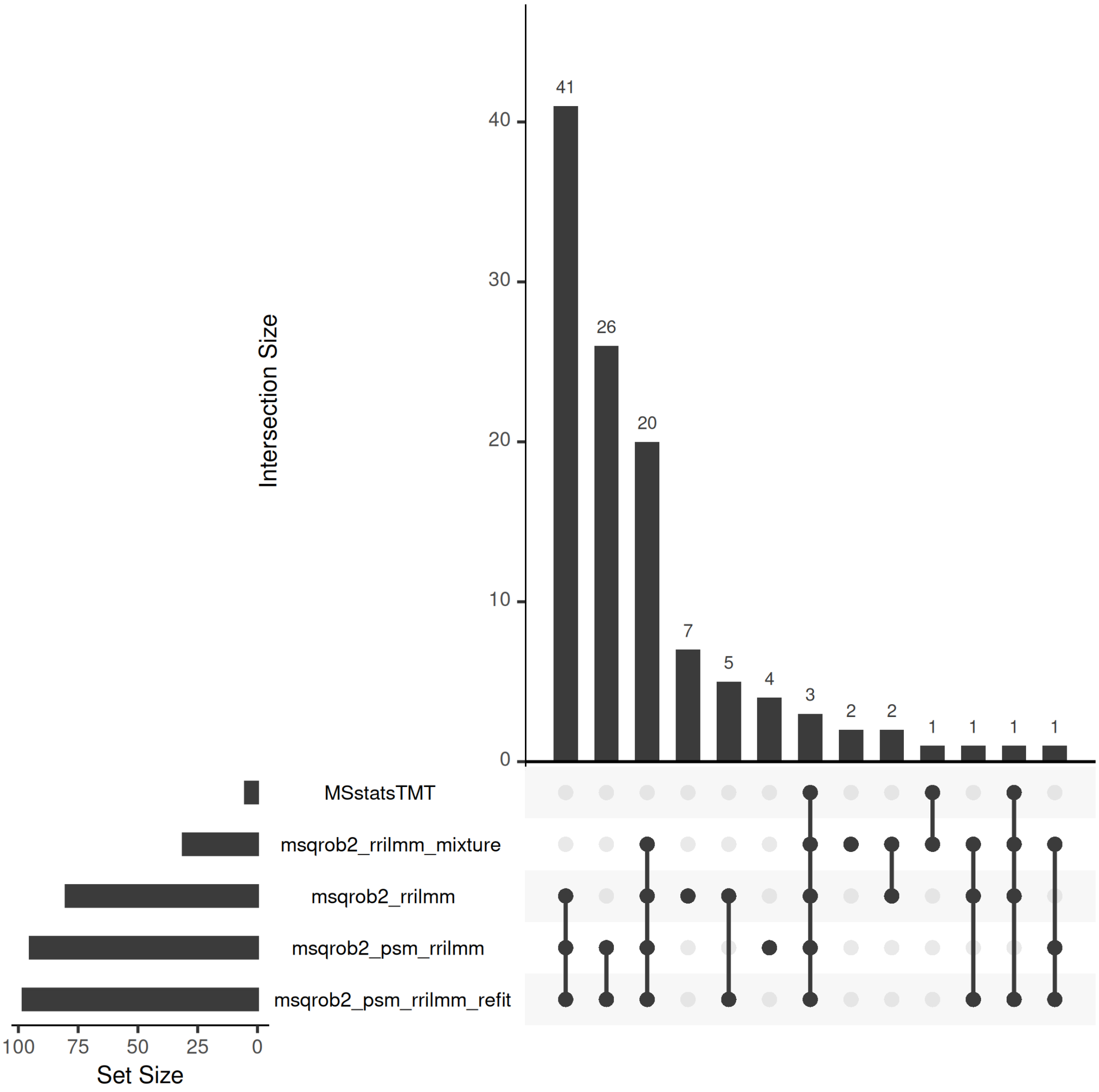

Supplementary Figure 11: Upset plot showing the overlap in significant proteins between the MSstatsTMT and the msqrob2TMT workflows for the early diet effect.

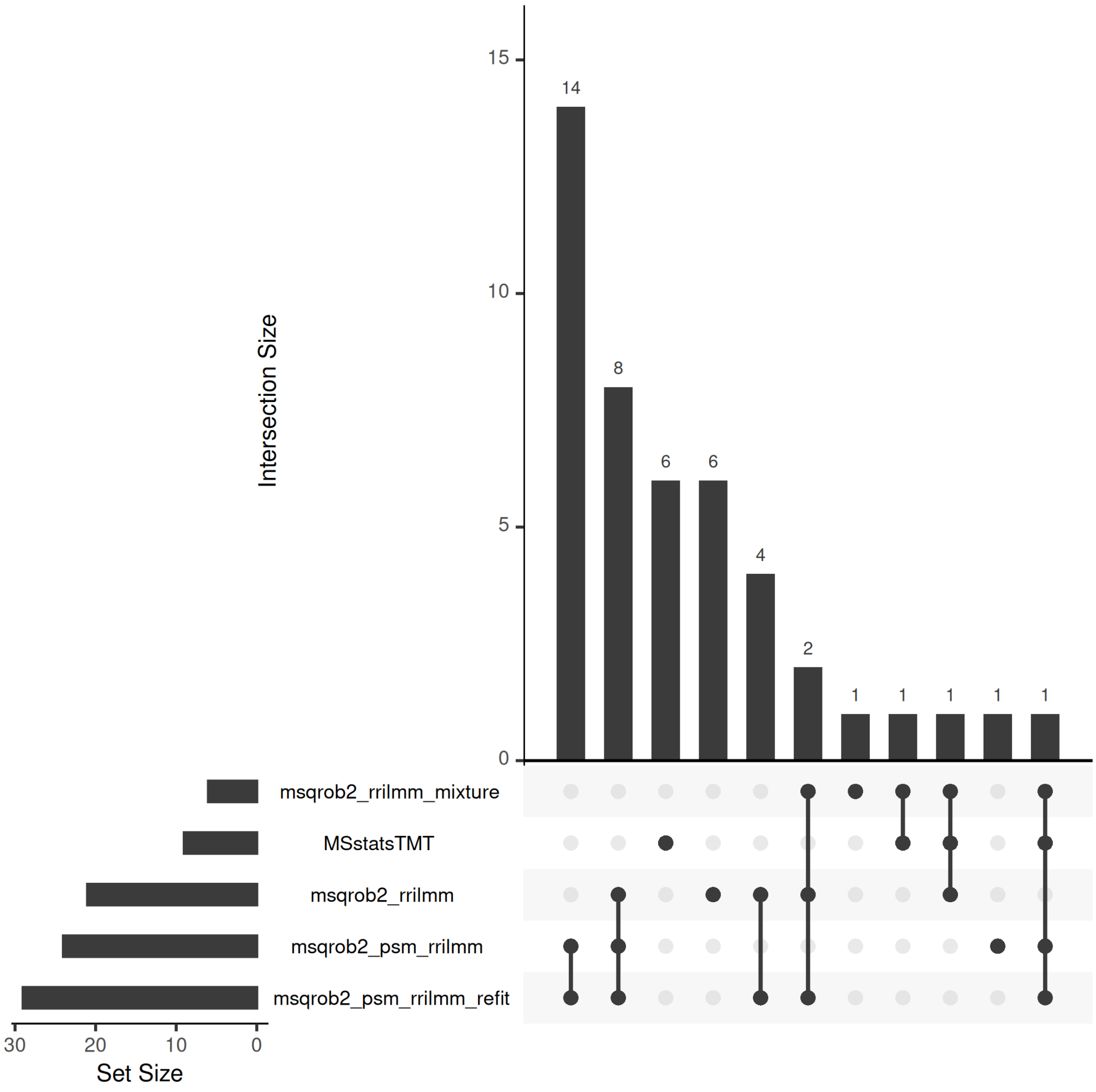

Supplementary Figure 12: Upset plot showing the overlap in significant proteins between the MSstatsTMT and the msqrob2TMT workflows for the late diet effect.

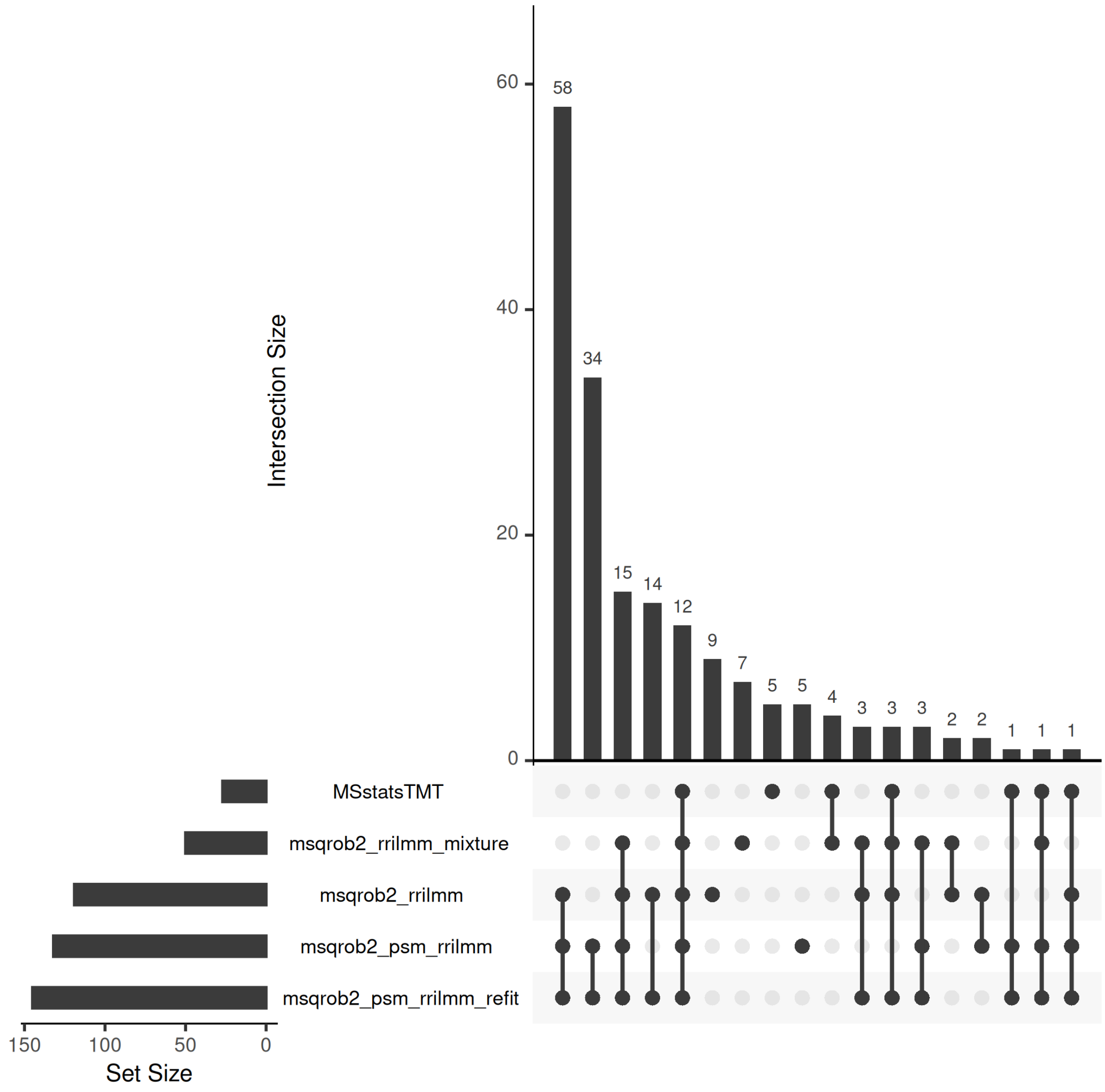

Supplementary Figure 13: Upset plot showing the overlap in significant proteins between the MSstatsTMT and the msqrob2TMT workflows for the average diet effect.

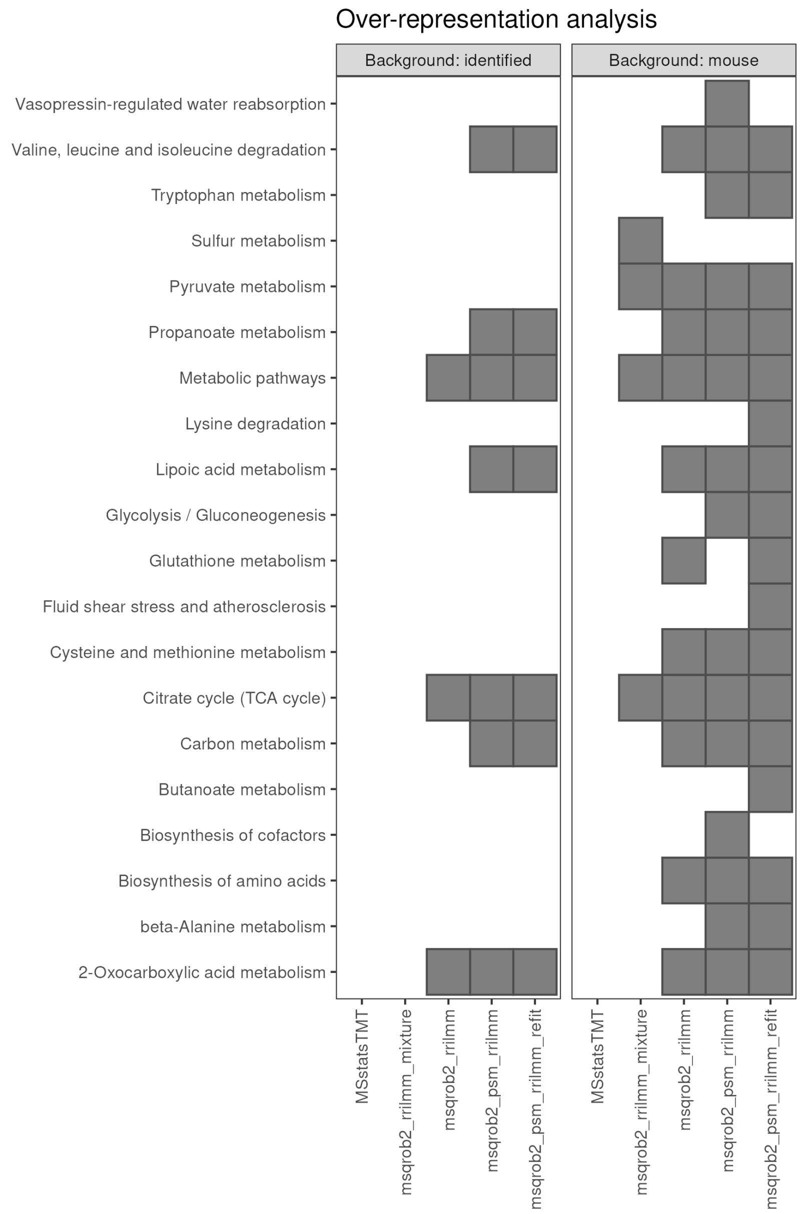

Supplementary Figure 14: Over-representation analysis on the set of proteins identified as significant at a 5% FDR threshold by each method. Annotated protein sets were retrieved from KEGG. The significant proteins were tested for over-representation against either the set of identified proteins in the data set (left) or against the complete mouse proteome. Grey boxes indicate that a KEGG protein set (rows) were identified as over-represented by significant proteins identified by a method (column). The proteins identified by MSstatsTMT did not lead to significant KEGG pathway enrichment, hence the corresponding empty columns.

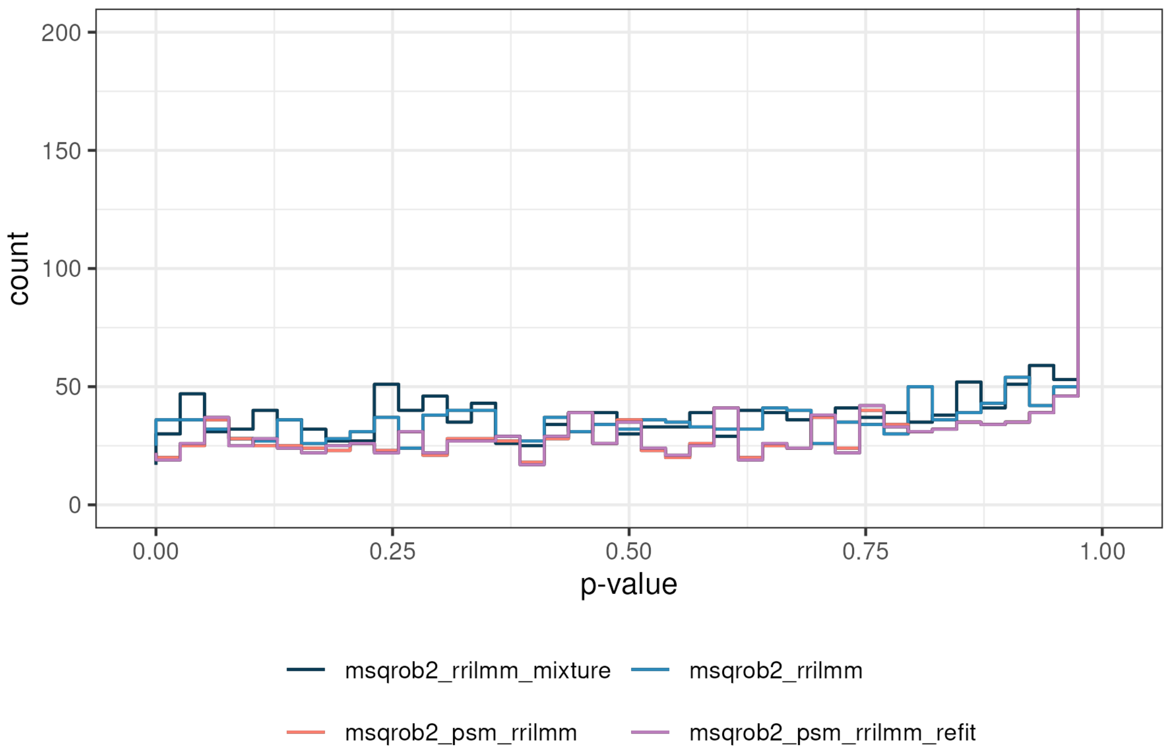

Supplementary Figure 15: Histogram of the p-values from the mock analysis on the mouse dataset. Only results for our PSM-level and protein-level msqrob2TMT workflows are shown, as the state-of-the-art tools could not fit the appropriate models to the data of the mouse study after including an additional mock treatment."
