# Supplementary material for: msqrob2TMT: Robust Linear Mixed Models for Inferring Differential Abundant Proteins in Labeled Experiments With Arbitrarily Complex Design
Source: Mol Cell Proteomics. 2025 May 30;24(7):101002. doi: 10.1016/j.mcpro.2025.101002 (PMC12365501; doi:10.1016/j.mcpro.2025.101002)
Supplement: Supplemental Data 2 [file mmc2.pdf]

Msqrob2TMT use case: the mouse diet use case study

Christophe Vanderaa Stijn Vandenbulcke Lieven Clement

2025-01-31

Table of contents

Introduction **2**

Load packages **2**

Load data **3**

Data preprocessing **4**

PSM filtering 4
Preprocessing workflow 6

Statistical modelling **7**

Model definition 7
One-factor model 10
Two-factor model 23
Protein-level model 27

Analysis at the protein-level by summarising over technical fractions **30**

Read QFeatures object 30
PSM filtering 31
Preprocessing workflow 32
Statistical modelling 33

Conclusion **35**

Citation **36**

License **36**

Session info 36

Introduction

In a previous vignette, we have demonstrated how to model mass spectrometry (MS)-based proteomics data from labelled experiments, ensuring that all sources of variability were correctly accounted for by the model. In this vignette, we demonstrate the application of msqrob2TMT workflows on a real-life data set: the mouse diet case study published by Plubell et al. 2017.

You can read more about `msqrob2TMT` in:

> Vandenbulcke S, Vanderaa C, Crook O, Martens L, Clement L. msqrob2TMT: robust linear mixed models for inferring differential abundant proteins in labelled experiments with arbitrarily complex design. bioRxiv. Published online March 29, 2024:2024.03.29.587218. doi:10.1101/2024.03.29.587218

Before diving into the use case, let us prepare our computational environment.

Load packages

First, we load the `msqrob2` package.

```
library("msqrob2")
```

We also load 4 additional packages for data manipulation and visualisation.

```
library("dplyr")
library("ggplot2")
library("ggrepel")
library("patchwork")
```

`msqrob2` relies on parallelisation to improve computational speed. To ensure this vignette can be run regardless of hardware, we will disable parallelisation. Parallelisation is controlled using the `BiocParallel` package.

```
library("BiocParallel")
register(SerialParam())
```

Load data

The data used in this vignette has been published by Plubell et al. 2017 (PXD005953). The objective of the experiment was to explore the impact of low-fat and high-fat diets on the proteomic content of adipose tissue in mice. It also assesses whether the duration of the diet may impact the results. The authors assigned twenty mice into four groups (5 mice per group) based on their diet, either low-fat (LF) or high-fat (HF), and the duration of the diet, which was classified as short (8 weeks) or long (18 weeks). Samples from the epididymal adipose tissue were extracted from each mice. The samples were then randomly distributed across three TMT 10-plex mixtures for analysis. In each mixture, two reference channels were used, each containing pooled samples that included a range of peptides from all the samples. Not all channels were used, leading to an unbalanced design. Each TMT 10-plex mixture was fractionated into nine parts and subjected to synchronous precursor selection, resulting in a total of 27 MS runs.

The data were reanalyzed by Huang et al. 2020 and have been deposited in the `MSV000084264` MASSiVE repository, but we will retrieve the timestamped data from our Zenodo repository. To facilitate management of the files, we here download them using the `BiocFileCache` package. We need 2 files: the Skyline identification and quantification table generated by the authors and the sample annotation files. `BiocFileCache` ensures that the files are downloaded once, hence the chunk below will take some time only the first time you run it.

```
library("BiocFileCache")
bfc <- BiocFileCache()
psmFile <- bfcrpath(bfc, "https://zenodo.org/records/14767905/files/mouse_psms.txt?download=
annotFile <- bfcrpath(bfc, "https://zenodo.org/records/14767905/files/mouse_annotations.csv?
```

Now the files are downloaded, we can load the two tables. We also perform a little cleanup of the sample annotations to generate the information needed for later data modelling.

```
psms <- read.delim(psmFile)
coldata <- read.csv(annotFile)
coldata$File.Name <- coldata$Run
coldata$Duration <- gsub("_.*", "", coldata$Condition)
coldata$Diet <- gsub(".*_", "", coldata$Condition)
```

We will also subset the data set to reduce computational costs. If you want to run the vignette on the full data set, you can skip this chunk. We here randomly sample 500 proteins from the experiment.

```r
proteinIds <- unique(psms$Protein.Accessions)
set.seed(1234)
psms <- psms[psms$Protein.Accessions %in% sample(proteinIds, 500), ]
```

Finally, we combine the data into a `QFeatures` object. We add to annotation columns so that the `readQFeatures` function can link the PSM to the corresponding quantification column within each run, see `?readQFeatures()` for more details.

```r
coldata$runCol <- coldata$Run
coldata$quantCols <- paste0("Abundance..", coldata$Channel)
mouse <- readQFeatures(psms, colData = coldata,
                       quantCols = unique(coldata$quantCols),
                       runCol = "Spectrum.File", name = "psms")
names(mouse) <- sub("^.*(Mouse.*ACN).*raw", "\\1", names(mouse))
mouse
```

```
An instance of class QFeatures containing 27 assays:
 [1] Mouse_A-J_TMT_40ug_14pctACN: SummarizedExperiment with 198 rows and 10 columns
 [2] Mouse_A-J_TMT_40ug_20pctACN: SummarizedExperiment with 481 rows and 10 columns
 [3] Mouse_A-J_TMT_40ug_22pctACN: SummarizedExperiment with 514 rows and 10 columns
 ...
 [25] Mouse_U-Dd_TMT_40ug_30pctACN: SummarizedExperiment with 726 rows and 10 columns
 [26] Mouse_U-Dd_TMT_40ug_40pctACN: SummarizedExperiment with 566 rows and 10 columns
 [27] Mouse_U-Dd_TMT_40ug_90pctACN: SummarizedExperiment with 251 rows and 10 columns
```

We now have a `QFeatures` object with 27 sets, each containing data associated with an MS run.

Data preprocessing

`msqrob2` relies on the `QFeatures` data structure, meaning that we can directly make use of `QFeatures`' data preprocessing functionality. We will not detail the usage of each function below, but instead refer to the `QFeatures` documentation.

PSM filtering

We remove PSMs that could not be mapped to a protein or that map to multiple proteins (the protein identifier contains multiple identifiers separated by a `;`).

```r
mouse <- filterFeatures(
    mouse, ~ Protein.Accessions != "" & ## Remove failed protein inference
        !grepl(";", Protein.Accessions)) ## Remove protein groups
```

Next,

1. We convert zero intensities to NA and
2. We filter features for which more than 70% of the intensities are missing in a run. We keep the spectrum as soon as the reporter ions are observed in at least 3 out of 10 TMT channels of the run (same cut-off as applied in Huang et al. 2020)/.

```r
mouse <- zeroIsNA(mouse, names(mouse)) ## 1.
mouse <- filterNA(mouse, names(mouse), pNA = 0.7) ## 2.
```

Peptide ions that were identified with multiple PSMs in a run are collapsed to the PSM with the highest summed intensity over the channels, a strategy that is also used by MSstats.

We therefore

1. Make a new variable for ionID in the rowData.
2. We calculate the `rowSums` for each ion.
3. Make a new variable `psmRank` that ranks the PSMs for each ionID based on the summed intensity.
4. We store the new information back in the `rowData`.
5. For each ion that maps to multiple PSMs, only keep the PSM with the highest summed intensity, that is that ranks first.
6. Filter ions for which the rowSum equals 0.

```r
for (i in names(mouse)) {
    rowdata <- rowData(mouse[[i]])
    rowdata$ionID <- paste0(rowdata$Annotated.Sequence, rowdata$Charge) ## 1.
    rowdata$rowSums <- rowSums(assay(mouse[[i]]), na.rm=TRUE) ## 2.
    rowdata <- data.frame(rowdata) |>
        group_by(ionID) |>
        mutate(psmRank = rank(-rowSums)) ## 3.
    rowData(mouse[[i]]) <- DataFrame(rowdata) ## 4.
}
mouse <- filterFeatures(mouse, ~ psmRank == 1) ## 5.
mouse <- filterFeatures(mouse, ~ rowSums > 0) ## 6.
```

Next, we facilitates data integration across different runs by redefining the rownames of each set using on the ion identifier as it is now unique within each run.

```r
for(i in names(mouse)){
    rownames(mouse[[i]]) <- rowData(mouse[[i]])$ionID ## 2.
}
```

Preprocessing workflow

We can now prepare the data for modelling. The workflow consists of mains steps that ensure the data complies to `msqrob2`'s requirements:

1. Intensities are log-transformed.
2. Samples are normalised.
3. (optionally) PSMs intensities are summarised into protein abundance values for protein-level workflows.

```r
sNames <- names(mouse)
mouse <- logTransform( ## 1.
    mouse, sNames, name = paste0(sNames, "_log"), base = 2
)
mouse <- normalize( ## 2.
    mouse, paste0(sNames, "_log"), name = paste0(sNames, "_norm"),
    method = "center.median"
)
mouse <- aggregateFeatures( ## 3.
    mouse, i = paste0(sNames, "_norm"), name = paste0(sNames, "_proteins"),
    fcol = "Protein.Accessions", fun = MsCoreUtils::medianPolish,
    na.rm=TRUE
)
```

We remove the reference channels that were used by the MSstatsTMT authors to obtain normalisation factors since msqrob2TMT workflows do not require normalisation from reference channel. The information about which channels are normalisation channels is available from the `colData`, in the `Condition` column.

```r
table(mouse$Condition)
```

```
 Long_HF  Long_LF   Long_M     Norm Short_HF Short_LF
      45       45       36       54       45       45
```

We remove any sample that is marked as `Norm`. We also remove sample that are annotated as `Long_M` since we could not find documentation for this group.

```r
mouse <- subsetByColData(
    mouse, mouse$Condition != "Norm" & mouse$Condition != "Long_M"
)
```

We conclude the preprocessing by joining the assays of the different runs

1. in a single PSM set for PSM-level models
2. in single protein set for protein-level models

```r
mouse <- joinAssays(mouse, paste0(sNames, "_norm"), "ions_norm") ## 1.
mouse <- joinAssays(mouse, paste0(sNames, "_proteins"), "proteins") ## 2.
```

Statistical modelling

The preprocessed data can now be modelled to answer biologically relevant questions.

Model definition

As described above, samples (adipose tissue) originate from mice that were either subject to a low-fat (`LF`) or high-fat (`HF`) diet. Moreover, each of the diet was maintained for a short duration (`Short`) or a long duration (`Long`). Note that each group contains 5 mice but the peptides from each sample have been fractionated in 9 fractions, leading to 45 units per group.

```r
table(Diet = mouse$Diet, Duration = mouse$Duration)
```

```
    Duration
Diet Long Short
  HF    45    45
  LF    45    45
```

MSstatsTMT (Huang et al. 2020) can only assess ANOVA designs with one factor, so the authors encoded the two-factor design into a one factor design.

```r
table(mouse$Condition)
```

```

 Long_HF  Long_LF Short_HF Short_LF
      45       45       45       45
```

Below, we will show how msqrob2TMT can model these data both using the one-factor and the two-factor encoding. However, we must first identify the potential source of variation in the experiments and include these variables in the model (you can find detailed explanation on the source of variation in another vignette).

1. **Channel effects**: the 20 mouse adipose tissue samples have been labelled using 18-plex TMT. We can expect that samples measured within the same TMT channel may be more similar than samples measured within different TMT channels. Since these effects may not be reproducible from one experiment to another, for instance because each TMT kit may potentially contain different impurity ratios, we can account for this correlation using a random effect for TMT channel. However, we will not model the effects of channels. Normalisation already removes part of the channel effect and including a random effect for channel nested within run (see below) is sufficient, as confirmed from our spike-in studies.

```
length(unique(mouse$Channel))
```

```
[1] 10
```

2. **Mixture effects**: the 20 mouse samples were assigned in one out of 3 mixtures. Again, we expect protein intensities from the same mixture will be more alike than those of different mixtures. Hence, we will add a random effect for mixture.

```
table(mouse$Mixture)
```

```

 PAMI-176_Mouse_A-J  PAMI-176_Mouse_K-T PAMI-194_Mouse_U-Dd
                 54                  63                  63
```

3. **Run effects**: labelled experiments contain multiple samples per run, enabling the estimation of run effect. Indeed, protein intensities that are measured within the same run will be more similar than protein intensities between runs. We will use a random effect for run to explicitly model this correlation in the data. Note that each sample has been acquired in 9 fractions, each fraction being measured in a separate run. Accounting for the effects of run will also absorb the effects of fraction.

```
length(unique(mouse$Run))
```

```
[1] 27
```

4. **Spectrum effects**: we will directly estimate the treatment effect at the protein-level from PSM-level data. This will again induce additional levels of correlation. The intensities for the different reporter ions in a TMT run within the same spectrum (PSM) will be more similar than the intensities between spectra. We therefore need to add a random effect term to account for the within PSM correlation structure. Note that a spectrum here contains the data from one peptide ion within a run. Hence, modelling a random effect for spectrum boils down to modelling a random effect for peptide ion nested within run.

5. **Channel effects nested in run**: modelling the data at the PSM-level also implies that a channel in a run contains multiple PSM intensities for each protein. Hence, intensities from different PSMs for a protein in the channel of a run will be more alike than intensities of different PSMs for the same protein between channels of runs, and we will address this correlation with a random effect for channel nested in run.

6. **Biological replicate effect**: the experiment involves biological replication as the adipose tissue extracts were sampled from 20 mice (5 mice per Diet x Duration combination). We expect that intensities from the same mouse are more alike than intensities between mice. We will therefore also address this correlation with a random effect for biological replicate.

```
length(unique(mouse$BioReplicate))
```

```
[1] 20
```

Now we have identified the sources of variation, we can define a model. We will demonstrate two modelling approaches, depending on how the treatment is encoded:

- The one-factor model (using the `Condition` variable). The variable contains all the level combinations between `Diet` and `Duration`. We also add all sources of technical variation depicted above. Note, that we use an encoding without intercept `~ 0 +`. This makes it more straightforward to define contrasts for a model with a one-way ANOVA design for the fixed effects. Indeed, by suppressing the intercept, a model parameters is estimated for each condition. Otherwise, `msqrob2` selects one of the conditions as the reference group, for which its model parameter is absorbed in the intercept.

```
model1 <- ~ 0 + Condition + ## fixed effect for experimental condition
        # (1 | Channel) + ## (1) random effect for channel is negligible
        (1 | Mixture) + ## (2) random effect for mixture
        (1 | Run) + ## (3) random effect for MS run
        (1 | Run:ionID) + ## (4) random effect for spectrum, i.e. ionID nested in run
        (1 | Run:Channel) + ## (5) random effect for spectrum nested in MS run
        (1 | BioReplicate)  ## (6) random effect for biological replicate (mouse)
```

- The two-factor model (using the Diet and Duration variables). msqrob2 also allows to model using main effects for Diet and Duration, and a Diet:Duration interaction, to account for proteins for which the Diet effect changes according to Duration, and vice versa. Hence, the two-factor model for the fixed effects can be written as Diet + Duration + Diet:Duration, shortened into Diet * Duration. Adding the technical sources of variation, the model becomes.

```
model2 <- ~ Diet * Duration + ## fixed effect for Diet and Duration with interaction
        # (1 | Channel) + ## (1) random effect for channel is negligible
        (1 | Mixture) + ## (2) random effect for mixture
        (1 | Run) + ## (3) random effect for MS run
        (1 | Run:ionID) + ## (4) random effect for spectrum, i.e. ionID nested in run
        (1 | Run:Channel) + ## (5) random effect for spectrum nested in MS run
        (1 | BioReplicate)  ## (6) random effect for biological replicate (mouse)
```

Note, that we do not suppress the intercept here, because the first level of every factor is absorbed in the intercept.

Note also that we have commented out the random effect for channel in both models. In practice, normalisation already removes part of the channel effect and is sufficient. You can experiment this yourself by removing the comment sign # in front of (1 | Channel) + across the vignette, and see how the results may change.

The two model definitions actually describe the same underlying model and there is no theoretical difference between one or the other approach. Notice, however, that the second approach is more flexible as the two factors are not required to be categorical. For instance, another experiment may explore the effect of duration on a numerical time scale, and/or diet may be modelled as the proportion of fat in the diet. Continuous variables cannot be modelled using the first approach.

Now we have defined the models, we can run the msqrob2 statistical analysis workflow.

One-factor model

The statistical workflow starts with msqrobAggregate(). The function takes the QFeatures object, extracts the quantitative values from the "ions_norm" set generated after preprocessing, and fits model1. The variables defined in model1 are automatically retrieved from the colData (i.e. "Condition", "Channel", "Run", "Mixture", "BioReplicate") and from the rowData (i.e. "ionID"). Moreover, we tell the function how the PSM-level data is grouped to protein data through the fcol argument, here we will group PSMs by the Protein.Accessions. The function will generate a new set, proteins_msqrob, with summarised protein values. This new set will also contain the modelling output, stored in the rowData in the msqrob_psm_rrilmm column. More specifically, the modelling output is

stored in the `rowData` for each protein as a `statModel` object, one model per row (protein). We also enable M-estimation (`robust = TRUE`) for improved robustness against outliers and ridge penalisation (`ridge = TRUE`) to stabilise the parameter estimation.

```
mouse <- msqrobAggregate(
    mouse, i = "ions_norm",
    formula = model1,
    fcol = "Protein.Accessions",
    modelColumnName = "msqrob_psm_rrilmm",
    name = "proteins_msqrob",
    ridge = TRUE, robust = TRUE
)
```

Once the models are estimated, we can start answering biological questions.

Difference between low fat and high fat diet after short duration

A first question one can ask is: how are protein abundance affected by diet when only considering a short diet duration? We need to convert this question in a combination of the model parameters, also referred to as a contrast. To aid defining contrasts, we will visualise the experimental design using the `ExploreModelMatrix` package. Since we are not interested in technical effects, we will only focus on the variable of interest, here `Condition`.

```
library("ExploreModelMatrix")
vd <- VisualizeDesign(
    sampleData =  colData(mouse),
    designFormula = ~ 0 + Condition,
    textSizeFitted = 4
)
vd$plotlist
```

```
[[1]]
```

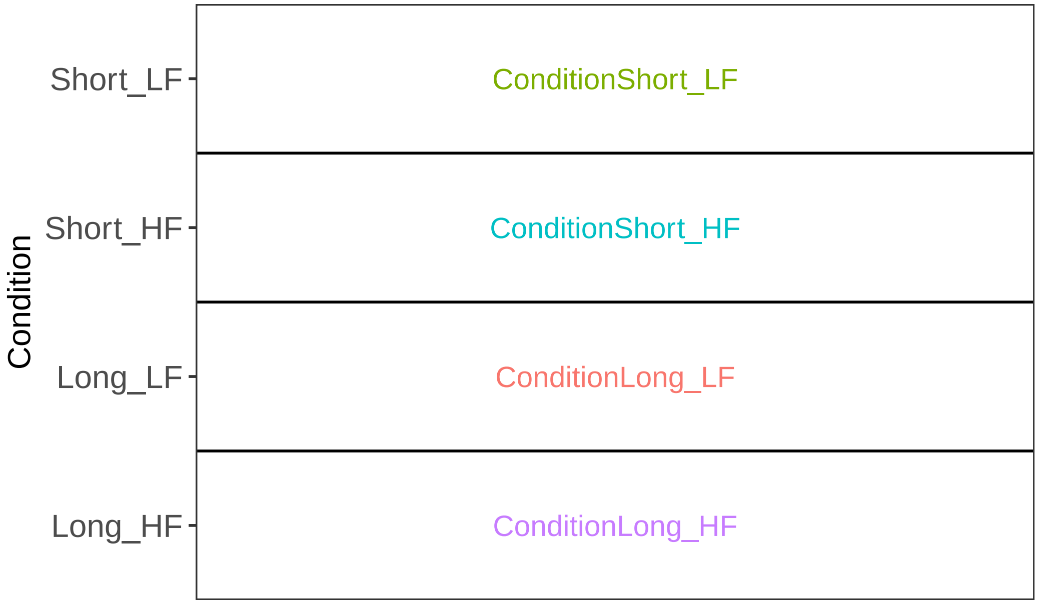

With a single factor, defining contrast is straightforward. So, assessing the difference between low-fat and high-fat diets for short duration boils down to assessing the difference between `Short_LF` and `Short_HF`, hence the contrast `ConditionShort_LF - ConditionShort_HF`. Note that because we used ridge regression for modelling, we need to prefix the parameter names with `ridge`.

```
contrast <- "ridgeConditionShort_LF - ridgeConditionShort_HF"
```

We can further specify the null hypothesis, that is that the differences between the two groups is zero.

```
(hypothesis1 <- paste(contrast, "= 0"))
```

```
[1] "ridgeConditionShort_LF - ridgeConditionShort_HF = 0"
```

We next use `makeContrast()` to build a contrast matrix.

```
(L <- makeContrast(
    hypothesis1,
    parameterNames = c("ridgeConditionShort_HF","ridgeConditionShort_LF")
))
```

```
                         ridgeConditionShort_LF - ridgeConditionShort_HF
ridgeConditionShort_HF                                                -1
ridgeConditionShort_LF                                                 1
```

We can now test our null hypothesis using `hypothesisTest()` which takes the `QFeatures` object with the fitted model and the contrast matrix we just built. Again, the results are stored in the set containing the model, here `proteins_msqrob`

```
mouse <- hypothesisTest(
    mouse, i = "proteins_msqrob", L, modelColumn = "msqrob_psm_rrilmm"
)
```

Let us retrieve the result table from the `rowData`. Note that the model column is named after the column name of the contrast matrix `L`.

```
inference <- rowData(mouse[["proteins_msqrob"]])[[colnames(L)]]
inference$Protein <- rownames(inference)
head(inference)
```

```
              logFC           se      df            t      pval adjPval Protein
A2AJB7 6.958308e-09 0.0001334067 68.0702 5.215863e-05 0.9999585       1  A2AJB7
A2AJK6           NA           NA      NA           NA        NA      NA  A2AJK6
A2AQP0           NA           NA      NA           NA        NA      NA  A2AQP0
A2AWP8           NA           NA      NA           NA        NA      NA  A2AWP8
A6H8H2           NA           NA      NA           NA        NA      NA  A6H8H2
B1AVY7           NA           NA      NA           NA        NA      NA  B1AVY7
```

The table contains the hypothesis testing results for every protein. Notice that some rows contain missing values. This is because data modelling resulted in a `fitError` for some proteins. We refer to another vignette that describes how to deal with `fitErrors`. We can use the table above directly to build a volcano plot using `ggplot2` functionality.

```
ggplot(inference) +
    aes(x = logFC, y = -log10(adjPval)) +
    geom_hline(yintercept = -log10(0.05)) +
    geom_text_repel(data = filter(inference, adjPval < 0.05),
                    aes(label = Protein)) +
    geom_point() +
    ggtitle("Statistical inference on differences between LF and HF (short duration)",
            paste("Hypothesis test:", gsub("ridgeCondition", "", colnames(L)), "= 0"))
```

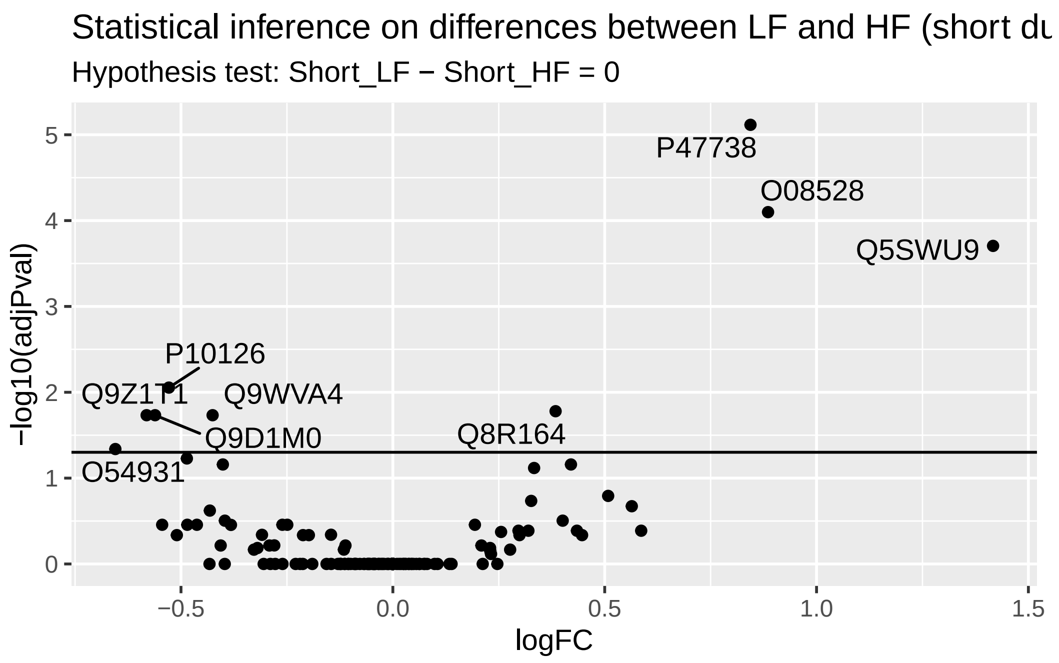

In this example (remember this is a subset of the complete data set), only a few proteins pass the significance threshold of 5%. Let us visualise the protein with the largest fold change.

```
(targetProtein <- rownames(inference)[which.max(inference$logFC)])
```

```
[1] "Q5SWU9"
```

To obtain the required data, we perform a little data manipulation pipeline:

1. We use the `QFeatures` subsetting functionality to retrieve all data related to Q5SWU9 and focusing on the `ions_norm` set that contains the preprocessed peptide ion data used for modelling.
2. We use `longFormat()` to convert the object into a table suitable for plotting.
3. We remove missing values for plotting and focus only on the data with short diet duration.
4. We reorder the sample identifiers to improve visualisation.

```
ionData <- mouse[targetProtein, , "ions_norm"] |> #1
    longFormat(colvars = colnames(colData(mouse)), #2
               rowvars = c("Protein.Accessions", "ionID")) |>
    data.frame() |>
    filter(!is.na(value) & Duration == "Short") |> #3
    mutate(colname = factor(colname, levels = unique(colname[order(Condition)]))) #4
```

We can now plot the log normalised intensities. Since the protein is modelled at the peptide ion level, multiple ion intensities are recorded in each sample. Each ion is linked across samples using a grey line. Samples are coloured according to the diet type. Finally, we split the plot in facets, one for each mixture, to visualise the heterogeneity induced by different pools of mice.

```
ggplot(ionData) +
    aes(x = colname,
        y = value) +
    geom_line(aes(group = ionID), linewidth = 0.1) +
    geom_point(aes(colour = Condition)) +
    facet_grid(~ Mixture, scales = "free") +
    labs(x = "Sample", y = "log2 intensity") +
    ggtitle(targetProtein) +
    theme_minimal() +
    theme(axis.text.x = element_blank())
```

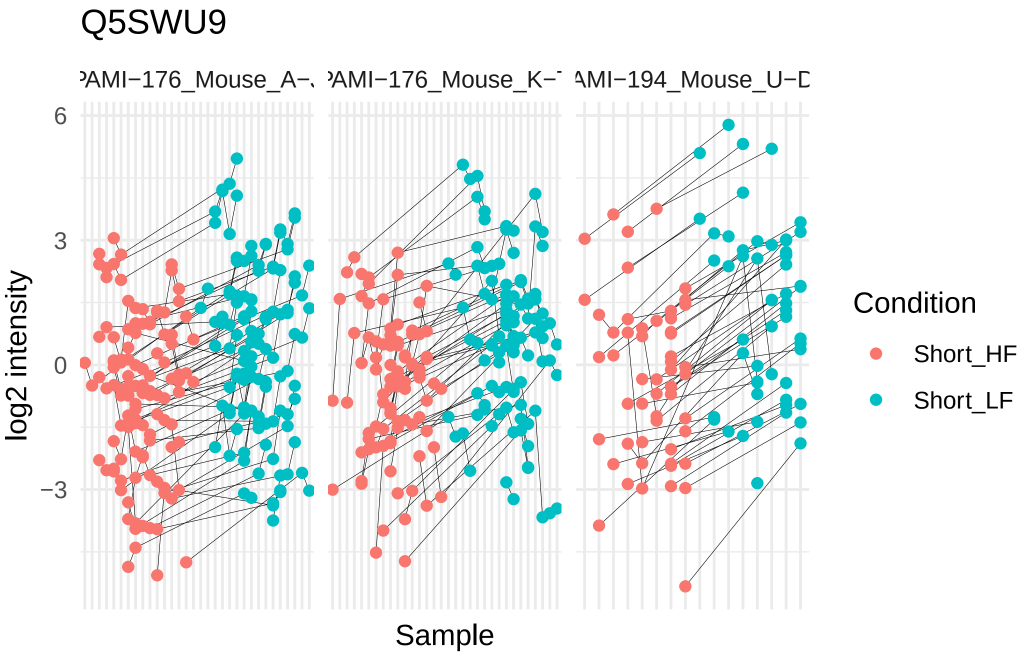

The statistical analysis revealed a significant increase (positive log fold change) of the abundance for Q5SWU9 in the group fed with a low-fat diet compared to the high-fat diet fed group (upon early diet duration). This finding can be visually validated as there is a systematic increase in peptide ion intensities between the low-fat diet group (blue) compared to the high-fat diet group (red).

Difference between low fat and high fat diet after long duration

The second question one can ask is what proteins are affected by diet when only considering, this time, a long diet duration. Following the same approach as above, the contrast becomes.

```
hypothesis2 <- "ridgeConditionLong_LF - ridgeConditionLong_HF = 0"
```

We run the same statistical analysis pipeline as above.

```
L <- makeContrast(
    hypothesis2,
    parameterNames = c("ridgeConditionLong_HF","ridgeConditionLong_LF")
)
mouse <- hypothesisTest(
    mouse, i = "proteins_msqrob", L, modelColumn = "msqrob_psm_rrilmm"
)
inference <- rowData(mouse[["proteins_msqrob"]])[[colnames(L)]]
inference$Protein <- rownames(inference)
```

And we plot the results.

```
ggplot(inference) +
    aes(x = logFC, y = -log10(adjPval)) +
    geom_hline(yintercept = -log10(0.05)) +
    geom_text_repel(data = filter(inference, adjPval < 0.05),
                    aes(label = Protein)) +
    geom_point() +
    ggtitle("Statistical inference on differences between LF and HF (long duration)",
            paste("Hypothesis test:", gsub("ridgeCondition", "", colnames(L)), "= 0"))
```

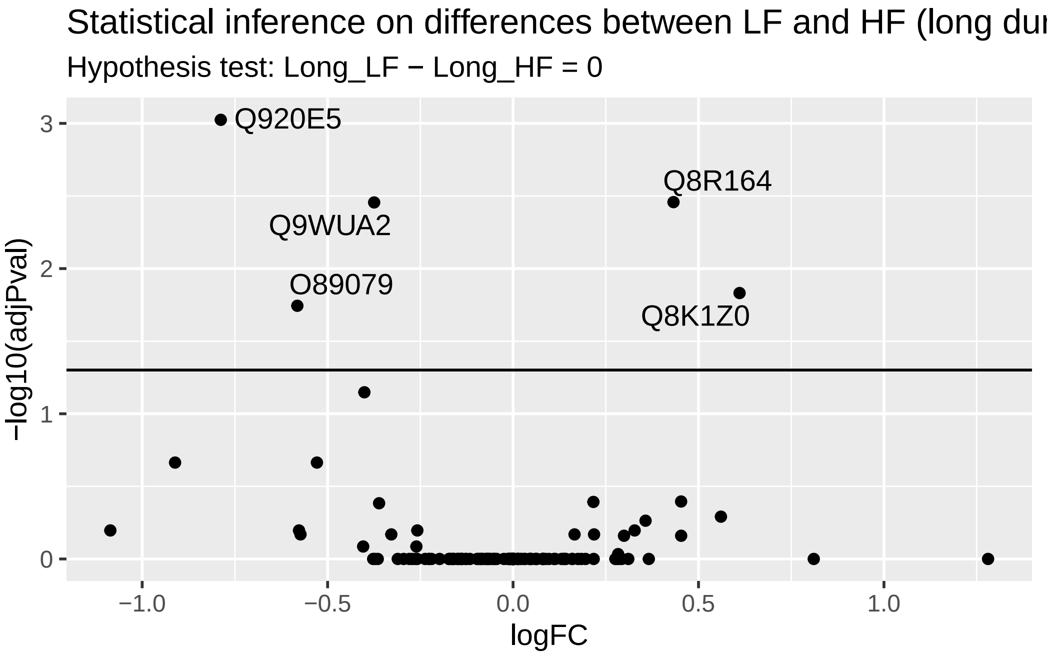

Again, only a few proteins come out differentially abundant between the two diets, but after a long diet duration. Surprisingly, there is only a small overlap between differential protein after short duration and after long duration. One hypothesis is that there is not sufficient data to detect a reliable difference. A solution would be to combine both short and long diet duration to retrieve an averaged systematic effect between diets that combine all available data. Another hypothesis is that diet duration may influence the effect of diet on the protein abundances. We will explore the two hypothesis in the following two sub-sections.

Average difference between low fat and high fat diet

One may want to identify the set of proteins that are systematically differentially abundant between diets, irrespective of the duration. To answer this question, we want to infer on the average difference between group `LF` and group `HF`. So the contrast becomes the difference between the average of group `LF` (short and long duration) and the average of group `HF` (short and long duration).

```
hypothesis3 <- "(ridgeConditionShort_LF + ridgeConditionLong_LF)/2 - (ridgeConditionShort_HF
```

Note that this contrast cannot be estimated by `msTrawler` (O'Brien et al. 2024) which only outputs results for all pairwise comparisons between group levels.

We next run again the same statistical analysis pipeline as above.

```r
L <- makeContrast(
    hypothesis3,
    parameterNames = c("ridgeConditionShort_HF", "ridgeConditionLong_HF", "ridgeConditionSho
)
mouse <- hypothesisTest(
    mouse, i = "proteins_msqrob", L, modelColumn = "msqrob_psm_rrilmm"
)
inference <- rowData(mouse[["proteins_msqrob"]])[[colnames(L)]]
inference$Protein <- rownames(inference)
```

And we plot the results.

```r
ggplot(inference) +
    aes(x = logFC, y = -log10(adjPval)) +
    geom_hline(yintercept = -log10(0.05)) +
    geom_text_repel(data = filter(inference, adjPval < 0.05),
                    aes(label = Protein)) +
    geom_point() +
    ggtitle("Statistical inference on average difference between LF and HF",
            paste("Hypothesis test:", gsub("ridgeCondition", "", colnames(L)), "= 0"))
```

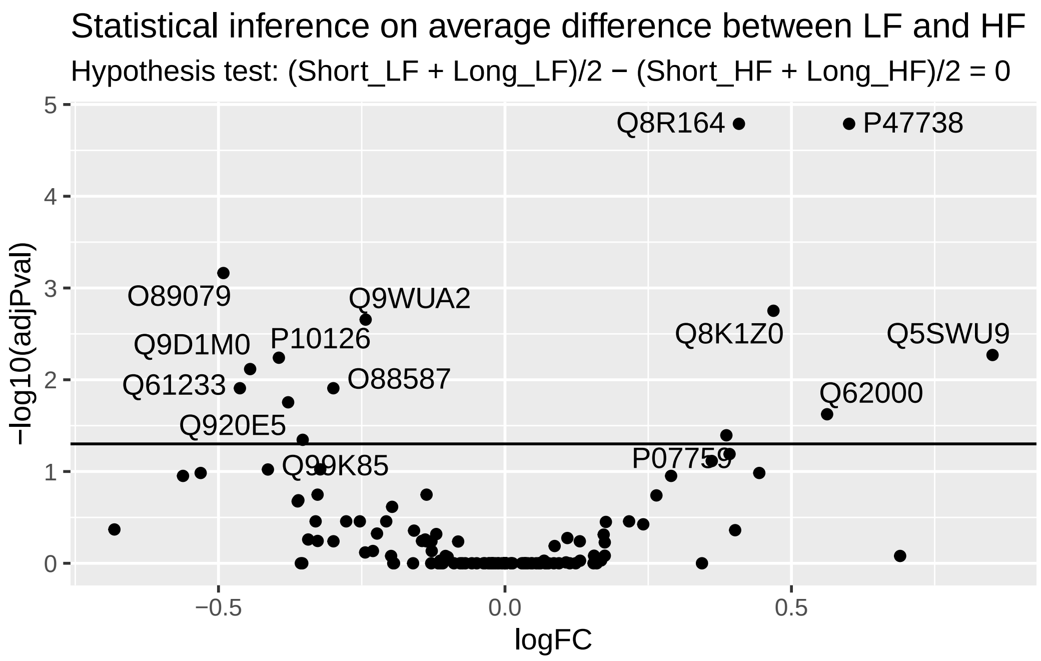

We find much more significant proteins when combining all available data to infer the differences between low-fat and high-fat diets, irrespective of duration. We also retrieve a good

overlap between this set of significant proteins and the two previous sets, indicating that more data helped improving the statistical power.

Interaction: does the diet effect change according to duration?

We will now explore whether the effect of diet on protein abundance may be affected by duration, i.e. we want to infer on the difference of differences. Let our first difference be the average difference (log2 fold change) in low-fat and high-fat diet for short duration and the second difference the average difference in low-fat and high-fat diet for long duration. Since we want to assess if the diet effect, i.e. the average difference (log2 fold change) between low-fat and high-fat diet, changes according to duration, our contrast of interest is the difference between these two differences.

```
hypothesis4 <- " (ridgeConditionShort_LF - ridgeConditionShort_HF) - (ridgeConditionLong_LF -
```

We can proceed with the same statistical pipeline.

```
L <- makeContrast(
    hypothesis4,
    parameterNames = c("ridgeConditionShort_HF", "ridgeConditionLong_HF", "ridgeConditionSho
)
mouse <- hypothesisTest(
    mouse, i = "proteins_msqrob", L, modelColumn = "msqrob_psm_rrilmm"
)
inference <- rowData(mouse[["proteins_msqrob"]])[[colnames(L)]]
inference$Protein <- rownames(inference)
```

And we plot the results.

```
ggplot(inference) +
    aes(x = logFC, y = -log10(adjPval)) +
    geom_hline(yintercept = -log10(0.05)) +
    geom_text_repel(data = filter(inference, adjPval < 0.05),
                    aes(label = Protein)) +
    geom_point() +
    ggtitle("Statistical inference on the effect of duration on the differences between diets
            paste("Hypothesis test:", gsub("ridgeCondition", "", colnames(L)), "= 0"))
```

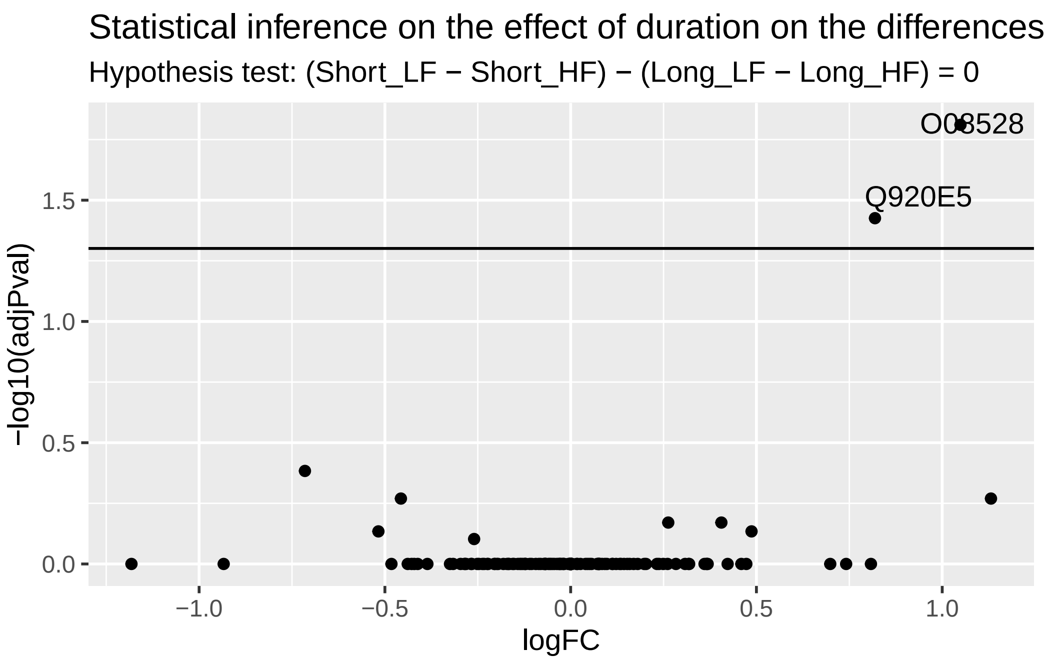

There is only one protein for which duration impacts the effect of diet. Let us visually explore this changes for the most significant protein.

```
(targetProtein <- rownames(inference)[which.min(inference$adjPval)])
```

```
[1] "O08528"
```

We use again `Qfeatures`'s data manipulation pipeline.

```
ionData <- mouse[targetProtein, , "ions_norm"] |> #1
    longFormat(colvars = colnames(colData(mouse)), #2
               rowvars = c("Protein.Accessions", "ionID")) |>
    data.frame() |>
    filter(!is.na(value)) |> #3
    mutate(colname = factor(colname, levels = unique(colname[order(Condition)]))) #4
```

And explore the peptide ion data, this time also facetting for duration in order to highlight changes in direction of the difference between low-fat and high-fat diets.

```
ggplot(ionData) +
    aes(x = Run, #colname,
        y = value) +
```

```
  # geom_line(aes(group = ionID), linewidth = 0.1) +
   #geom_point(aes(colour = Condition)) +
   # geom_boxplot(aes(colour = Condition)) +
   geom_point(aes(shape = rowname, colour = Diet)) +
   facet_wrap(Duration ~ Mixture, scales = "free_x", labeller = label_both) +
   #labs(x = "Sample", y = "log2 intensity") +
   labs(x = "Run", y = "log2 intensity") +
   ggtitle(targetProtein) +
   theme_minimal() +
   theme(axis.text.x = element_blank(),legend.position = "none")
```

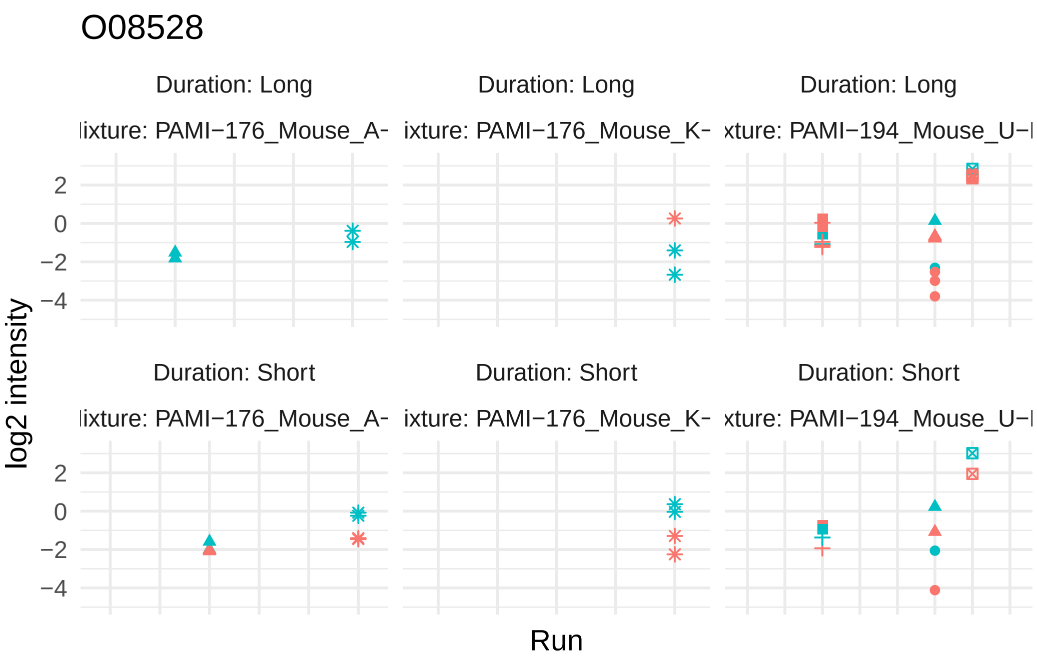

The graph hints towards a slight increase in protein abundance in the low-fat diet group compared to the high-fat diet group during a short diet duration, but this increase disappears after a long diet duration. However, the visual inspection of the results also shows that the result rely on sparse and highly unbalanced data. The results may hence require further experimental validation.

Note that we performed the statistical analysis for each hypothesis separately. However, msqrob2 can assess multiple hypothesis at once.

```
L <- makeContrast(
    c(hypothesis1, hypothesis2, hypothesis3, hypothesis4),
    parameterNames = c("ridgeConditionShort_HF", "ridgeConditionLong_HF", "ridgeConditionSho
```

```
)
mouse <- hypothesisTest(
    mouse, i = "proteins_msqrob", L,
    modelColumn = "msqrob_psm_rrilmm", overwrite = TRUE
)
```

Note that since we already generated results for the contrast, we overwrite the results with the argument `overwrite = TRUE`.

We retrieve the inference tables from the `rowData` to generate the volcano plot.

```
inferenceTables <- rowData(mouse[["proteins_msqrob"]])[, colnames(L)]
```

We here use a `lapply()` loop to generate the plots. The code chunk is elaborate, but follows the same structure as in the previous section. This generates a list of volcano plots, one for each hypothesis.

```
volcanoPlots <- lapply(colnames(inferenceTables), function(i) {
    inference <- inferenceTables[[i]]
    inference$Protein <- rownames(inference)
    ggplot(inference) +
        aes(x = logFC, y = -log10(adjPval)) +
        geom_hline(yintercept = -log10(0.05)) +
        geom_text_repel(data = filter(inference, adjPval < 0.05),
                        aes(label = Protein)) +
        geom_point() +
        ggtitle("Hypothesis test:",
                paste(gsub("ridge", "", i), "= 0"))
})
```

We combine all the plots in a single figure using the `patchwork` packages.

```
wrap_plots(volcanoPlots)
```

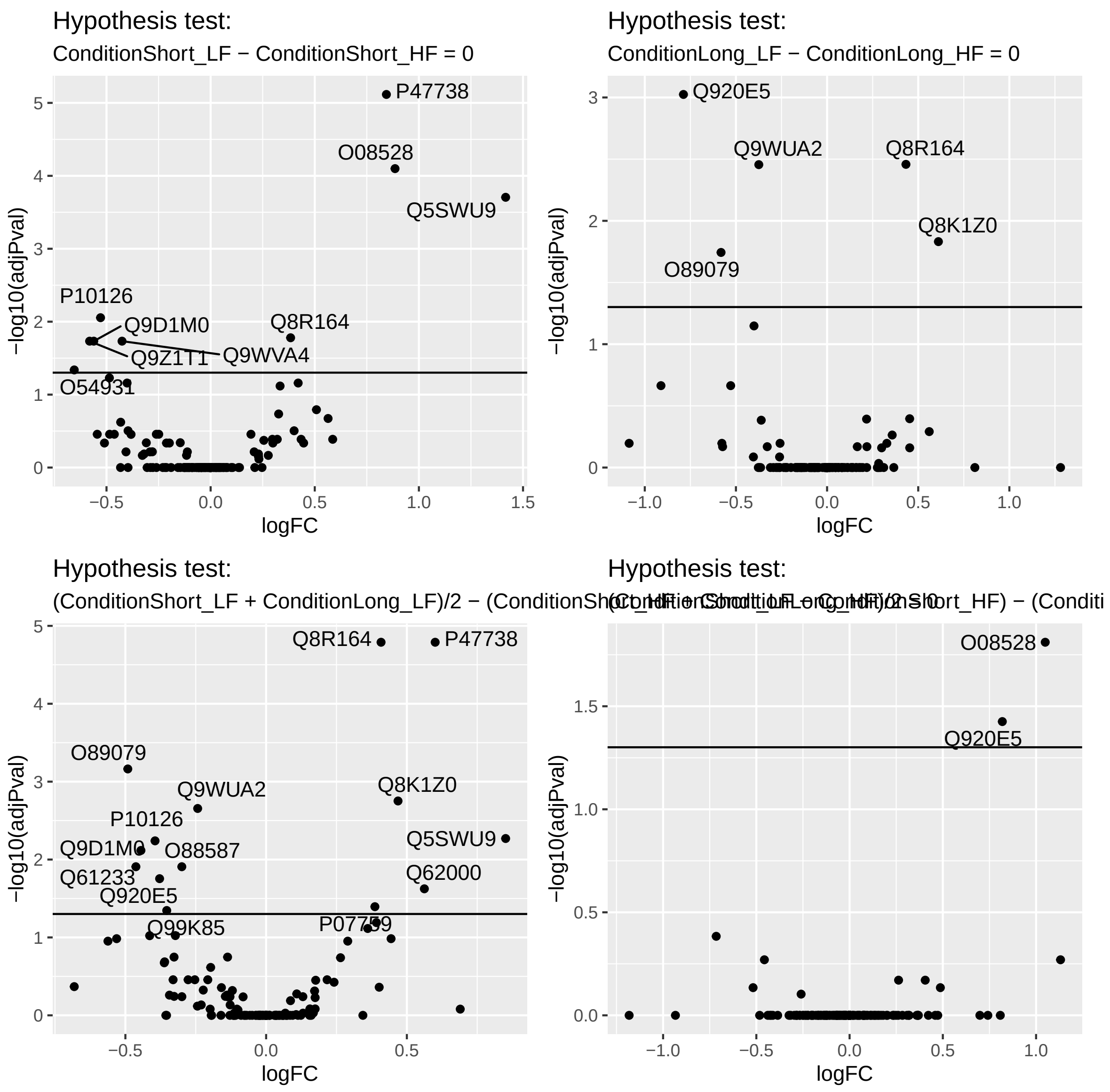

Two-factor model

Running the second model where diet and duration are encoded as separate factors requires the same function as for the first model.

```
mouse <- msqrobAggregate(
    mouse, i = "ions_norm",
    formula = model2,
```

```
    fcol = "Protein.Accessions",
    modelColumnName = "msqrob_psm_rrilmm",
    name = "proteins_msqrob_model2",
    ridge = TRUE, robust = TRUE
)
```

The major change between the two approaches is the interpretation of the parameters. We again make use of `VisualizeDesign()` to help interpreting the parameters of the second model.

```
VisualizeDesign(
    sampleData =  colData(mouse),
    designFormula = ~ Diet * Duration,
    textSizeFitted = 4
)$plotlist[[1]]
```

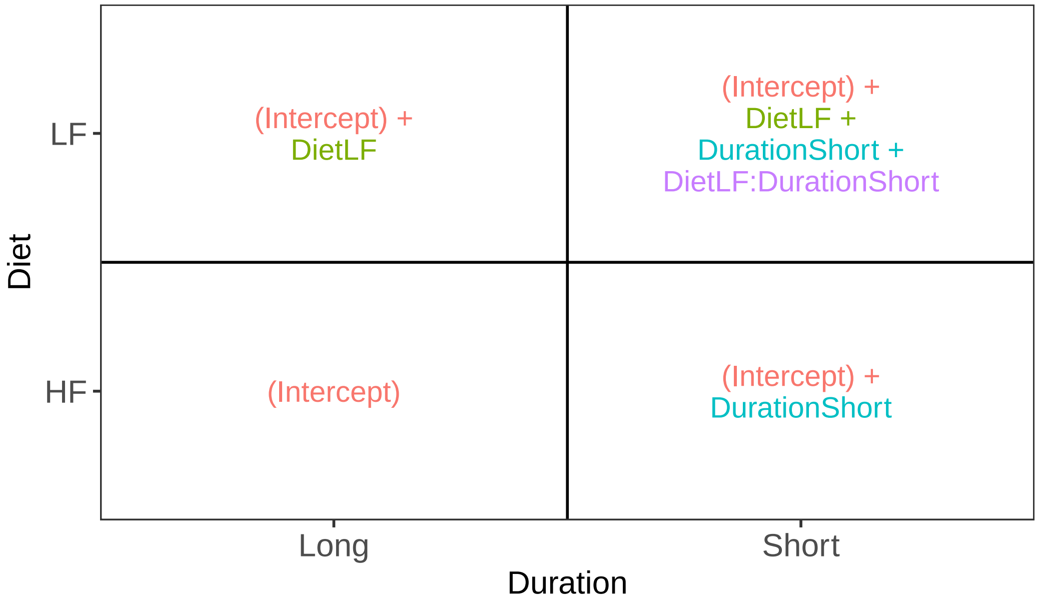

We here construct contrasts for the same hypotheses as in the previous section:

1. Difference between low fat and high fat diet after short duration. The mean for the short low-fat diet group is defined by `(Intercept) + DietLF + DurationShort + DietLF:DurationShort`. The mean for the short high-fat diet group is defined by `(Intercept) + DurationShort`. The difference between the two results in the hypothesis below:

```
hypothesis1_tf <- "ridgeDietLF + ridgeDietLF:DurationShort = 0"
```

2. Difference between low fat and high fat diet after long duration. The mean for the long low-fat diet group is defined by `(Intercept) + DietLF`. That for long high-fat diet group is defined by `(Intercept)`. The difference between the two results in the hypothesis below:

```
hypothesis2_tf <- "ridgeDietLF = 0"
```

3. Average difference between low fat and high fat diet. The average low-fat diet is defined by `((Intercept) + DietLF + DurationShort + DietLF:DurationShort + (Intercept) + DietLF)/2`. The average high-fat diet group is defined by `((Intercept) + DurationShort + (Intercept))/2`. The difference between the two results in the hypothesis below:

```
hypothesis3_tf <- "ridgeDietLF + (ridgeDietLF:DurationShort)/2 = 0"
```

4. Proteins for which the diet effects changes according to location. Remember, this questions assesses the difference between differences. The difference between 1. and 2. is `(DietLF + DietLF:DurationShort) - (DietLF)` and results in the hypothesis below:

```
hypothesis4_tf <- "ridgeDietLF:DurationShort = 0"
```

Notice that hypothesis 4 boils down to inferring the significance of the interaction term, which demonstrates its theoretical definition.

We will again use the same statistical inference pipeline as depicted for the previous model, testing all hypothesis at once.

```
L_tf <- makeContrast(
    c(hypothesis1_tf, hypothesis2_tf, hypothesis3_tf, hypothesis4_tf),
    parameterNames = c("ridgeDietLF","ridgeDurationShort","ridgeDietLF:DurationShort")
)
```

We perform the hypothesis testing pipeline using the same function.

```
mouse <- hypothesisTest(
    mouse, i = "proteins_msqrob_model2", L_tf,
    modelColumn = "msqrob_psm_rrilmm"
)
```

The inference tables were all stored in the `rowData` as separate columns, like previously.

```
inferenceTables2 <- rowData(mouse[["proteins_msqrob_model2"]])[, colnames(L_tf)]
```

We here use again the lapply() loop to generate the list of volcano plots, one for each hypothesis.

```
volcanoPlots2 <- lapply(names(inferenceTables2), function(i) {
    inference <- inferenceTables2[[i]]
    inference$Protein <- rownames(inference)
    ggplot(inference) +
        aes(x = logFC, y = -log10(adjPval)) +
        geom_hline(yintercept = -log10(0.05)) +
        geom_text_repel(data = filter(inference, adjPval < 0.05),
                        aes(label = Protein)) +
        geom_point() +
        ggtitle("Hypothesis test:",
                paste(gsub("tests_|ridge", "", i), "= 0"))
})
```

We combine all the plots in a single figure using the patchwork packages.

```
wrap_plots(volcanoPlots2)
```

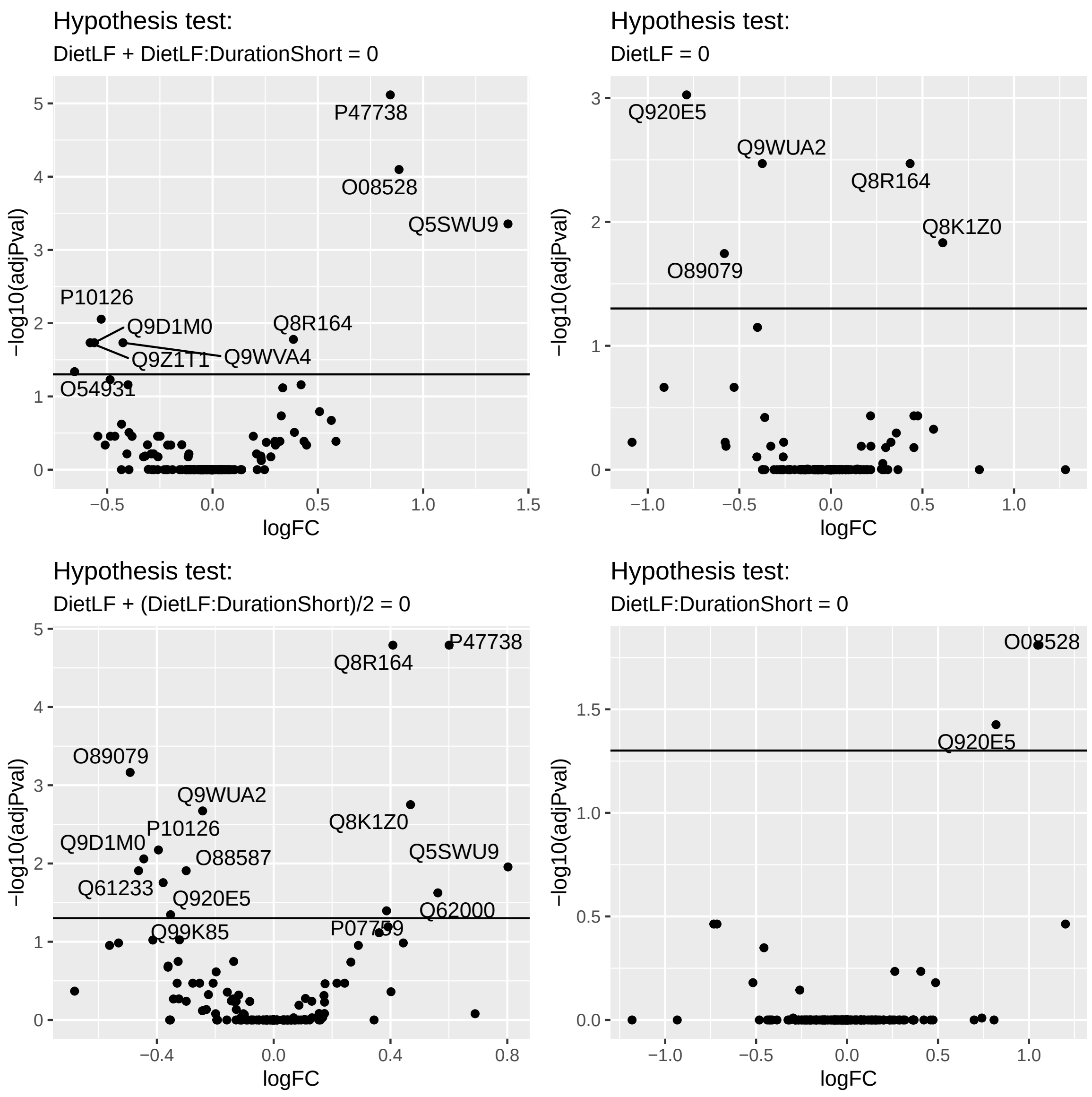

We here confirm that both modelling approach (one-factor or two-factor encoding) lead to the same results, as the volcano plots above are almost identical to the previous section.

Protein-level model

This section illustrate data modelling at the protein level instead of the PSM level as shown in the previous sections. We fit an interaction model similar to model 2.

Here, we work with a workflow where the peptide ion intensities are summarised within each run. This has already been done in the preprocessing. Note, that we no longer have multiple abundance values for a protein in the same channel of a run. Hence, we can omit the nested effects for channel and ionID in run.

```
model2sum <- ~ Diet * Duration + ## fixed effect for Diet and Duration with interaction
        # (1 | Channel) + ## (1) random effect for channel is negligible
        (1 | Mixture) + ## (2) random effect for mixture
        (1 | Run) + ## (3) random effect for MS run
        (1 | BioReplicate)  ## (6) random effect for biorepeat (mouse)
```

For protein-level modelling, we use `msqrob()` instead of `msqrobAggregate()`, but their function arguments closely overlap.

```
mouse <- msqrob(
    mouse, i = "proteins",
    formula = model2sum,
    modelColumnName = "msqrob_rrilmm",
    ridge = TRUE, robust = TRUE
)
```

We perform hypothesis tests for the early, late, average and interaction effects. Note, that the specification of contrasts for the fixed effects remains the same.

```
mouse <- hypothesisTest(
    mouse, i = "proteins", L_tf, modelColumn = "msqrob_rrilmm"
)
```

The inference tables were all stored in the `rowData` as separate columns, like previously.

```
inferenceTablesSum <- rowData(mouse[["proteins"]])[, colnames(L_tf)]
```

We here use again the `lapply()` loop that generates the list of volcano plots, one for each hypothesis.

```
volcanoPlotsSum <- lapply(names(inferenceTablesSum), function(i) {
    inference <- inferenceTablesSum[[i]]
    inference$Protein <- rownames(inference)
    ggplot(inference) +
        aes(x = logFC, y = -log10(adjPval)) +
        geom_hline(yintercept = -log10(0.05)) +
```

```
        geom_text_repel(data = filter(inference, adjPval < 0.05),
                        aes(label = Protein)) +
        geom_point() +
        ggtitle("Hypothesis test:",
                paste(gsub("tests_|ridge", "", i), "= 0"))
})
```

```
wrap_plots(volcanoPlotsSum)
```

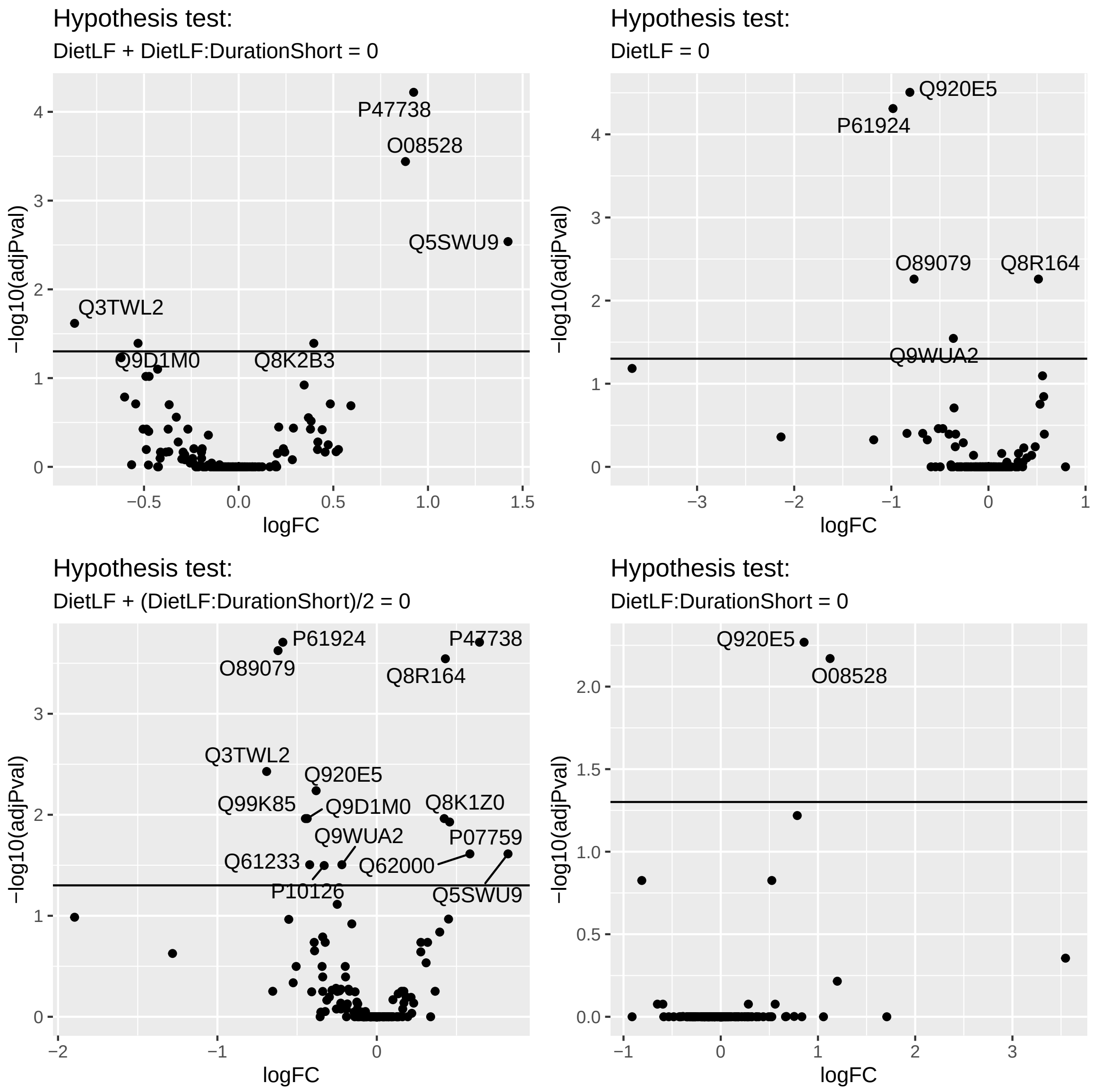

Analysis at the protein-level by summarising over technical fractions

Read QFeatures object

The mouse data consist of 3 TMT-mixtures that have been acquired using 9 technical fractions. Researchers often choose to summarise the peptide ion-intensities to the protein-level over all technical fraction. We will thus ignore the variability due to `Run` and `Fraction` and only consider the `Mixture` as a batch effect.

Note, that we can use the same code to read in the data and setup the colData.

```
psms <- read.delim(psmFile)
coldata <- read.csv(annotFile)
coldata$File.Name <- coldata$Run
coldata$Duration <- gsub("_.*", "", coldata$Condition)
coldata$Diet <- gsub(".*_", "", coldata$Condition)
```

We will again subset the data set to reduce computational costs. If you want to run the vignette on the full data set, you can skip this chunk. We here randomly sample 500 proteins from the experiment.

```
proteinIds <- unique(psms$Protein.Accessions)
set.seed(1234)
psms <- psms[psms$Protein.Accessions %in% sample(proteinIds, 500), ]
```

In order for the `readQFeatures` function to combine the data over all technical fractions we used the following steps:

1. We remove the `Run`, `Fraction` and `File` columns from the sample annotations. These are specific to each fraction and are no longer meaningful when combining data at the mixture level.
2. The entries for each of the 9 fraction remain, leading to duplicated lines which we collapse using `unique()`.
3. We define `Mixture` as the `runCol` variable as expected by `QFeatures` (see `?readQFeatures()` for more details).
4. We add the names of the quantitative columns contained in the PSM table in a new `quantCols` column, again as expected by `QFeatures`.
5. We add a new column in the PSM table that contains the name of the mixture so they match with the `runCol` from *3.*. This information is retrieved from the raw spectrum file names.

```
coldata <- coldata |>
  select(-Run,-Fraction,-File.Name) |> ## 1.
  unique() |> ## 2.
  mutate(
      runCol = Mixture, ## 3.
      quantCols = paste0("Abundance..", Channel) ## 4.
  )
psms$Mixture <- sub("(.*)_TMT.*", "\\1", psms$Spectrum.File)  ## 5.
```

We now have prepared the data so that `readQFeatures()` reads the data with each mixture (hence combining the nine fractions) in a separate set.

```
(mouseMix <- readQFeatures(psms, colData = coldata,
                       quantCols = unique(coldata$quantCols),
                       runCol = "Mixture", name = "psms"))
```

```
An instance of class QFeatures containing 3 assays:
 [1] PAMI-176_Mouse_A-J: SummarizedExperiment with 3424 rows and 10 columns
 [2] PAMI-176_Mouse_K-T: SummarizedExperiment with 3081 rows and 10 columns
 [3] PAMI-194_Mouse_U-Dd: SummarizedExperiment with 4089 rows and 10 columns
```

PSM filtering

We now proceed to the same PSM filtering approach as presented above using the same code. Therefore, we perform the steps in a single code chunk. See the *Data preprocessing* section for comprehensive walkthrough.

```
## Remove failed protein inference and protein groups
mouseMix <- filterFeatures(
    mouseMix, ~ Protein.Accessions != "" &
        !grepl(";", Protein.Accessions))
## Filter missing values
mouseMix <- zeroIsNA(mouseMix, names(mouseMix))
mouseMix <- filterNA(mouseMix, names(mouseMix), pNA = 0.7)
## Keep the highest intensity PSM for each peptide ion
for (i in names(mouseMix)) {
    rowdata <- rowData(mouseMix[[i]])
    rowdata$ionID <- paste0(rowdata$Annotated.Sequence, rowdata$Charge)
    rowdata$rowSums <- rowSums(assay(mouseMix[[i]]), na.rm=TRUE)
    rowdata <- rowdata <- data.frame(rowdata) |>
```

```
        group_by(ionID) |>
        mutate(psmRank = rank(-rowSums))
    rowData(mouseMix[[i]]) <- DataFrame(rowdata)
}
mouseMix <- filterFeatures(mouseMix, ~ psmRank == 1)
mouseMix <- filterFeatures(mouseMix, ~ rowSums > 0)
## Assign unique rownames for data integration
for(i in names(mouseMix)){
    rownames(mouseMix[[i]]) <- rowData(mouseMix[[i]])$ionID ## 2.
}
```

Preprocessing workflow

The same applies for the preprocessing workflow. Again, see the *Data preprocessing* section for comprehensive walkthrough.

```
sNames <- names(mouseMix)
## Log-transformation
mouseMix <- logTransform(
    mouseMix, sNames, name = paste0(sNames, "_log"), base = 2
)
## Normalisation
mouseMix <- normalize(
    mouseMix, paste0(sNames, "_log"), name = paste0(sNames, "_norm"),
    method = "center.median"
)
## Summarisation to protein level
mouseMix <- aggregateFeatures(
    mouseMix, i = paste0(sNames, "_norm"), name = paste0(sNames, "_proteins"),
    fcol = "Protein.Accessions", fun = MsCoreUtils::medianPolish,
    na.rm=TRUE
)
## Remove unnecessary files
mouseMix <- subsetByColData(
    mouseMix, mouseMix$Condition != "Norm" & mouseMix$Condition != "Long_M"
)
## Data integration
mouseMix <- joinAssays(mouseMix, paste0(sNames, "_proteins"), "proteins") ## 2.
```

Statistical modelling

We summarised the data over all technical fraction runs. So we have to model the effect of the treatment while correcting for the correlation of multiple biorepeats that are assessed in the same mixture.

```
model2sumMix <- ~ Diet * Duration + ## fixed effect for Diet and Duration with interaction
    # (1 | Channel) + ## (1) random effect for channel is negligible
    (1 | Mixture)  ## (2) random effect for mixture
```

We run the protein-level model.

```
mouseMix <- msqrob(
    mouseMix, i = "proteins",
    formula = model2sumMix,
    modelColumnName = "msqrob_rrilmm",
    ridge = TRUE, robust = TRUE
)
```

We perform hypothesis tests for the early, late, average and interaction effects. Note, that the specification of contrasts for the fixed effects remains the same.

```
mouseMix <- hypothesisTest(
    mouseMix, i = "proteins", L_tf, modelColumn = "msqrob_rrilmm"
)
```

The inference tables were all stored in the `rowData` as separate columns, like previously.

```
inferenceTablesSumMix <- rowData(mouseMix[["proteins"]])[, colnames(L_tf)]
```

We here use again the `lapply()` loop that generates the list of volcano plots, one for each hypothesis.

```
volcanoPlotsSumMix <- lapply(names(inferenceTablesSumMix), function(i) {
    inference <- inferenceTablesSumMix[[i]]
    inference$Protein <- rownames(inference)
    ggplot(inference) +
        aes(x = logFC, y = -log10(adjPval)) +
        geom_hline(yintercept = -log10(0.05)) +
        geom_text_repel(data = filter(inference, adjPval < 0.05),
                        aes(label = Protein)) +
```

```
        geom_point() +
        ggtitle("Hypothesis test:",
                paste(gsub("tests_|ridge", "", i), "= 0"))
})
```

```
wrap_plots(volcanoPlotsSumMix)
```

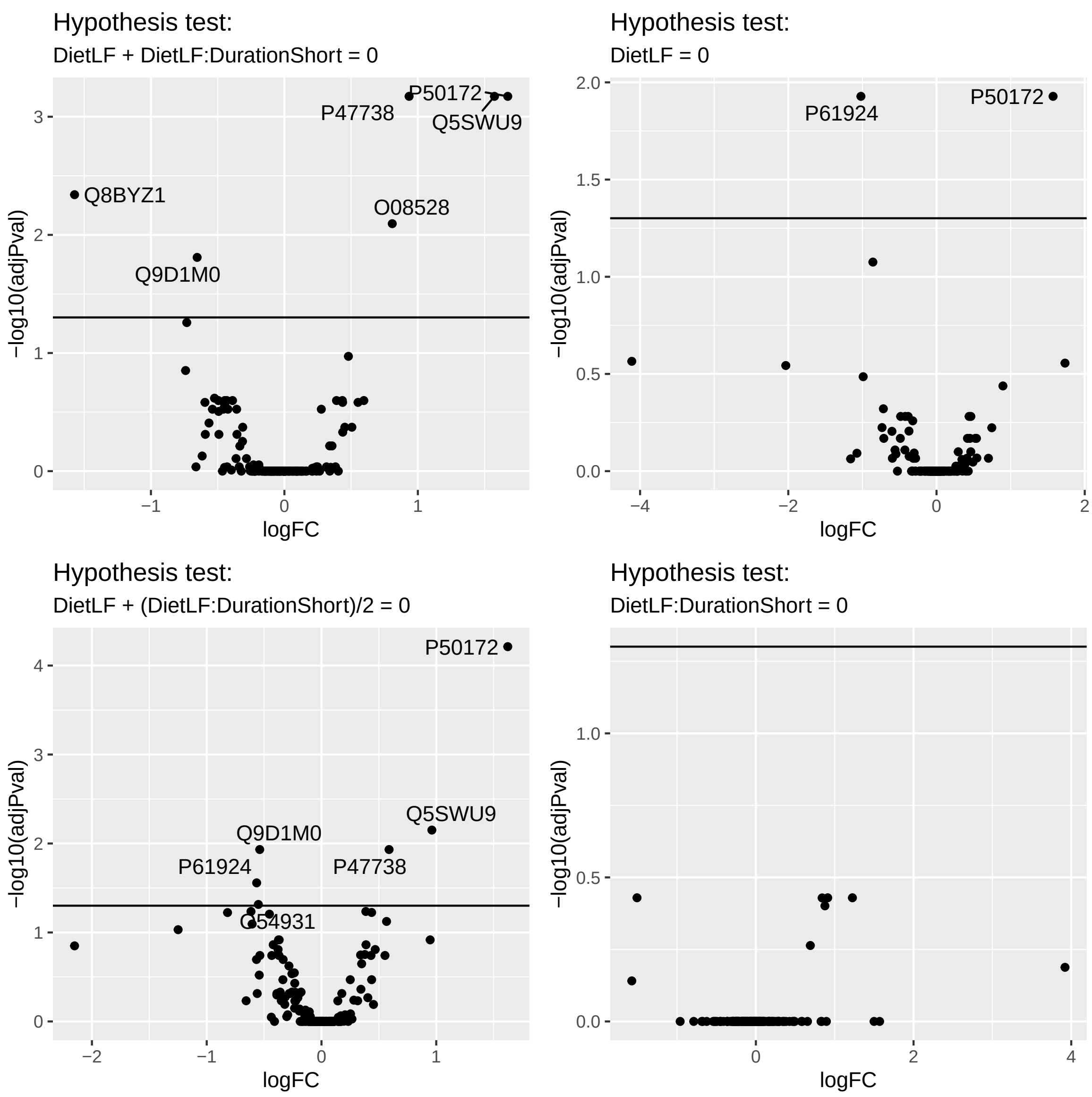

We can see the results partly overlap with the previous protein-level analysis where fractions

were modelled separately. Since there is no ground truth available, we cannot conclude which approach is best.

Conclusion

In this vignette, we have demonstrated the application of msqrob2TMT workflows on a real-life case study.

The preprocessing workflow relies on the the `QFeatures` package. The package provides functionality to carry out many steps like data filtering, missing values management, normalisation, log-transformation, imputation, summarisation, etc. The functions also provide different methods for each step, meaning that the preprocessing pipeline can be easily adapted to the researcher's needs based on their experiment and data set.

Once preprocessed, we use the `msqrob2` package to model all sources of variability as identified from the experiment: effect of diet and duration, effect of the MS acquisition run, effect of TMT mixture, effect of spectrum, and effect of sample. Modelling these different sources of variability allows to correctly infer changes in protein abundances between groups of interest while using PSM-level data, although we also illustrate how to model the data at the protein level.

Regarding protein-level modelling, we also demonstrated two summarisation strategies by collapsing peptides within each LC fraction separately or by collapsing peptides within a mixture, hence combining the peptides across the nine fractions. While the first approach allows to account for run-to-run variation, it will consider peptides that span two fractions (because their chromatogram is split during fractionation) as independent. Conversely, summarisation across fractions within a mixture will consistently combine data originating from the same chromatogram, but ignores the run-to-run variation leading to increased noise. In practice, we found the two approaches led to partially overlapping results, and we could not objectively establish which approach is best suited.

The experiment aims to understand the proteomic changes in mouse adipose tissue that occur upon feeding the mice with low-fat or high-fat diets, during a short or a long duration. We illustrated two approaches to encode these treatments of interest. First, we reproduced the approach taken by MSstatsTMT (Huang et al. 2020) that combines diet and duration into a single factor. This is because MSstatsTMT can only assess ANOVA designs with one factor, but we also showed how to model these data as two factor using our msqrob2TMT workflow. To allow for the diet duration to influence the impact of diet type, we included an interaction term. We showed that both approaches are equivalent and lead to the same results. However, the second approach is far more flexible and allows including more than 2 variables (with multiple interaction terms) as well as including numerical variables, which may be essential in other experimental contexts.

When performing statistical analysis, we explained how to translate biological questions to statistical hypothesis and contrast matrices. For example, we explored the differences in protein abundance between low-fat and high-fat diets and the impact of diet duration on the difference between low-fat and high-fat diets. Some contrasts involved several parameters, which is not possible to perform with `msTrawler` (O'Brien et al. 2024).

Hence, we here demonstrated the power and flexibility of `msqrob2` and the msqrob2TMT workflows to help researchers answer biologically-relevant questions from their MS proteomics data.

Citation

> Vandenbulcke S, Vanderaa C, Crook O, Martens L, Clement L. msqrob2TMT: robust linear mixed models for inferring differential abundant proteins in labelled experiments with arbitrarily complex design. bioRxiv. Published online March 29, 2024:2024.03.29.587218. doi:10.1101/2024.03.29.587218

License

This vignette is distributed under a Artistic-2.0 license.

Session info

```
R version 4.4.2 (2024-10-31)
Platform: x86_64-pc-linux-gnu
Running under: Ubuntu 24.04.1 LTS

Matrix products: default
BLAS:   /usr/lib/x86_64-linux-gnu/openblas-pthread/libblas.so.3
LAPACK: /usr/lib/x86_64-linux-gnu/openblas-pthread/libopenblasp-r0.3.26.so;  LAPACK version 3

locale:
 [1] LC_CTYPE=en_US.UTF-8       LC_NUMERIC=C
 [3] LC_TIME=en_US.UTF-8        LC_COLLATE=en_US.UTF-8
 [5] LC_MONETARY=en_US.UTF-8    LC_MESSAGES=en_US.UTF-8
 [7] LC_PAPER=en_US.UTF-8       LC_NAME=C
 [9] LC_ADDRESS=C               LC_TELEPHONE=C
[11] LC_MEASUREMENT=en_US.UTF-8 LC_IDENTIFICATION=C
```

```
time zone: Etc/UTC
tzcode source: system (glibc)

attached base packages:
[1] stats4    stats     graphics  grDevices utils     datasets  methods
[8] base

other attached packages:
 [1] ExploreModelMatrix_1.18.0   BiocFileCache_2.14.0
 [3] dbplyr_2.5.0                BiocParallel_1.40.0
 [5] patchwork_1.3.0             ggrepel_0.9.6
 [7] ggplot2_3.5.1               dplyr_1.1.4
 [9] msqrob2_1.14.1              QFeatures_1.16.0
[11] MultiAssayExperiment_1.32.0 SummarizedExperiment_1.36.0
[13] Biobase_2.66.0              GenomicRanges_1.58.0
[15] GenomeInfoDb_1.42.1         IRanges_2.40.1
[17] S4Vectors_0.44.0            BiocGenerics_0.52.0
[19] MatrixGenerics_1.18.1       matrixStats_1.5.0

loaded via a namespace (and not attached):
 [1] Rdpack_2.6.2            DBI_1.2.3               rlang_1.1.4
 [4] magrittr_2.0.3          shinydashboard_0.7.2    clue_0.3-66
 [7] compiler_4.4.2          RSQLite_2.3.9           vctrs_0.6.5
[10] reshape2_1.4.4          stringr_1.5.1           ProtGenerics_1.38.0
[13] pkgconfig_2.0.3         crayon_1.5.3            fastmap_1.2.0
[16] XVector_0.46.0          labeling_0.4.3          promises_1.3.2
[19] rmarkdown_2.29          UCSC.utils_1.2.0        nloptr_2.1.1
[22] purrr_1.0.2             bit_4.5.0.1             xfun_0.50
[25] zlibbioc_1.52.0         cachem_1.1.0            jsonlite_1.8.9
[28] blob_1.2.4              later_1.4.1             DelayedArray_0.32.0
[31] parallel_4.4.2          cluster_2.1.8           R6_2.5.1
[34] stringi_1.8.4           limma_3.62.2            boot_1.3-31
[37] Rcpp_1.0.14             knitr_1.49              BiocBaseUtils_1.8.0
[40] httpuv_1.6.15           Matrix_1.7-1            splines_4.4.2
[43] igraph_2.1.3            tidyselect_1.2.1        abind_1.4-8
[46] yaml_2.3.10             codetools_0.2-20        curl_6.1.0
[49] lattice_0.22-6          tibble_3.2.1            plyr_1.8.9
[52] shiny_1.10.0            withr_3.0.2             evaluate_1.0.3
[55] pillar_1.10.1           BiocManager_1.30.25     filelock_1.0.3
[58] DT_0.33                 shinyjs_2.1.0           reformulas_0.4.0
[61] generics_0.1.3          munsell_0.5.1           scales_1.3.0
[64] minqa_1.2.8             xtable_1.8-4            BiocStyle_2.34.0
[67] glue_1.8.0              lazyeval_0.2.2          tools_4.4.2
```

```
[70] lme4_1.1-36            cowplot_1.1.3          grid_4.4.2
[73] tidyr_1.3.1            rbibutils_2.3          MsCoreUtils_1.18.0
[76] colorspace_2.1-1       nlme_3.1-166           GenomeInfoDbData_1.2.13
[79] cli_3.6.3              S4Arrays_1.6.0         AnnotationFilter_1.30.0
[82] gtable_0.3.6           rintrojs_0.3.4         digest_0.6.37
[85] SparseArray_1.6.0      htmlwidgets_1.6.4      farver_2.1.2
[88] memoise_2.0.1          htmltools_0.5.8.1      lifecycle_1.0.4
[91] httr_1.4.7             mime_0.12              statmod_1.5.0
[94] bit64_4.5.2            MASS_7.3-64
```
