# Supplementary material for: msqrob2TMT: Robust Linear Mixed Models for Inferring Differential Abundant Proteins in Labeled Experiments With Arbitrarily Complex Design
Source: Mol Cell Proteomics. 2025 May 30;24(7):101002. doi: 10.1016/j.mcpro.2025.101002 (PMC12365501; doi:10.1016/j.mcpro.2025.101002)
Supplement: Supplemental Data 3 [file mmc3.pdf]

Msqrob2TMT: flexible workflows for modelling labelled proteomics data

Christophe Vanderaa Stijn Vandenbulcke Lieven Clement

2025-01-31

Table of contents

Introduction 2

Load packages 3

Load data 3

Data preprocessing 5
- Sample filtering . . . 5
- PSM filtering . . . 6
- Missing data management . . . 8
- Log2 transformation . . . 8
- Normalisation . . . 8
- Summarisation . . . 9
- Join assays . . . 9

Modelling sources of variation 9
- Effect of treatment of interest . . . 10
- Effect of TMT channel and run . . . 11
- Effect of replication . . . 12
- PSM-level modelling . . . 13

Statistical inference 14
- Retrieving results . . . 14
- Volcano plots . . . 17
- Fold change distributions . . . 18
- Visual validation . . . 19
- Protein-level models . . . 20

Inference for all pairwise comparisons between conditions 23

Dealing with `fitErrors` **27**
Removing the random effect of sample 27
Manual inspection 30
Imputation 33

Conclusion **35**

Citation **36**

License **36**

Session info **36**

Introduction

Labelling strategies in mass spectrometry (MS)-based proteomics enable increased sample throughput by acquiring multiplexed samples in a single run. However, contemporary designs often require the acquisition of multiple runs, leading to a complex correlation structure. Addressing this correlation is key for correct statistical inference and reliable biomarker discovery. msqrob2TMT is a set of mixed model-based workflows tailored toward differential abundance analysis for labelled MS-based proteomics data. Thanks to its increased flexibility, msqrob2TMT can model both sample-specific and feature-specific (e.g. peptide or protein) covariates, which unlocks the inference to experiments with arbitrarily complex designs as well as to correct explicitly for feature-specific properties.

The key features of `msqrob2TMT` workflows are:

1. Modularity: all core functions rely on the `QFeatures` class, a standardised data structure, meaning that output of a function can be fed as input to any other function. Hence, different functions are assembled as modular blocks into a complete data analysis workflows that can be easily adapted to the pecularities of any MS-based proteomics data set. Therefore, the approach extends well beyond the use case presented in this vignette.
2. Flexibility: the `msqrob2` modelling approach relies on the `lme4::lmer()` model specification syntax, meaning that any linear model can be specified. For fixed effects, this includes modelling categorical and numerical variables, as well as their interaction. Unlike MSstatsTMT and msTrawler we also put no requirement on the presence of specific variables to include, although we strongly suggest a few below. Hence, `msqrob2` can model any arbitrarily complex linear model.
3. Performance: thanks to the inclusion of robust ridge regression, we demonstrated improved performance of msqrob2TMT workflows upon the competing software.

You can read more about `msqrob2TMT` in:

> Vandenbulcke S, Vanderaa C, Crook O, Martens L, Clement L. msqrob2TMT: robust linear mixed models for inferring differential abundant proteins in labelled experiments with arbitrarily complex design. bioRxiv. Published online March 29, 2024:2024.03.29.587218. doi:10.1101/2024.03.29.587218

In this vignette, we will guide you through how to implement `msqrob2TMT` workflows using the `msqrob2` package.

Load packages

First, we load the `msqrob2` package, along 2 additional packages for data manipulation and visualisation.

```
library("msqrob2")
library("dplyr")
library("ggplot2")
```

`msqrob2` relies on parallelisation to improve computational speed. To ensure this vignette can be run regardless of hardware, we will disable parallelisation. Parallelisation is controlled using the `BiocParallel` package.

```
library("BiocParallel")
register(SerialParam())
```

Load data

The data set used in this vignette is a spike-in experiment, published in Huang et al. 2020 (PXD0015258). It consists of controlled mixtures with known ground truth. UPS1 peptides at concentrations of 500, 333, 250, and 62.5 fmol were spiked into 50 g of SILAC HeLa peptides, each in duplicate. These concentrations form a dilution series of 1, 0.667, 0.5, and 0.125 relative to the highest UPS1 peptide amount (500 fmol). A reference sample was created by combining the diluted UPS1 peptide samples with 50g of SILAC HeLa peptides. All dilutions and the reference sample were prepared in duplicate, resulting in a total of ten samples. These samples were then treated with TMT10-plex reagents and combined before LC-MS/MS analysis. This protocol was repeated five times, each with three technical replicates, totaling 15 MS runs.

The data have been deposited by the authors in the `MSV000084264` MASSiVE repository, but we will retrieve the timestamped data from our Zenodo repository. To facilitate management

of the files, we here download them using the BiocFileCache package. We need 2 files: the Skyline identification and quantification table generated by the authors and the sample annotation files. BiocFileCache ensures that the files are downloaded once, hence the chunk below will take some time only the first time you run it.

```
library("BiocFileCache")
bfc <- BiocFileCache()
psmFile <- bfcrpath(bfc, "https://zenodo.org/records/14767905/files/spikein1_psms.txt?downloa
annotFile <- bfcrpath(bfc, "https://zenodo.org/records/14767905/files/spikein1_annotations.c
```

Now the files are downloaded, we can load the two tables. We also perform a little cleanup of the sample annotations to generate the information needed for downstream data processing.

```
psms <- read.delim(psmFile)
coldata <- read.csv(annotFile)
coldata <- coldata[, c("Run", "Channel", "Condition", "Mixture", "TechRepMixture")]
coldata$FileName <- coldata$Run
coldata$Run <- sub("^.*(Mix.*).raw", "\\1", coldata$Run)
```

There is a peculiarity with the dataset: the spectra have been identified with 2 nodes. In one node, the authors searched the SwissProt database for proteins with static modifications related to the metabolic labelling, in the other node they searched the Sigma_UPS protein database without these static modifications. However, some spectra were identified by both nodes leading to duplicate PSMs. We here remove these duplicated PSMs that are identification artefacts.

```
qcols <- grep("Abundance", colnames(psms), value = TRUE)
duplicatesQuants <- duplicated(psms[, qcols]) | duplicated(psms[, qcols], fromLast = TRUE)
psms <- psms[!duplicatesQuants, ]
```

We will also subset the data set to reduce computational costs. If you want to run the vignette on the full data set, you can skip this chunk. The subsetting will keep all UPS proteins, known to be differentially abundant by experimental design and we will keep 500 background proteins known to be unchanged across condition.

```
allProteins <- unique(psms$Protein.Accessions)
upsProteins <- grep("ups", allProteins, value = TRUE)
helaProteins <- grep("ups", allProteins, value = TRUE, invert = TRUE)
set.seed(1234)
keepProteins <- c(upsProteins, sample(helaProteins, 500))
psms <- psms[psms$Protein.Accessions %in% keepProteins, ]
```

Finally, we combine the data into a `QFeatures` object. We add two annotation columns so that the `readQFeatures` function can link the PSM to the corresponding quantification column within each run, see `?readQFeatures()` for more details.

```
coldata$runCol <- coldata$FileName
coldata$quantCols <- paste0("Abundance..", coldata$Channel)
spikein <- readQFeatures(psms, colData = coldata,
                         quantCols = unique(coldata$quantCols),
                         runCol = "Spectrum.File", name = "psms")
names(spikein) <- sub("^.*(Mix.*).raw", "\\1", names(spikein))
spikein
```

```
An instance of class QFeatures containing 15 assays:
 [1] Mixture1_01: SummarizedExperiment with 1905 rows and 10 columns
 [2] Mixture1_02: SummarizedExperiment with 1902 rows and 10 columns
 [3] Mixture1_03: SummarizedExperiment with 1952 rows and 10 columns
 ...
 [13] Mixture5_01: SummarizedExperiment with 1919 rows and 10 columns
 [14] Mixture5_02: SummarizedExperiment with 1909 rows and 10 columns
 [15] Mixture5_03: SummarizedExperiment with 1844 rows and 10 columns
```

We now have a `QFeatures` object with 15 sets, each containing data associated with an MS run.

Data preprocessing

`msqrob2` relies on the `QFeatures` data structure, meaning that we can directly make use of `QFeatures`' data preprocessing functionality. We will not detail the usage of each function below, but instead refer to the `QFeatures` documentation.

Sample filtering

We first remove the reference channels. These channels were used by the MSstatsTMT authors to obtain normalisation factors. However, this approach ignores the uncertainty associated with the measurement with these channels, potentially inflating the noise in the samples of interest. Hence, msqrob2TMT workflows do not use the reference channels. In practice, we found no impact of reference channel normalisation on model performance. The information is available from the `colData`, under the `Condition` column. We remove any sample that is marked as `Norm`.

```
spikein <- subsetByColData(spikein, spikein$Condition != "Norm")
```

PSM filtering

The background proteins originate from HeLa cells, which also contain UPS proteins. The background UPS proteins and the spiked-in UPS proteins differ in metabolic labelling, so we should be able to distinguish them. We used the PSM-level data searched with mascot, as provided by the MSstatsTMT authors who used two mascot identification nodes. In one node they searched the SwissProt database for proteins with static modifications related to the metabolic labelling, in the other node they searched the Sigma_UPS protein database without these static modifications. Ideally, this should separate the spiked-in UPS proteins and the UPS proteins from the HeLa cells, however, this is not always the case. The SwissProt search is expected to return peptide-spectrum matches (PSMs) for all proteins, including non-UPS HeLa, UPS HeLa, and spike-in UPS proteins. Conversely, the Sigma_UPS search is expected to return PSMs exclusively for spike-in UPS proteins. However, a PSM that matches a UPS protein in the SwissProt search but is not identified as such in the Sigma_UPS search could either correctly originate from a HeLa protein or represent a spiked-in UPS protein that was not recognised as such in the Sigma_UPS search. Additionally, there are ambiguous PSMs that are not matched to a UPS protein in the HeLa search but are matched to a UPS protein in the SwissProt search. To address this, we exclude these ambiguous proteins from the analysis.

To define amibiguous PSMs, we retrieve the PSM annotations from the `rowData` and create a new colum indicating whether a PSM belongs to a UPS protein or not, based on the protein SwissProt identifiers.

```
rowdata <- rbindRowData(spikein, names(spikein))
rowdata$isUps <- "no"
isUpsProtein <- grepl("ups", rowdata$Protein.Accessions)
rowdata$isUps[isUpsProtein] <- "yes"
```

Then, we define an ambiguous PSM as a PSM that is marked as UPS by the SwissProt identifier but not by the Sigma_UPS node (`Marked.as` column), and inversely. Ambiguous PSMs are removed.

```
rowdata$isUps[!isUpsProtein & grepl("UPS", rowdata$Marked.as)] <- "amb"
rowdata$isUps[isUpsProtein & !grepl("UPS", rowdata$Marked.as)] <- "amb"
rowData(spikein) <- split(rowdata, rowdata$assay)
spikein <- filterFeatures(spikein, ~ isUps != "amb")
```

Next, we remove PSMs that could not be mapped to a protein or that map to multiple proteins (the protein identifier contains multiple identifiers separated by a `;`).

```
spikein <- filterFeatures(
    spikein, ~ Protein.Accessions != "" & ## Remove failed protein inference
        !grepl(";", Protein.Accessions)) ## Remove protein groups
```

We also remove peptide ions that map to a different protein depending on the run.

```
rowdata <- rbindRowData(spikein, names(spikein))
rowdata <- data.frame(rowdata) |>
    group_by(Annotated.Sequence, Charge) |>
    mutate(nProtsMapped = length(unique(Protein.Accessions)))
rowData(spikein) <- split(rowdata, rowdata$assay)
spikein <- filterFeatures(spikein, ~ nProtsMapped == 1)
```

We also remove proteins that can only be found in one run as such proteins may not be trustworthy.

```
idProteins <- lapply(rowData(spikein), function(x) unique(x$Protein.Accessions))
idInNRuns <- table(unlist(idProteins))
oneRunWonders <- names(idInNRuns)[idInNRuns == 1]
spikein <- filterFeatures(spikein, ~ !Protein.Accessions %in% oneRunWonders)
```

Finally, peptide ions that were identified with multiple PSMs in a run are collapsed to the PSM with the highest summed intensity over the channels, a strategy that is also used by MSstats.

We therefore

1. Make a new variable for ionID in the rowData.
2. We calculate the `rowSums` for each ion.
3. Make a new variable `psmRank` that ranks the PSMs for each ionID based on the summed intensity.
4. We store the new information back in the `rowData`.
5. For each ion that maps to multiple PSMs, only keep the PSM with the highest summed intensity, that is that ranks first.
6. Filter ions for which the rowSum equals 0.

```
for (i in names(spikein)) {
    rowdata <- rowData(spikein[[i]])
    rowdata$ionID <- paste0(rowdata$Annotated.Sequence, rowdata$Charge) ## 1.
    rowdata$rowSums <- rowSums(assay(spikein[[i]]), na.rm=TRUE) ## 2.
    rowdata <- data.frame(rowdata) |>
```

```
        group_by(ionID) |>
        mutate(psmRank = rank(-rowSums)) ## 3.
    rowData(spikein[[i]]) <- DataFrame(rowdata) ## 4.
}
spikein <- filterFeatures(spikein, ~ psmRank == 1) ## 5.
spikein <- filterFeatures(spikein, ~ rowSums > 0) ## 6.
```

Missing data management

We ensure missing values are properly encoded. Zero values and missing values have different interpretations. However, zeros may artificially occur when the quantification threshold lies above the noise level. We therefore replace any zero by a missing value. We then remove PSMs with 5 or more missing values out of 10 TMT channels (>= 50%). This is an arbitrary value that may need to be adjusted depending on the experiment and the data set.

```
spikein <- zeroIsNA(spikein, names(spikein))
spikein <- filterNA(spikein, names(spikein), pNA = 0.5)
```

Log2 transformation

Next, we log2 transform the intensities.

```
sNames <- names(spikein)
spikein <- logTransform(
    spikein, sNames, name = paste0(sNames, "_log"), base = 2
)
```

Normalisation

We normalise each TMT channel by subtracting the median intensity within each run.

```
spikein <- normalize(
    spikein, paste0(sNames, "_log"), name = paste0(sNames, "_norm"),
    method = "center.median"
)
```

Summarisation

In case of the proteins models, we summarise (also referred to as aggregation) the PSM-level data into protein intensities through median polish.

```
spikein <- aggregateFeatures(
    spikein, i = paste0(sNames, "_norm"), name = paste0(sNames, "_proteins"),
    fcol = "Protein.Accessions", fun = MsCoreUtils::medianPolish,
    na.rm = TRUE
)
```

Join assays

Finally, we join the different runs into a single set for ions and for proteins. Sets are joined by stacking the columns (samples) in a matrix and rows (features) are matched by the row names. We will join all log2-normalised ion data in one set and all protein data in another. First, we need to redefine the rownames for the ion data using the ion identifier.

```
for (i in paste0(sNames, "_norm")) {
    rownames(spikein[[i]]) <- rowData(spikein[[i]])$ionID
}
```

We can now join the sets together.

```
spikein <- joinAssays(spikein, paste0(sNames, "_norm"), "ions")
spikein <- joinAssays(spikein, paste0(sNames, "_proteins"), "proteins")
```

Modelling sources of variation

Proteomics data contain several sources of variation that need to be accounted for by the model. The code below shows how to run the model that accounts for all relevant sources of variation in the spike-in experiment, which we have shown performs best in the Msqrob2TMT paper.

```
spikein <- msqrobAggregate(
    spikein,  i = "ions",
    formula = ~ 0 + Condition + ## fixed effect for experimental condition
        # (1 | Channel) + ## random effect for channel
        (1 | Mixture) + ## random effect for mixture
```

```
        (1 | Run) + ## random effect for run
        (1 | Run:Channel) + ## random effect for PSMs for the same protein in a channel of a
        (1 | Run:ionID), ## random effect for ions in the same spectrum of an MS run
    fcol = "Protein.Accessions",
    modelColumnName = "msqrob_psms_rrilm",
    robust =TRUE, ridge = TRUE
)
```

Note, that we use an encoding without intercept `~ 0 +`. This makes it more straightforward to define contrasts for one-way ANOVA designs with a treatment involving a single factor. Indeed, by suppressing the intercept, a model parameters is estimated for each group. Otherwise, `msqrob2` selects one of the groups as the reference group, for which its model parameter is absorbed in the intercept.

We will now build up the model by progressively adding the different sources of variation.

Effect of treatment of interest

We model the source of variation induced by the experimental treatment of interest as a fixed effect, which we consider non-random, i.e. the treatment effect is assumed to be the same in repeated experiments, but it is unknown and has to be estimated. When modelling a typical label-free experiment at the protein level, the model boils down to a linear model, again we suppress the index for protein:

$$y_r = \mathbf{x}_r^T \beta + \epsilon_r,$$

with y_r the $\log_2$-normalized protein intensities in run r; $\mathbf{x}_r$ a vector with the covariate pattern for the sample in run r encoding the intercept, treatment, potential batch effects and confounders; β the vector of parameters that model the association between the covariates and the outcome; and ϵ_r the residuals reflecting variation that is not captured by the fixed effects. Note that $\mathbf{x}_r$ allows for a flexible parameterization of the treatment beyond a single covariate, i.e. including a 1 for the intercept, continuous and categorical variables as well as their interactions. For all models considered in this work, we assume the residuals to be independent and identically distributed (i.i.d) according to a normal distribution with zero mean and constant variance, i.e. $\epsilon_r \sim N(0, \sigma_\epsilon^2)$, that can differ from protein to protein.

Using `msqrob2`, the model translates into the following code:

```
spikein <- msqrob(
    spikein,  i = "proteins",
    formula = ~  0 + Condition, ## fixed effect for experimental condition
    robust = TRUE, ridge = TRUE,
```

```
    modelColumnName = "msqrob_rrilm"
)
```

The function takes the `QFeatures` object, extracts the quantitative values from the `"proteins"` set generated during summarisation, and fits a simple linear model with `Condition` as covariate, which is automatically retrieved from `colData(spikein)`. We also enabled M-estimation (`robust = TRUE`) for improved robustness against outliers and ridge penalisation (`ridge = TRUE`) to stabilise the parameter estimation. The fitting results are available in the `msqrob_rrilm` column of the `rowData`. More specifically, the function will store the modelling output for each protein in a `statModel` object, which are then saved in the `rowData`, one model per row. We will see in a later section how to perform statistical inference on the estimated parameters

```
models <- rowData(spikein[["proteins"]])[["msqrob_rrilm"]]
models[1:3]
```

```
$O00151
Object of class "StatModel"
The type is "lmer"
There number of elements is 5

$O00299
Object of class "StatModel"
The type is "lmer"
There number of elements is 5

$O00410
Object of class "StatModel"
The type is "lmer"
There number of elements is 5
```

Note, that we use an encoding without intercept `~ 0 +`. This makes it more straightforward to define contrasts for one-way ANOVA designs with a treatment involving a single factor. Indeed, by suppressing the intercept, a model parameters is estimated for each group. Otherwise, `msqrob2` selects one of the groups as the reference group, for which its model parameter is absorbed in the intercept.

Effect of TMT channel and run

As label-free experiments contain only a single sample per run, run-specific effects will be absorbed in the residuals. However, the data analysis of labeled experiments, e.g. using TMT

multiplexing, involving multiple MS runs has to account for run- and label-specific effects, explicitly. If all treatments are present in each run, and if channel swaps are performed so as to avoid confounding between channel and treatment, then the model parameters can be estimated using fixed channel and run effects. Indeed, for these designs run acts as a blocking variable as all treatment effects can be estimated within each run.

However, for more complex designs this is no longer possible and the uncertainty in the estimation of the mean model parameters can involve both within and between channel and run variability. For these designs we can resort to mixed models where the channel and run effect are modelled using random effects, i.e. they are considered as a random sample from the population of all possible runs (channel labels), which are assumed to be i.i.d normally distributed with mean 0 and constant variance, $u_r \sim N(0, \sigma^{2,\text{run}})$ ($u_{channel} \sim N(0, \sigma^{2,\text{channel}})$). The use of random effects thus models the correlation in the data, explicitly. Indeed, protein intensities that are measured within the same run (channel) will be more similar than protein intensities between runs (channels).

Hence, the model is extended to:

$$y_{rc} = \mathbf{x}^T_{rc}\beta + u_c^{\text{channel}} + u_r^{\text{run}} + \epsilon_{rc}$$

with y_{rc} the normalised $\log_2$ protein intensities in run r and channel c, u_c^{channel} the effect introduced by the label of channel c, and u_r^{run} the effect for MS run r.

This translates in the following code:

```
spikein <- msqrob(
    spikein,  i = "proteins",
    formula = ~  0 + Condition + ## fixed effect for experimental condition
        # (1 | Channel) + ## random effect for channel
        (1 | Run), ## random effect for MS run
    robust = TRUE, ridge = TRUE,
    modelColumnName = "msqrob_rrilmm"
    )
```

Note here that we have commented out the random effect for channel. In practice, normalisation already removes part of the channel effect and is sufficient. You can experiment this yourself by removing the comment sign `#` in front of `(1 | Channel) +` across the vignette, and see how the results may change.

Effect of replication

Some experiments also include technical replication where a TMT mixture can be acquired multiple times. This again will induce correlation. Indeed, protein intensities from the same

mixture will be more alike than those of different mixtures. Hence, we also include a random effect to account for this pseudo-replication, i.e. $u_m^{\text{mix}} \sim N(0, \sigma^{2,\text{mix}})$. The model thus extends to:

$$y_{rcm} = \mathbf{x}_{rcm}^T \beta + u_c^{\text{channel}} + u_r^{\text{run}} + u_m^{\text{mix}} + \epsilon_{rcm}$$

with m the index for mixture.

The model translates to the following code:

```
spikein <- msqrob(
    spikein,  i = "proteins",
    formula = ~  0 + Condition + ## fixed effect for experimental condition
        # (1 | Channel) + ## random effect for channel
        (1 | Run) + ## random effect for MS run
        (1 | Mixture), ## random effect for mixture
    robust = TRUE, ridge = TRUE,
    modelColumnName = "msqrob_rrilmm",
    overwrite = TRUE
)
```

We use `overwrite = TRUE` to overwrite the previous results in the `rowData`.

PSM-level modelling

Above, we modelled the data at the protein level. However, we could also directly estimate the treatment effect from PSM-level data. This will again induce additional levels of correlation. Indeed, the intensities for the different reporter ions in a TMT run within the same spectrum (PSM) will be more similar than the intensities between PSMs. We therefore need to add a random effect term to account for the within PSM correlation structure, i.e. $u_{rp}^{\text{PSM}} \sim N(0, \sigma^{2,\text{PSM}})$. Moreover, in each channel of a run multiple PSM intensities are picked up for each protein. Hence, intensities from different PSMs for a protein in the same channel of a run will be more alike than intensities of different PSMs for the same protein between channels of runs, and we will address this correlation with a channel-specific random effect nested in run, i.e. $u_{rc}^{channel} \sim N(0, \sigma^{2,\text{channel}})$. The model then becomes:

$$y_{rcmp} = \mathbf{x}_{rcmp}^T \beta + u_c^{\text{channel}} + u_r^{\text{run}} + u_m^{\text{mix}} + u_{rc}^{\text{channel}} + u_{rp}^{\text{PSM}} + \epsilon_{rcmp}$$

with y_{rcmp} the $\log_2$-normalized PSM intensities for run r with label c in mixture m and peptide ion p. Note, that the peptide ion random effect is also nested within each run since each spectrum is described by run-specific characteristics.

The model translates to the code below. Note that we here no longer use `msqrob()`, but `msqrobAggregate()`. The latter will combine annotations from the `colData` (i.e. `"Condition"`, `"Channel"`, `"Run"`, `"Mixture"`) and from the `rowData` (i.e. `"ionID"`). Moreover, we need to tell the function how the PSM-level data is grouped to protein data through the `fcol` argument, here we will group PSMs by the `Protein.Accessions`.

```
spikein <- msqrobAggregate(
    spikein,  i = "ions",
    formula = ~  0 + Condition + ## fixed effect for experimental condition
        # (1 | Channel) + ## random effect for channel
        (1 | Run) + ## random effect for Run
        (1 | Mixture) + ## random effect for mixture
        (1 | Run:Channel) + ## random effect for channel nested in run
        (1 | Run:ionID), ## random effect for ion nested in run
    fcol = "Protein.Accessions",
    modelColumnName = "msqrob_psm_rrilmm",
    name = "proteins_msqrob",
    robust = TRUE, ridge = TRUE
)
```

So, we built the model shown at the beginning of this section, effectively accounting for all sources of variation in TMT-based proteomics data.

Statistical inference

Now we have modelled our data, we can use the model output to assess the amplitude of an effect of interest and its statistical significance.

Retrieving results

With `getCoef()`, we can retrieve the estimated model parameters. We will focus on the last model as an illustration, but the same approach applies for any model estimated by `msqrob()` or `msqrobAggregate()`. We start with extracting the model output, stored as `StatModel` objects, from the `rowData`. Next, `getCoef()` retrieves the estimated model parameters:

```
models <- rowData(spikein[["proteins_msqrob"]])$msqrob_psm_rrilmm
params <- getCoef(models[[1]])
head(params)
```

```
                       (Intercept) (Intercept)Run:ChannelMixture1_01:127C
                        -0.9131324                              0.0000000
(Intercept)Run:ChannelMixture1_01:127N (Intercept)Run:ChannelMixture1_01:128C
                         0.0000000                              0.0000000
(Intercept)Run:ChannelMixture1_01:128N (Intercept)Run:ChannelMixture1_01:129C
                         0.0000000                              0.0000000
```

The PSM model estimated 212 parameters to model the fixed and random effects. However, we are interested, for this data set, in the parameters that model the effect of `Condition`.

```
params[grep("Condition", names(params))]
```

```
ridgeCondition0.125   ridgeCondition0.5 ridgeCondition0.667     ridgeCondition1
        0.046231349        -0.007111620        -0.006361725        -0.032758004
```

Note the `"ridge"` tag in front of the parameter names that indicates the parameters have been estimated using ridge penalisation. For this protein we can see that the effects of condition are very close to zero, as expected since the protein is part of the HeLa background. However, we can explore the parameters for one of the UPS proteins.

```
params <- getCoef(models[["O00762ups"]])
params[grep("Condition", names(params))]
```

```
ridgeCondition0.125   ridgeCondition0.5 ridgeCondition0.667     ridgeCondition1
        -1.39371019          0.02427225          0.42348830          0.92014019
```

Let's know verify the result provide the expected fold change. To do so we define a contrast, that is a parameter combination that provide an answer to the research question, for instance for the average log2 difference in intensity between condition 1x and condition 0.5x. Since this is a benchmark study, the obvious answer is $log_2(1) - log_2(0.5) = 1$.

```
unname(params["ridgeCondition1"] - params["ridgeCondition0.5"])
```

```
[1] 0.8958679
```

This value is close to the expected value. Now, the question is how statistically significant is the estimated log2 fold change. `msqrob2` provides a user friendly interface to allow computing contrast of interest and performing statistical inference. We first define the contrast using `makeContrast()`. The first argument is the null hypothesis to test, here that the average difference between condition 1x and 0.5x is 0. The second argument points to the parameters that are involved to perform the test.

```r
(L <- makeContrast(
    "ridgeCondition1 - ridgeCondition0.5 = 0",
    c("ridgeCondition1", "ridgeCondition0.5")
))
```

```
                  ridgeCondition1 - ridgeCondition0.5
ridgeCondition1                                     1
ridgeCondition0.5                                  -1
```

We can now test our null hypothesis using `hypothesisTest()` which takes the `QFeatures` object with the fitted model and the contrast we just built. Again, the results are stored in the set containing the model, here `proteins_msqrob`

```r
spikein <- hypothesisTest(
    spikein, i = "proteins_msqrob", contrast = L,
    modelColumn = "msqrob_psm_rrilmm"
)
```

Let's retrieve the results from the `rowData`.

```r
htest <- rowData(spikein[["proteins_msqrob"]])$"ridgeCondition1 - ridgeCondition0.5"
head(htest)
```

```
               logFC           se         df             t       pval
O00151 -2.564638e-02 2.151045e-02  473.82313 -1.192276e+00 0.23374996
O00220  1.574596e-02 5.935274e-02   94.62092  2.652946e-01 0.79135965
O00244 -3.846217e-11 1.737203e-06   84.20039 -2.214029e-05 0.99998239
O00299 -3.224517e-02 1.179043e-02 1379.86119 -2.734859e+00 0.00632058
O00330 -2.898931e-02 3.675144e-02  193.96129 -7.887940e-01 0.43119548
O00399           NA           NA         NA            NA         NA
           adjPval
O00151 0.56558630
O00220 1.00000000
O00244 1.00000000
O00299 0.03353122
O00330 0.87073669
O00399         NA
```

The last row is filled with missing values because data modelling resulted in a `fitError`. We will explore in a later section how we can deal with proteins that could not be fit.

Volcano plots

We can use the table above directly to build a volcano plot using `ggplot2` functionality. We also highlight which proteins are UPS standards, known from the experimental design to be differentially abundant.

```
htest$protein <- rownames(htest)
htest$isUps <- grepl("ups", htest$protein)
ggplot(htest) +
    aes(x = logFC,
        y = -log10(adjPval),
        color = isUps) +
    geom_hline(yintercept = -log10(0.05)) +
    geom_point() +
    scale_color_manual(
        values = c("grey20", "firebrick"), name = "",
        labels = c("HeLA background", "UPS standard")
    ) +
    ggtitle("msqrob_psm_rrilm model",
            "Hypothesis test: Condition 1x - 0.5x = 0")
```

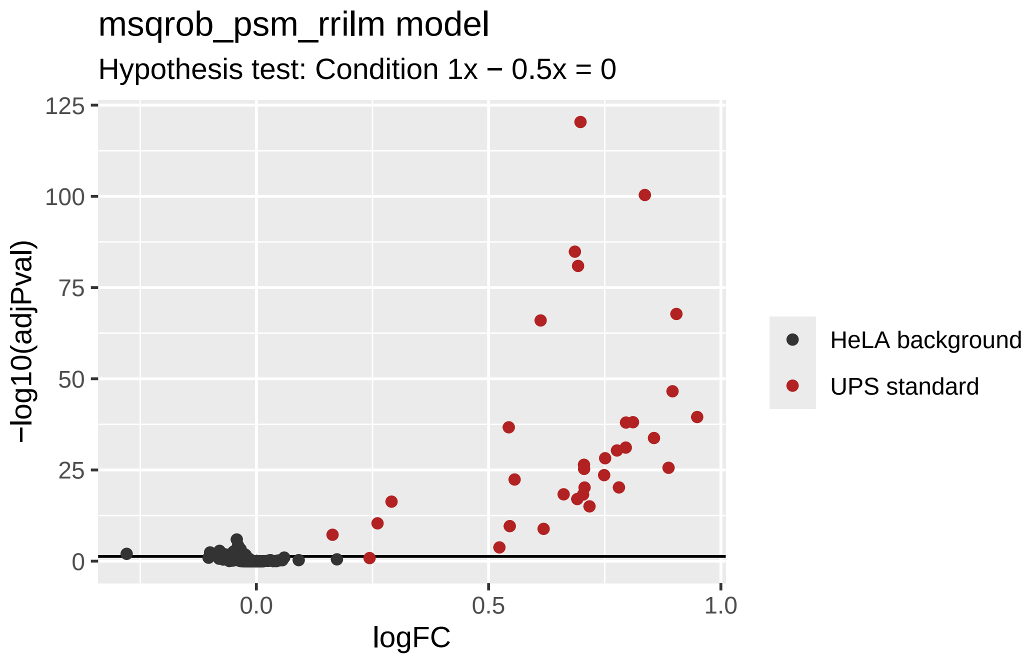

Fold change distributions

As this is a spike-in study with known ground truth, we can also plot the log2 fold change distributions against the expected values, in this case 0 for the HeLa proteins and 1 for the UPS standards.

```
ggplot(htest) +
    aes(y = logFC,
        x = isUps,
        colour = isUps) +
    geom_boxplot() +
    geom_hline(yintercept = c(0, 1), colour = c("grey20", "firebrick")) +
    scale_color_manual(
        values = c("grey20", "firebrick"), name = "",
        labels = c("HeLA background", "UPS standard")
    ) +
    ggtitle("Distribution of the log2 fold changes",
            "Hypothesis test: Condition 1x - 0.5x = 0")
```

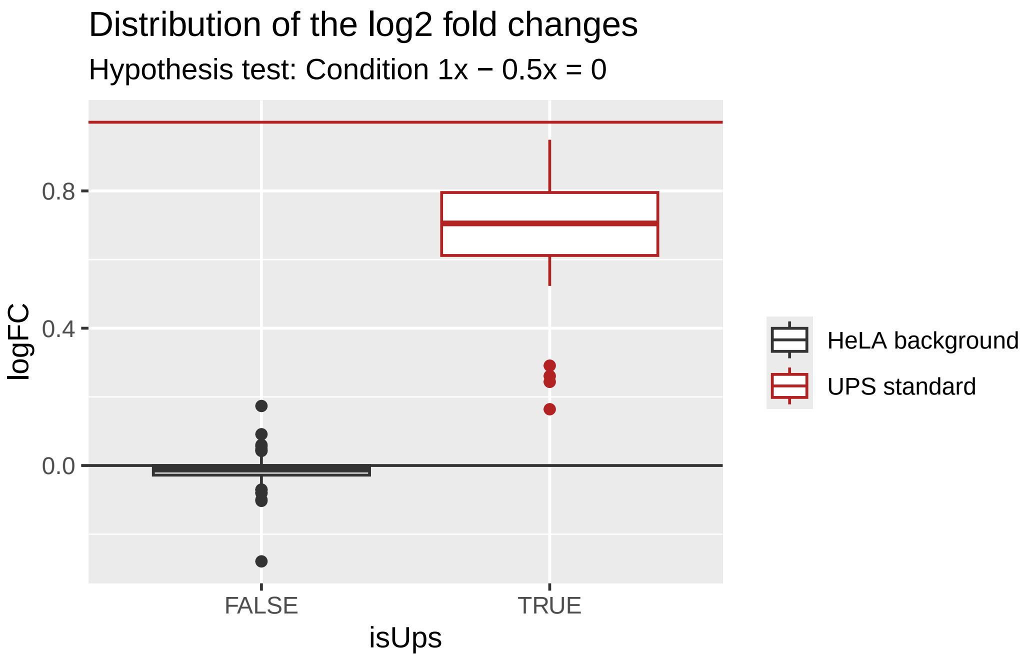

Estimated log2 fold change for HeLa proteins are closely distributed around 0, as expected. log2 fold changes for UPS standard proteins are distributed toward 1, although it is underestimated as reported previously (Savitski et al. 2011).

Visual validation

We can explore the PSM intensities for a protein to validate the statistical inference results. For example, let's explore the intensities for the protein with the most significant difference.

```
(targetProtein <- rownames(htest)[which.min(htest$adjPval)])
```

```
[1] "P02788ups"
```

To obtain the required data, we perform a little data manipulation pipeline:

1. We use the `QFeatures` subsetting functionality to retrieve all data related to P02788ups and focusing on the `ions` set that contains the peptide ion data used for model fitting.
2. We use `longFormat()` to convert the object into a table suitable for plotting.
3. We remove missing values for plotting.
4. We reorder the sample identifiers to improve visualisation.

```
ionData <- spikein[targetProtein, , "ions"] |> #1
    longFormat(colvars = colnames(colData(spikein)), #2
               rowvars = c("Protein.Accessions", "ionID")) |>
    data.frame() |>
    filter(!is.na(value)) |> #3
    mutate(colname = factor(colname, levels = unique(colname[order(Condition)]))) #4
```

Finally, we plot the log2 normalised intensities for each sample. Since the protein is modelled at the peptide ion level, multiple ion intensities are recorded in each sample. Each ion is linked across samples using a grey line. Samples are colored according to UPS spike-in condition. Finally, we split the plot in facets, one for each mixture, to visualise the heterogeneity induced by sample preparation.

```
ggplot(ionData) +
    aes(x = colname,
        y = value) +
    geom_line(aes(group = ionID), linewidth = 0.1) +
    geom_point(aes(colour = Condition)) +
    facet_grid(~ Mixture, scales = "free") +
    ggtitle(targetProtein) +
    theme_minimal() +
    theme(axis.text.x = element_blank())
```

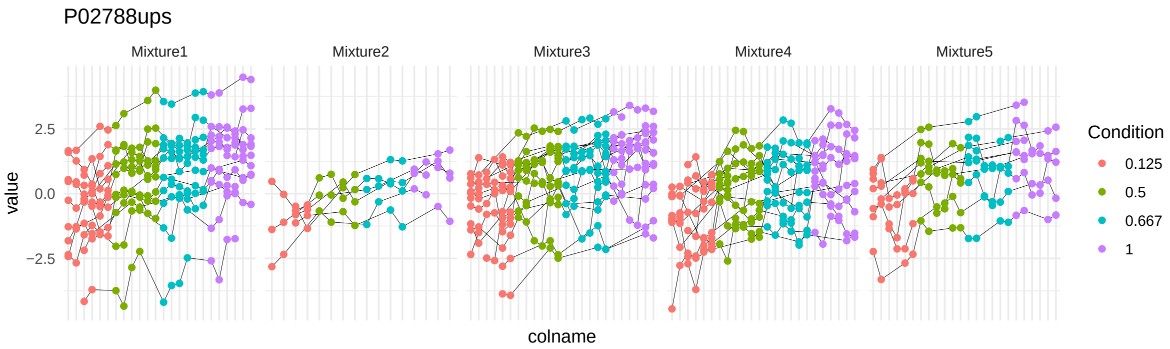

Protein-level models

Performing the statistical inference for summarisation-based models, hence modelling protein-level data, is very similar as for the PSM-based models shown above. Let's apply the same statistical inference pipeline for the `msqrob_rrilmm` model stored in the `QFeatures` object.

```
L <- makeContrast(
    "ridgeCondition1 - ridgeCondition0.5 = 0",
    c("ridgeCondition1", "ridgeCondition0.5")
)
spikein <- hypothesisTest(
    spikein, i = "proteins", contrast = L,
    modelColumn = "msqrob_rrilmm"
)
htestProt <- rowData(spikein[["proteins"]])$"ridgeCondition1 - ridgeCondition0.5"
```

We build the volcano using the same code:

```
htestProt$protein <- rownames(htestProt)
htestProt$isUps <- grepl("ups", htestProt$protein)
ggplot(htestProt) +
    aes(x = logFC,
        y = -log10(adjPval),
        color = isUps) +
    geom_hline(yintercept = -log10(0.05)) +
    geom_point() +
    scale_color_manual(
        values = c("grey20", "firebrick"), name = "",
        labels = c("HeLA background", "UPS standard")
    ) +
```

```
    ggtitle("msqrob_rrilm model",
            "Hypothesis test: Condition 1x - 0.5x = 0\nProtein-level modelling")
```

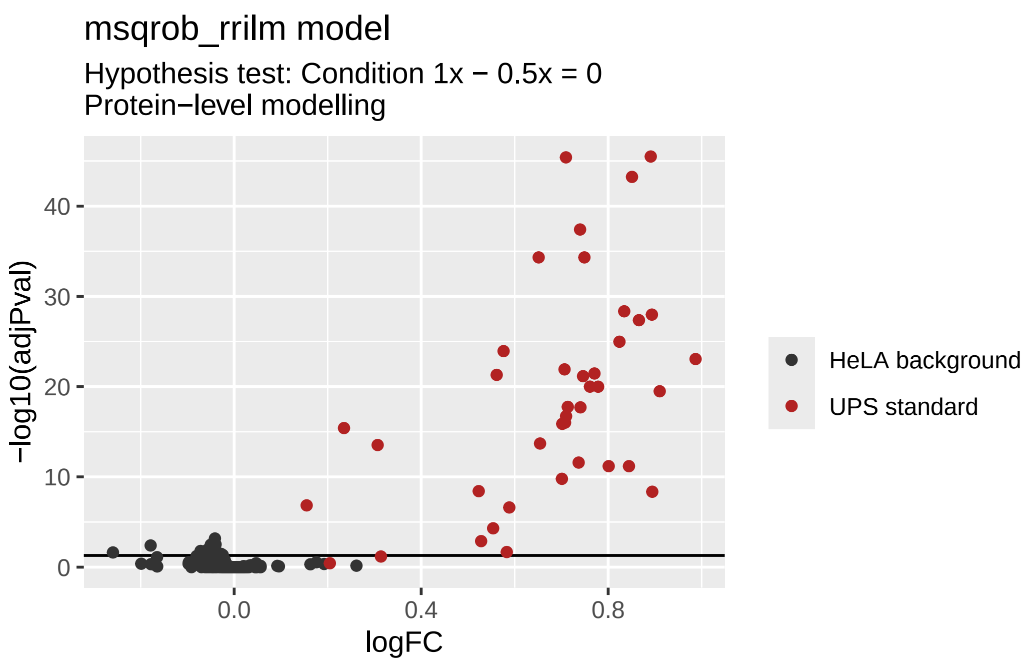

We plot the fold change distributions:

```
ggplot(htestProt) +
    aes(y = logFC,
        x = isUps,
        colour = isUps) +
    geom_boxplot() +
    geom_hline(yintercept = c(0, 1), colour = c("grey20", "firebrick")) +
    scale_color_manual(
        values = c("grey20", "firebrick"), name = "",
        labels = c("HeLA background", "UPS standard")
    ) +
    ggtitle("Distribution of the log2 fold changes",
            "Hypothesis test: Condition 1x - 0.5x = 0\nProtein-level modelling")
```

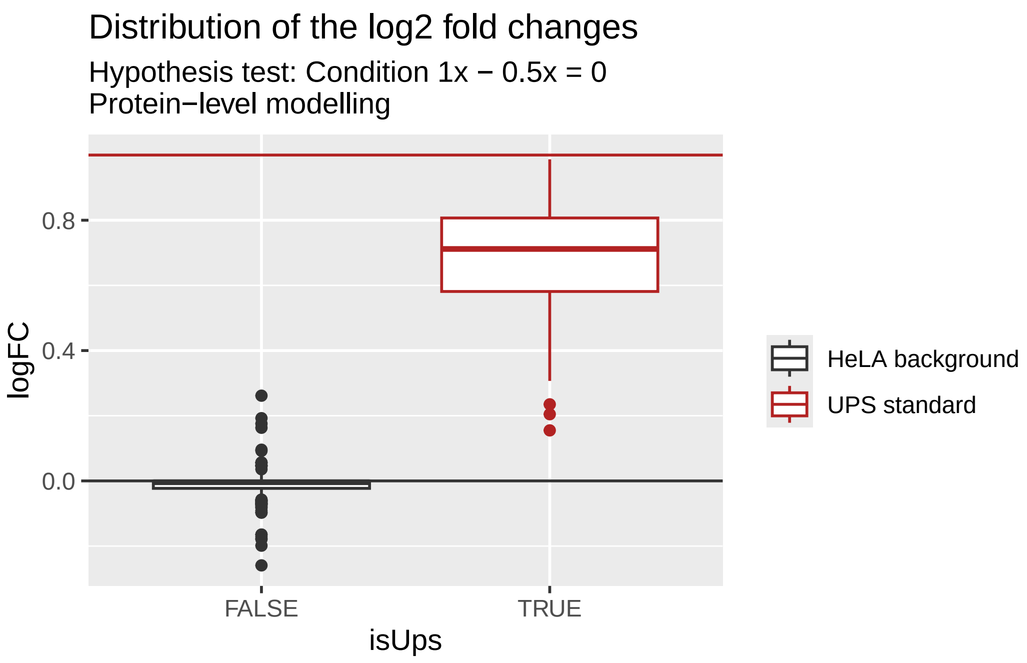

Exploring the intensities at the protein level is simplified compared to PSM-level exploration since every sample now contains a single observation, the protein intensity.

```
data.frame(intensity = assay(spikein[["proteins"]])[targetProtein, ],
           colData(spikein),
           colname = colnames(spikein[["proteins"]])) |>
    mutate(colname = factor(colname, levels = unique(colname[order(Condition)]))) |>
    ggplot() +
    aes(x = colname,
        y = intensity) +
    geom_point(aes(colour = Condition)) +
    facet_grid(~ Mixture, scales = "free") +
    ggtitle(targetProtein) +
    theme_minimal() +
    theme(axis.text.x = element_blank())
```

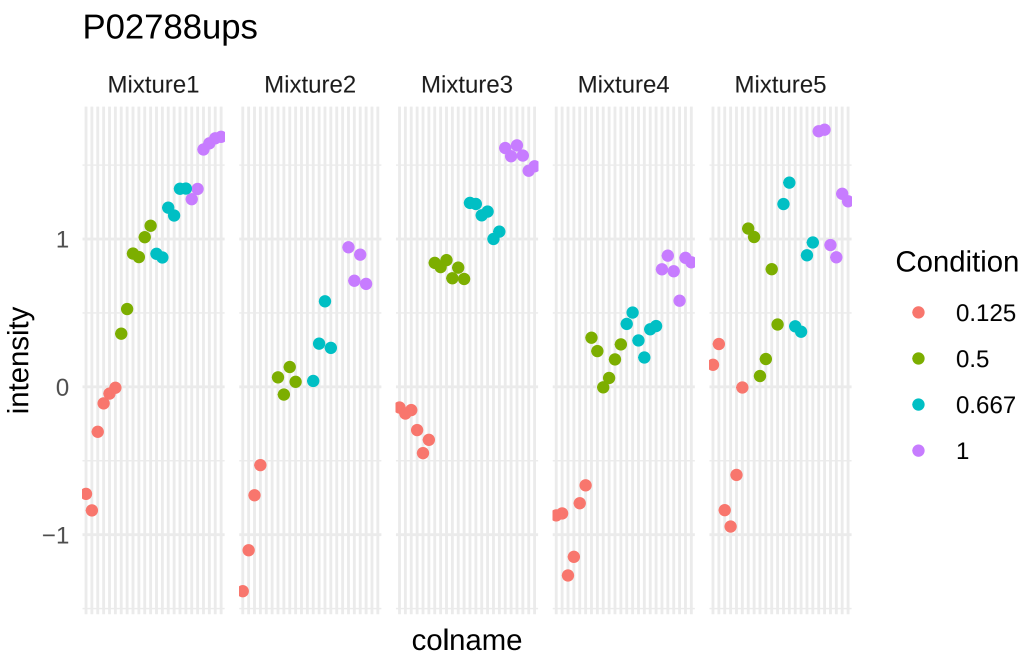

Notice how the summarisation-based approach hides the variation associated with the measurement of different peptide ions within the same protein, as well as discrepancies between peptide identification rates across mixtures.

Inference for all pairwise comparisons between conditions

For a factor variable, we often want to infer on all pairwise comparisons between the groups. Note, that our models have a fixed effect component that consist of a single factor and that we have chosen to suppress the intercept. `msqrob2` therefore returned models with a separate model parameter to estimate the mean for every group.

```
design_fixed  <- model.matrix(~ 0 + Condition, colData(spikein))
(param_names_fixed <- colnames(design_fixed))
```

```
[1] "Condition0.125" "Condition0.5"   "Condition0.667" "Condition1"
```

If we use ridge regression, `msqrob2` by default will put the prefix `ridge` to the parameter names of the fixed effects.

```
(param_names_fixed <- paste0("ridge", param_names_fixed))
```

```
[1] "ridgeCondition0.125" "ridgeCondition0.5"   "ridgeCondition0.667"
[4] "ridgeCondition1"
```

We now make all pairwise contrasts. The function `combn()` can make all combinations of a vector and we apply the function `paste()` with the argument `collapse = " - "` to these combinations. This makes a vector of strings specifying all contrasts of interest. Upon pasting "= 0" to each of the contrasts, all null hypotheses corresponding to the pairwise comparisons are specified.

Note, that we first sort the parameter names in decreasing order to ensure that a positive log2 fold change estimate refers to an upregulation in the highest spike-in condition involved in the comparison.

```
(hypotheses <- param_names_fixed |>
     sort(decreasing = TRUE) |>
     combn(2, paste, collapse=" - ") |>
     paste("= 0"))
```

```
[1] "ridgeCondition1 - ridgeCondition0.667 = 0"
[2] "ridgeCondition1 - ridgeCondition0.5 = 0"
[3] "ridgeCondition1 - ridgeCondition0.125 = 0"
[4] "ridgeCondition0.667 - ridgeCondition0.5 = 0"
[5] "ridgeCondition0.667 - ridgeCondition0.125 = 0"
[6] "ridgeCondition0.5 - ridgeCondition0.125 = 0"
```

We now have specified the null hypotheses for all pairwise contrasts of interest. Below, we will generate the contrast matrix for these six contrasts.

```
(L <- makeContrast(hypotheses, parameterNames = param_names_fixed))
```

```
                    ridgeCondition1 - ridgeCondition0.667
ridgeCondition0.125                                     0
ridgeCondition0.5                                       0
ridgeCondition0.667                                    -1
ridgeCondition1                                         1
                    ridgeCondition1 - ridgeCondition0.5
ridgeCondition0.125                                   0
ridgeCondition0.5                                    -1
ridgeCondition0.667                                   0
ridgeCondition1                                       1
                    ridgeCondition1 - ridgeCondition0.125
```

```
ridgeCondition0.125                                  -1
ridgeCondition0.5                                     0
ridgeCondition0.667                                   0
ridgeCondition1                                       1
                    ridgeCondition0.667 - ridgeCondition0.5
ridgeCondition0.125                                     0
ridgeCondition0.5                                      -1
ridgeCondition0.667                                     1
ridgeCondition1                                         0
                    ridgeCondition0.667 - ridgeCondition0.125
ridgeCondition0.125                                      -1
ridgeCondition0.5                                         0
ridgeCondition0.667                                       1
ridgeCondition1                                           0
                    ridgeCondition0.5 - ridgeCondition0.125
ridgeCondition0.125                                    -1
ridgeCondition0.5                                       1
ridgeCondition0.667                                     0
ridgeCondition1                                         0
```

The next step is to perform hypothesis tests for each contrast

```
spikein <- hypothesisTest(
    spikein, i = "proteins_msqrob",
    contrast = L,
    modelColumn = "msqrob_psm_rrilmm",
    overwrite = TRUE
)
```

Six columns have been added to the row data, one for each contrast.

```
colnames(rowData(spikein[["proteins_msqrob"]]))
```

```
 [1] "Checked"
 [2] "Confidence"
 [3] "Marked.as"
 [4] "X..Protein.Groups"
 [5] "X..Proteins"
 [6] "Master.Protein.Accessions"
 [7] "Master.Protein.Descriptions"
 [8] "Protein.Accessions"
```

```
 [9] "Protein.Descriptions"
[10] "Activation.Type"
[11] "MS.Order"
[12] "Quan.Info"
[13] "isUps"
[14] "nProtsMapped"
[15] "psmRank"
[16] ".n"
[17] "msqrob_psm_rrilmm"
[18] "ridgeCondition1 - ridgeCondition0.5"
[19] "ridgeCondition1 - ridgeCondition0.667"
[20] "ridgeCondition1 - ridgeCondition0.125"
[21] "ridgeCondition0.667 - ridgeCondition0.5"
[22] "ridgeCondition0.667 - ridgeCondition0.125"
[23] "ridgeCondition0.5 - ridgeCondition0.125"
```

The results of contrast `"ridgeCondition1 - ridgeCondition0.5"` can be retrieved as follows.

```
head(rowData(spikein[["proteins_msqrob"]])[,"ridgeCondition1 - ridgeCondition0.5"])
```

```
               logFC           se         df             t       pval
O00151 -2.564638e-02 2.151045e-02  473.82313 -1.192276e+00 0.23374996
O00220  1.574596e-02 5.935274e-02   94.62092  2.652946e-01 0.79135965
O00244 -3.846217e-11 1.737203e-06   84.20039 -2.214029e-05 0.99998239
O00299 -3.224517e-02 1.179043e-02 1379.86119 -2.734859e+00 0.00632058
O00330 -2.898931e-02 3.675144e-02  193.96129 -7.887940e-01 0.43119548
O00399            NA           NA         NA            NA         NA
          adjPval
O00151 0.56558630
O00220 1.00000000
O00244 1.00000000
O00299 0.03353122
O00330 0.87073669
O00399         NA
```

Note, the we can use the same code to perform the hypothesis tests and extract the results for the protein-level model.

```
spikein <- hypothesisTest(
    spikein, i = "proteins",
    contrast = L,
    modelColumn = "msqrob_rrilmm",
    overwrite = TRUE
)
```

```
head(rowData(spikein[["proteins"]])[,"ridgeCondition1 - ridgeCondition0.5"])
```

```
              logFC         se       df          t       pval    adjPval
O00151 -0.011923963 0.02048120 104.6714 -0.5821905 0.56169027 1.00000000
O00299 -0.030143423 0.01272432 104.1417 -2.3689607 0.01968154 0.11751036
O00410 -0.018771290 0.01074906 104.1756 -1.7463197 0.08370311 0.35399440
O00629  0.003201686 0.02101664 105.3579  0.1523406 0.87920969 1.00000000
O14745 -0.033068368 0.01491050 104.3857 -2.2177906 0.02873612 0.16203980
O14980 -0.026264096 0.01005398 104.1682 -2.6123091 0.01032205 0.07225436
```

Dealing with `fitErrors`

Missing value patterns in the data may lead to non-estimable parameters. This is recognised by `msqrob2` and will lead to `fitErrors` which is a type of model output where the model could not be fit. This information is available from the `StatModel` objects.

```
rowData(spikein[["proteins_msqrob"]])[["msqrob_psm_rrilmm"]] |>
    sapply(function(x) x@type) |>
    table()
```

```
fitError     lmer
      94      313
```

We suggest 3 strategies for dealing with these `fitErrors`.

Removing the random effect of sample

This strategy only applies for PSM-level models. Some proteins are difficult to detect and may be quantified by a single peptide ion species. In these cases, every sample contains a single observation for the protein and hence no random effect of `Run:Channel` can be estimated.

While the results for such one-hit wonders are questionable, we provide `msqrobRefit()` to refit a new model for a subset of proteins of interest.

Work in progress: we plan to include soon the function to perform the refitting in `msqrob2`. Below, you can find a prototype of such functionality. See `?msqrobAggregate` for documentation of the arguments.

```
msqrobRefit <- function(object, formula, i, subset, fcol, name,
                        modelColumnName, ...) {
    seti <- getWithColData(object, i)
    setj <- getWithColData(object, name)
    if (any(!subset %in% rowData(seti)[[fcol]]))
        stop("Some entries in 'subset' not found in '", fcol,
             "' (rowData of set '", i, "')")
    setjRefit <- msqrobAggregate(
        seti[rowData(seti)[[fcol]] %in% subset, ],
        formula = formula, fcol = fcol, modelColumnName = modelColumnName,
        ...
    )
    rowData(setj)[[modelColumnName]][subset] <-
        rowData(setjRefit)[[modelColumnName]][subset]
    modelsNew <- rowData(setj)[[modelColumnName]]
    hlp <- limma::squeezeVar(
        var = vapply(modelsNew, getVar, numeric(1)),
        df = vapply(modelsNew, getDF, numeric(1))
    )
    for (ii in seq_along(modelsNew)) {
        modelsNew[[ii]]@varPosterior <- as.numeric(hlp$var.post[ii])
        modelsNew[[ii]]@dfPosterior <- as.numeric(hlp$df.prior + getDF(modelsNew[[ii]]))
    }
    rowData(object[[name]])[[modelColumnName]] <- modelsNew
    object
}
```

In this case, we want to refit a model without a sample effect for one-hit-wonder proteins. This information can be retrieved from the aggregation results, using `aggcounts()`. This getter function provides the number of features used when performing summarisation for each protein in each sample.

```
counts <- aggcounts(spikein[["proteins_msqrob"]])
counts[1:5, 1:5]
```

```
        161117_SILAC_HeLa_UPS1_TMT10_Mixture1_01.raw_Abundance..127N
```

```
000151                                                       4
000220                                                       1
000244                                                       0
000299                                                      15
000330                                                       2
       161117_SILAC_HeLa_UPS1_TMT10_Mixture1_01.raw_Abundance..127C
000151                                                       4
000220                                                       2
000244                                                       0
000299                                                      15
000330                                                       2
       161117_SILAC_HeLa_UPS1_TMT10_Mixture1_01.raw_Abundance..128N
000151                                                       4
000220                                                       2
000244                                                       0
000299                                                      15
000330                                                       2
       161117_SILAC_HeLa_UPS1_TMT10_Mixture1_01.raw_Abundance..128C
000151                                                       4
000220                                                       1
000244                                                       0
000299                                                      15
000330                                                       2
       161117_SILAC_HeLa_UPS1_TMT10_Mixture1_01.raw_Abundance..129N
000151                                                       4
000220                                                       2
000244                                                       0
000299                                                      15
000330                                                       2
```

One-hit wonder proteins are proteins for which the number of feature used for summarisation does not exceed 1 peptide ion across samples.

```
oneHitProteins <- rownames(counts)[rowMax(counts) == 1]
```

Using `msqrobRefit()` is very similar to `msqrobAggregate()`, see here however that we adapted the formula to remove the random effect for channel nested within run. We also mention which proteins must be refit using the `subset` argument.

```
spikein <- msqrobRefit(
    spikein, i = "ions",
    subset = oneHitProteins,
```

```r
    formula = ~ 0 + Condition + ## fixed effect for experimental condition
        # (1 | Channel) + ## fixed effect for channel
        (1 | Mixture) + ## random effect for mixture
        (1 | Run ) + ## random effect for run
        (1 | Run:ionID), ## random effect for PSM nested in MS run
        ## random effect for channel nested in run has been removed
    fcol = "Protein.Accessions",
    modelColumnName = "msqrob_psm_rrilmm",
    name = "proteins_msqrob",
    robust = TRUE, ridge = TRUE
)
```

Let's see how removing the random effect of channel within run for one-hit-wonder proteins reduced the number of `fitError`s.

```r
fitTypes <- rowData(spikein[["proteins_msqrob"]])[["msqrob_psm_rrilmm"]] |>
    sapply(function(x) x@type)
table(fitTypes)
```

```
fitTypes
fitError     lmer
       1      406
```

Manual inspection

One protein is still non-estimable upon refitting and requires additional data exploration to understand why the model cannot be estimated. Let us take the protein that cannot be fitted.

```r
proteinError <- names(fitTypes[fitTypes == "fitError"])[[1]]
```

To understand the problem, we plot the data for that protein using the same `QFeatures` pipeline described above.

```r
ionData <- spikein[proteinError, , "ions"] |>
    longFormat(colvars = colnames(colData(spikein)),
               rowvars = c("Protein.Accessions", "ionID")) |>
    data.frame() |>
    filter(!is.na(value)) |>
    mutate(colname = factor(colname, levels = unique(colname[order(Condition)]))) #4
```

Hence, we here plot the data in function of the `Channel` (x-axis), `Condition` (colour) and `Mixture` (shape).

```
ggplot(ionData) +
    aes(x = colname,
        y = value) +
    geom_point(aes(colour = Condition)) +
    facet_grid(~ Mixture, scales = "free") +
    ggtitle(targetProtein) +
    theme_minimal() +
    theme(axis.text.x = element_blank())
```

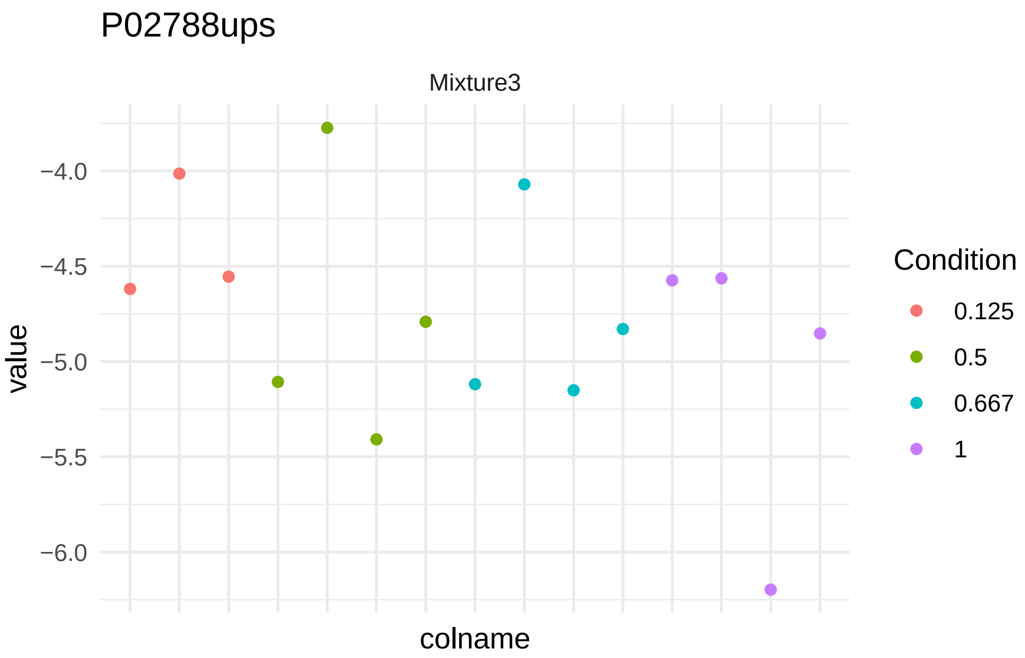

We can immediately spot that PSM intensities are only present in mixture 3. Hence, the mixed model cannot be fitted with a random effect for mixture. A solution would be drop the random effect for mixture, provided that a data analysis expert and/or a field specialist deems it reasonable. This requires expert intervention to simplify the model definition based on the available data set so as to provide valid statistical inference on the research hypotheses.

`msqrob2` flags these problematic proteins instead of defining ad-hoc heuristics, avoiding potentially misleading conclusions. The expert can then decide to refit a simpler model using `msqrobRefit()`.

Note, that the same approach can be applied for summarisation-based models that start from protein data.

```r
se <- getWithColData(spikein, "proteins")
data.frame(intensity = assay(se)[proteinError, ],
           colData(spikein),
           colname = colnames(se)) |>
    filter(!is.na(intensity)) |>
    mutate(colname = factor(colname, levels = unique(colname[order(Condition)]))) |>
    ggplot() +
    aes(x = colname,
        y = intensity) +
    geom_point(aes(colour = Condition)) +
    facet_grid(~ Mixture, scales = "free") +
    ggtitle(proteinError, "associated to a fitError\nSummarised protein intensities (median [illegible]
    theme_minimal() +
    theme(axis.text.x = element_blank())
```

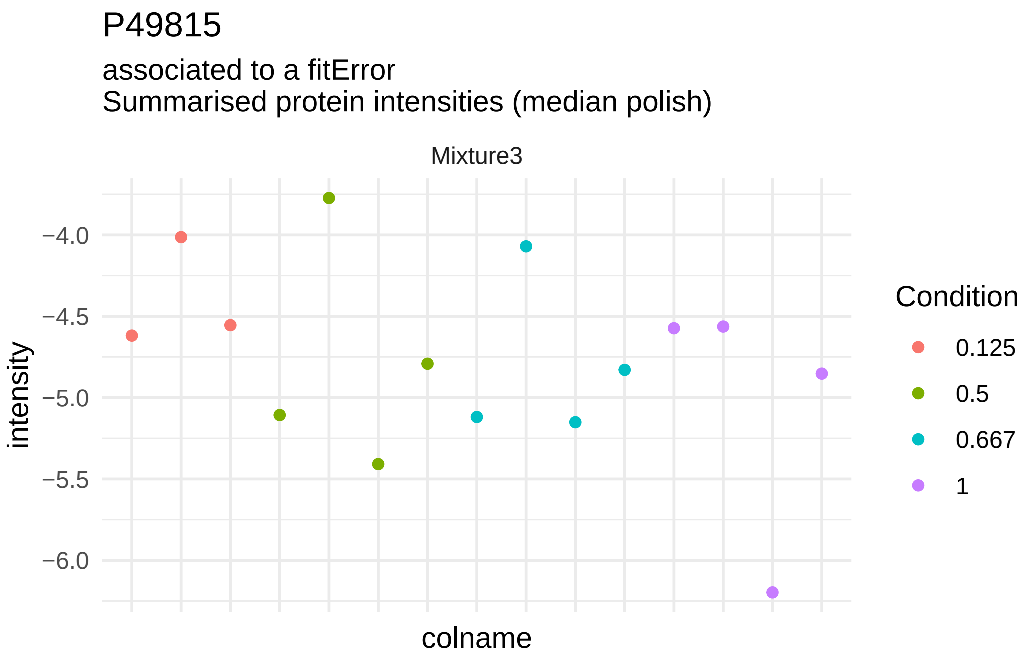

The same conclusion applies as for the PSM-level data. In fact, this is the same plot as above since the protein is also a one-hit wonder, what seriously questions the reliability of the data for protein. This example illustrates the relevance of `msqrob2` to safeguard against automatic model simplification that may be otherwise fall unnoticed by the user.

Imputation

The last strategy to deal with fit errors is to impute missing values so that all models can be estimated. `QFeatures` provides a large panel of imputation strategies through `impute()`. Identifying which imputation strategy is most suited for this data set is outside the scope of this vignette, and we here arbitrarily decide to use KNN imputation.

```
(spikein <- impute(
    spikein, i = "ions", name = "ions_imputed",
    method = "knn", colmax = 1
))
```

```
An instance of class QFeatures containing 64 assays:
 [1] Mixture1_01: SummarizedExperiment with 1649 rows and 8 columns
 [2] Mixture1_02: SummarizedExperiment with 1644 rows and 8 columns
 [3] Mixture1_03: SummarizedExperiment with 1703 rows and 8 columns
 ...
 [62] proteins: SummarizedExperiment with 407 rows and 120 columns
 [63] proteins_msqrob: SummarizedExperiment with 407 rows and 120 columns
 [64] ions_imputed: SummarizedExperiment with 4066 rows and 120 columns
```

The function added a new set `ions_imputed` which we can use to fit the PSM model.

```
spikein <- msqrobAggregate(
    spikein,  i = "ions_imputed",
    formula = ~ 0 + Condition + ## fixed effect for experimental condition
        # (1 | Channel) + ## random effect for channel
        (1 | Mixture) + ## random effect for mixture
        (1 | Run) + ## random effect for run
        (1 | Run:Channel) + ## random effect for PSMs from the same protein in a channel of
        (1 | Run:ionID), ## random effect for ions in the same spectrum of an MS run
    fcol = "Protein.Accessions",
    modelColumnName = "msqrob_psm_rrilmm",
    name = "proteins_msqrob_imputed",
    robust = TRUE, ridge = TRUE
)
```

We here assess how many models have been estimated for all proteins upon imputation.

```
rowData(spikein[["proteins_msqrob_imputed"]])[["msqrob_psm_rrilmm"]] |>
    sapply(function(x) x@type) |>
    table()
```

```
fitError     lmer
      59      348
```

Again `fitErrors` were generated for one-hit-wonder proteins.

```
counts <- aggcounts(spikein[["proteins_msqrob_imputed"]])
oneHitProteins <- rownames(counts)[rowMax(counts) == 1]
spikein <- msqrobRefit(
    spikein, i = "ions_imputed",
    subset = oneHitProteins,
    formula = ~ 0 + Condition + ## fixed effect for experimental condition
        # (1 | Channel) + ## random effect for channel
        (1 | Mixture) + ## random effect for mixture
        (1 | Run ) + ## random effect for run
        (1 | Run:ionID), ## random effect for PSM nested in MS run
        ## random effect for channel nested in run has been removed
    fcol = "Protein.Accessions",
    modelColumnName = "msqrob_psm_rrilmm",
    name = "proteins_msqrob_imputed",
    robust = TRUE, ridge = TRUE
)
rowData(spikein[["proteins_msqrob_imputed"]])[["msqrob_psm_rrilmm"]] |>
    sapply(function(x) x@type) |>
    table()
```

```
lmer
 407
```

Upon refit, no `fitErrors` were generated for any proteins, as expected. Be mindful that although this approach no longer requires strong statistical insights, the results upon imputation will be highly depended on the suitability of the imputation approach.

We can apply the same approach for upon summarisation, starting from the imputed peptide ion data.

```
spikein <- aggregateFeatures(
    spikein, i = "ions_imputed", name = "proteins_imputed",
    fcol = "Protein.Accessions", fun = MsCoreUtils::medianPolish,
    na.rm = TRUE
)
```

```
spikein <- msqrob(
    spikein,  i = "proteins_imputed",
    formula = ~ 0 + Condition + ## fixed effect for experimental condition
        # (1 | Channel) + ## random effect for channel
        (1 | Mixture) + ## random effect for mixture
        (1 | Run), ## random effect for MS run
    robust = TRUE, ridge = TRUE,
    modelColumnName = "msqrob_rrilmm",
    overwrite = TRUE
)
models <- rowData(spikein[["proteins_imputed"]])[["msqrob_rrilmm"]]
table(sapply(models, function(x) x@type))
```

```
lmer
 407
```

Conclusion

In this vignette, we have demonstrated how to run msqrob2TMT workflows. Because the packages relies on the `QFeatures` data class, we could demonstrate the implementation of a complete pre-processing workflow: sample filtering, PSM filtering, missing value management, log2-transformation, normalisation, (optionally) summarisation and (optionally) imputation.

Once pre-processed, we use the `msqrob2` package to model all sources of variability in the MS experiment: effect of treatment of interest, effect of TMT labelling, effect of the MS acquisition run, and the effect of replication. We built protein-level models, but we have also shown that we can build PSM-level models if we also include a spectrum effect and an effect for channel nested within run.

We showed how to run statistical inference on the modelling results to retrieve the significance of differentially abundant proteins. We explored statistical results through volcano plots and boxplots of the log2 fold changes and visually validated the results for one protein by plotting the input data. We have shown the inference pipeline is similar for both PSM-level models and protein-level models. Furthermore, we illustrated how to streamline the analysis of multiple hypothesis tests.

Finally, we have demonstrated how to deal with proteins that cannot be modelled due to missing values. For PSM-level models, we can remove the random effects for channel within run that cannot be estimated for one-hit-wonder proteins. We can also manually inspect how missing values can influence the model design, and refit a simplified model upon expert's

intervention. Finally, we can impute missing values, which unlocks model fitting, but imposes strong assumption on the validity of the imputation approach and the reliability of the predicted values.

Citation

Vandenbulcke S, Vanderaa C, Crook O, Martens L, Clement L. msqrob2TMT: robust linear mixed models for inferring differential abundant proteins in labelled experiments with arbitrarily complex design. bioRxiv. Published online March 29, 2024:2024.03.29.587218. doi:10.1101/2024.03.29.587218

License

This vignette is distributed under a Artistic-2.0 license.

Session info

```
R version 4.4.2 (2024-10-31)
Platform: x86_64-pc-linux-gnu
Running under: Ubuntu 24.04.1 LTS

Matrix products: default
BLAS:   /usr/lib/x86_64-linux-gnu/openblas-pthread/libblas.so.3
LAPACK: /usr/lib/x86_64-linux-gnu/openblas-pthread/libopenblasp-r0.3.26.so;  LAPACK version 3

locale:
 [1] LC_CTYPE=en_US.UTF-8       LC_NUMERIC=C
 [3] LC_TIME=en_US.UTF-8        LC_COLLATE=en_US.UTF-8
 [5] LC_MONETARY=en_US.UTF-8    LC_MESSAGES=en_US.UTF-8
 [7] LC_PAPER=en_US.UTF-8       LC_NAME=C
 [9] LC_ADDRESS=C               LC_TELEPHONE=C
[11] LC_MEASUREMENT=en_US.UTF-8 LC_IDENTIFICATION=C

time zone: Etc/UTC
tzcode source: system (glibc)

attached base packages:
[1] stats4    stats     graphics  grDevices utils     datasets  methods
[8] base
```

```
other attached packages:
 [1] BiocFileCache_2.14.0       dbplyr_2.5.0
 [3] BiocParallel_1.40.0        ggplot2_3.5.1
 [5] dplyr_1.1.4                msqrob2_1.14.1
 [7] QFeatures_1.16.0           MultiAssayExperiment_1.32.0
 [9] SummarizedExperiment_1.36.0 Biobase_2.66.0
[11] GenomicRanges_1.58.0       GenomeInfoDb_1.42.1
[13] IRanges_2.40.1             S4Vectors_0.44.0
[15] BiocGenerics_0.52.0        MatrixGenerics_1.18.1
[17] matrixStats_1.5.0

loaded via a namespace (and not attached):
 [1] Rdpack_2.6.2            DBI_1.2.3               rlang_1.1.4
 [4] magrittr_2.0.3          clue_0.3-66             compiler_4.4.2
 [7] RSQLite_2.3.9           vctrs_0.6.5             reshape2_1.4.4
[10] stringr_1.5.1           ProtGenerics_1.38.0     pkgconfig_2.0.3
[13] crayon_1.5.3            fastmap_1.2.0           XVector_0.46.0
[16] labeling_0.4.3          rmarkdown_2.29          UCSC.utils_1.2.0
[19] nloptr_2.1.1            purrr_1.0.2             bit_4.5.0.1
[22] xfun_0.50               zlibbioc_1.52.0         cachem_1.1.0
[25] jsonlite_1.8.9          blob_1.2.4              DelayedArray_0.32.0
[28] parallel_4.4.2          cluster_2.1.8           R6_2.5.1
[31] stringi_1.8.4           limma_3.62.2            boot_1.3-31
[34] Rcpp_1.0.14             knitr_1.49              BiocBaseUtils_1.8.0
[37] Matrix_1.7-1            splines_4.4.2           igraph_2.1.3
[40] tidyselect_1.2.1        abind_1.4-8             yaml_2.3.10
[43] codetools_0.2-20        curl_6.1.0              lattice_0.22-6
[46] tibble_3.2.1            plyr_1.8.9              withr_3.0.2
[49] evaluate_1.0.3          pillar_1.10.1           BiocManager_1.30.25
[52] filelock_1.0.3          reformulas_0.4.0        generics_0.1.3
[55] munsell_0.5.1           scales_1.3.0            minqa_1.2.8
[58] BiocStyle_2.34.0        glue_1.8.0              lazyeval_0.2.2
[61] tools_4.4.2             lme4_1.1-36             grid_4.4.2
[64] impute_1.80.0           tidyr_1.3.1             rbibutils_2.3
[67] MsCoreUtils_1.18.0      colorspace_2.1-1        nlme_3.1-166
[70] GenomeInfoDbData_1.2.13 cli_3.6.3               S4Arrays_1.6.0
[73] AnnotationFilter_1.30.0 gtable_0.3.6            digest_0.6.37
[76] SparseArray_1.6.0       farver_2.1.2            memoise_2.0.1
[79] htmltools_0.5.8.1       lifecycle_1.0.4         httr_1.4.7
[82] statmod_1.5.0           bit64_4.5.2             MASS_7.3-64
```
